# Semi-Synthesis of Marine-Derived Ilamycin F Derivatives and Their Antitubercular Activities

**DOI:** 10.3389/fchem.2021.774555

**Published:** 2021-10-29

**Authors:** Jun Li, Zhiyong Liu, Mingye Hong, Changli Sun, Tianyu Zhang, Hua Zhang, Jianhua Ju, Junying Ma

**Affiliations:** ^1^ CAS Key Laboratory of Tropical Marine Bio-Resources and Ecology, Guangdong Key Laboratory of Marine Materia Medica, RNAM Center for Marine Microbiology, South China Sea Institute of Oceanology, Chinese Academy of Sciences, Guangzhou, China; ^2^ Southern Marine Science and Engineering Guangdong Laboratory (Guangzhou), Guangzhou, China; ^3^ Tuberculosis Research Laboratory, State Key Laboratory of Respiratory Disease, Guangdong-Hong Kong-Macao Joint Laboratory of Respiratory Infectious Diseases, Guangzhou Institutes of Biomedicine and Health, Chinese Academy of Sciences, Guangzhou, China; ^4^ Guangdong Provincial Key Laboratory of Medical Molecular Diagnostics, Institute of Laboratory Medicine, Guangdong Medical University, Dongguan, China; ^5^ College of Oceanology, University of Chinese Academy of Sciences, Qingdao, China

**Keywords:** streptomycetes, cyclopeptide, antitubercular activity, ilamycin, semi-synthesis, derivatization

## Abstract

Tuberculosis (TB) is still a global disease threatening people’s lives. With the emergence of multi-drug-resistant *Mycobacterium tuberculosis* the prevention and control of tuberculosis faces new challenges, and the burden of tuberculosis treatment is increasing among the world. Ilamycins are novel cyclopeptides with potent anti-TB activities, which have a unique target protein against *M. tuberculosis* and drug-resistant strains. Herein, ilamycin F, a major secondary metabolite isolated from the marine-derived mutant strain *Streptomyces atratus* SCSIO ZH16 Δ*ilaR*, is used as a scaffold to semi-synthesize eighteen new ilamycin derivatives (ilamycin NJL1–NJL18, **1**–**18**). Our study reveals that four of ilamycin NJLs (**1, 6, 8**, and **10**) have slightly stronger anti-TB activities against *Mtb* H37Rv (minimum inhibitory concentration, 1.6–1.7 μM) compared with that of ilamycin F on day 14th, but obviously display more potent activities than ilamycin F on day 3rd, indicating anti-TB activities of these derivatives with fast-onset effect. In addition, cytotoxic assays show most ilamycin NJLs with low cytotoxicity except ilamycin NJL1 (**1**). These findings will promote the further exploration of structure-activity relationships for ilamycins and the development of anti-TB drugs.

## Introduction

Tuberculosis (TB) is an infectious disease caused by the pathogen *Mycobacterium tuberculosis* (*Mtb*), which is the leading cause of death from a single infectious agent. Globally, it is estimated that 10 million new cases and 1.2 million deaths occurred in 2019 due to TB infections (World Health Organization, 2020). With the appearance of drug-resistant strains, multidrug resistance (MRD) TB and extensive drug resistance (XDR) TB have resulted in a major challenge to the prevention and treatment of TB in the world, especially in developing countries (Saravanan et al., 2018). Standard treatment of TB is a long course, including a 2-months induction phase and a 4-months consolidation phase, thus it is important that drugs with fast-onset action can contribute to shorten treatment in clinical trials (Horsburgh et al., 2015). Hence, there is an urgent demand for development of novel anti-TB drugs with unique targets and fast-onset action.

**FIGURE 1:**
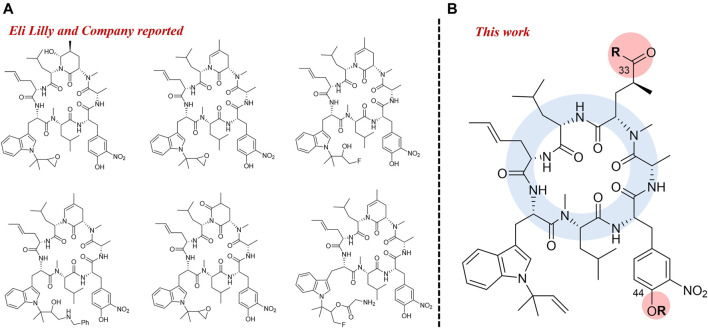
Semi-synthesis of rufomycin active derivatives from Eli Lilly and Company **(A)** Modified position from this work **(B)**.

With the development of drug-resistant tuberculosis, the discovery of new drugs or the drug-repurposed for tuberculosis is increasing recently (Furin et al., 2019). Ilamycins, also named rufomycins, comprise a representative of cycloheptapeptides with strong anti-TB activity, which were isolated from *Streptomyces atratus* and *S. islandicus* (Takita et al., 1962; Cary et al., 1971; Ma et al., 2017; Sun et al., 2020; Zhou et al., 2020). Previous studies identified the target of rufomycins, caseinolytic protein C1 (ClpC1), which was different from that of the current therapeutic drugs (Sassetti et al., 2003; Lee et al., 2016; Choules et al., 2018; Choules et al., 2019; Wolf et al., 2019). Therefore, compounds of this family are a promising drug-lead for the treatment of MRD- and XRD-TB. Moreover, for the structure-activity relationship (SAR) studies, Eli Lilly and Company firstly synthesized a series of ilamycin derivatives in 2000 (Lambooy, 2000). Anti-TB assays showed that 6 of the derivatives exhibited strong inhibitory activities against *Mtb* H37Ra (Figure 1A).

In recent years, our group has been focusing on the discovery and the biosynthesis of anti-infective antibiotics. Ilamycin F, isolated from a genetic engineered mutant of the deep South China Sea-derived strain *Streptomyces atratus* SCSIO ZH16, had a strong anti-TB activity against *M. tuberculosis* H37Rv with minimum inhibitory concentration (MIC) value of 1.2 μM (Ma et al., 2017). As the main metabolite of the mutant strain *S. atratus* SCSIO ZH16 Δ*ilaR,* the yield of ilamycin F is about 400–500 mg/L in its mutant. In this regard, ilamycin F is ideally utilized as a starting material for preparing new ilamycin derivatives, which will facilitate to further investigate their SAR and discover more efficient anti-TB drug leads. Herein, we report the preparation and characterization of eighteen new ilamycin F derivatives (ilamycin NJL1**–**NJL18) on C-33 and C-44 of ilamycin F (Figure 1B). Several semi-synthesized derivatives display potent anti-TB activity against *M. tuberculosis* H37Rv with fast-onset effect and low cytotoxicity.

## Results and Discussion

### Semi-Synthesis of Ilamycin NJLs

**SCHEME 1:**
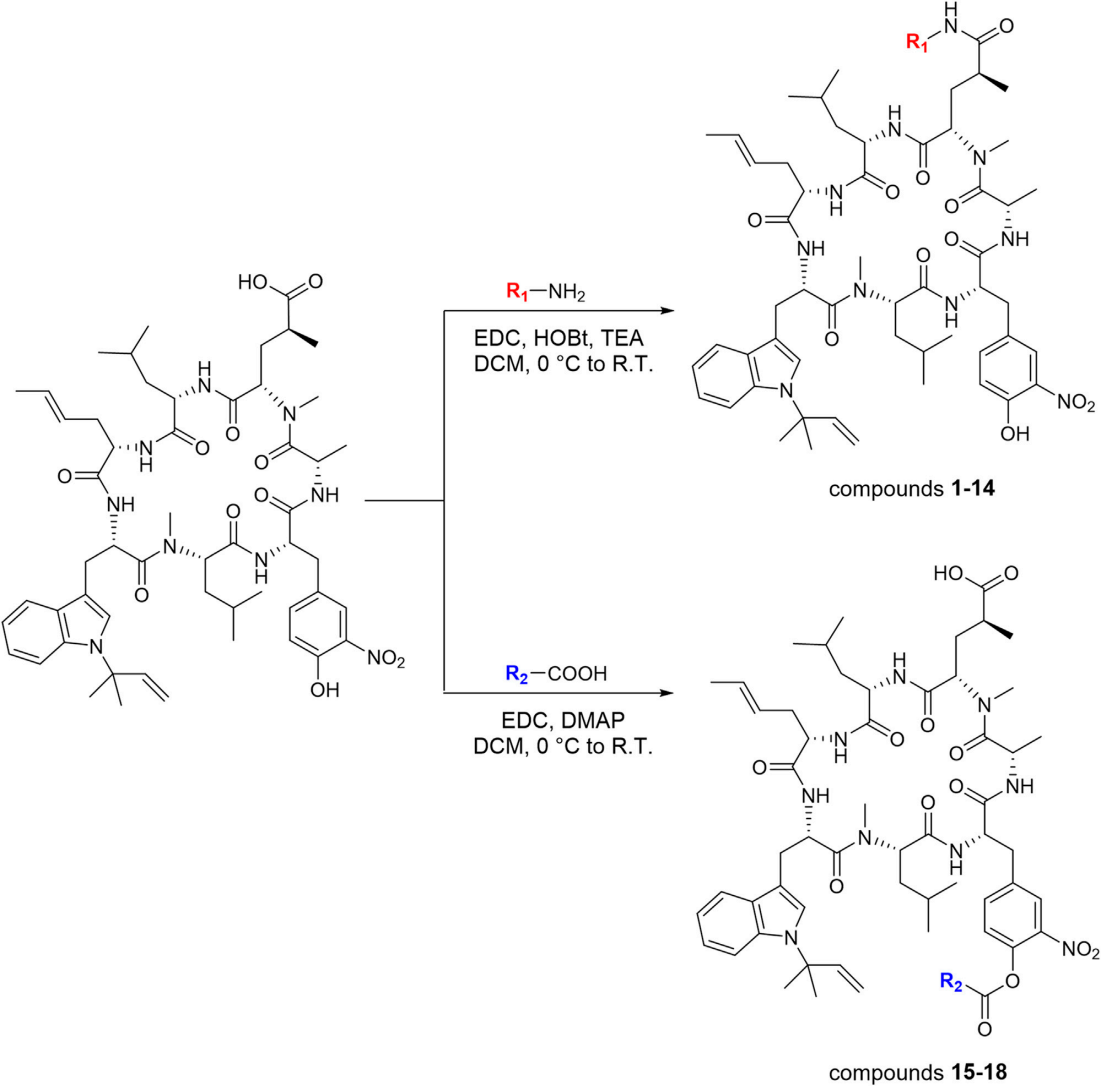
Synthetic routes of ilamycin NJLs (compounds **1**–**18**).

**FIGURE 2:**
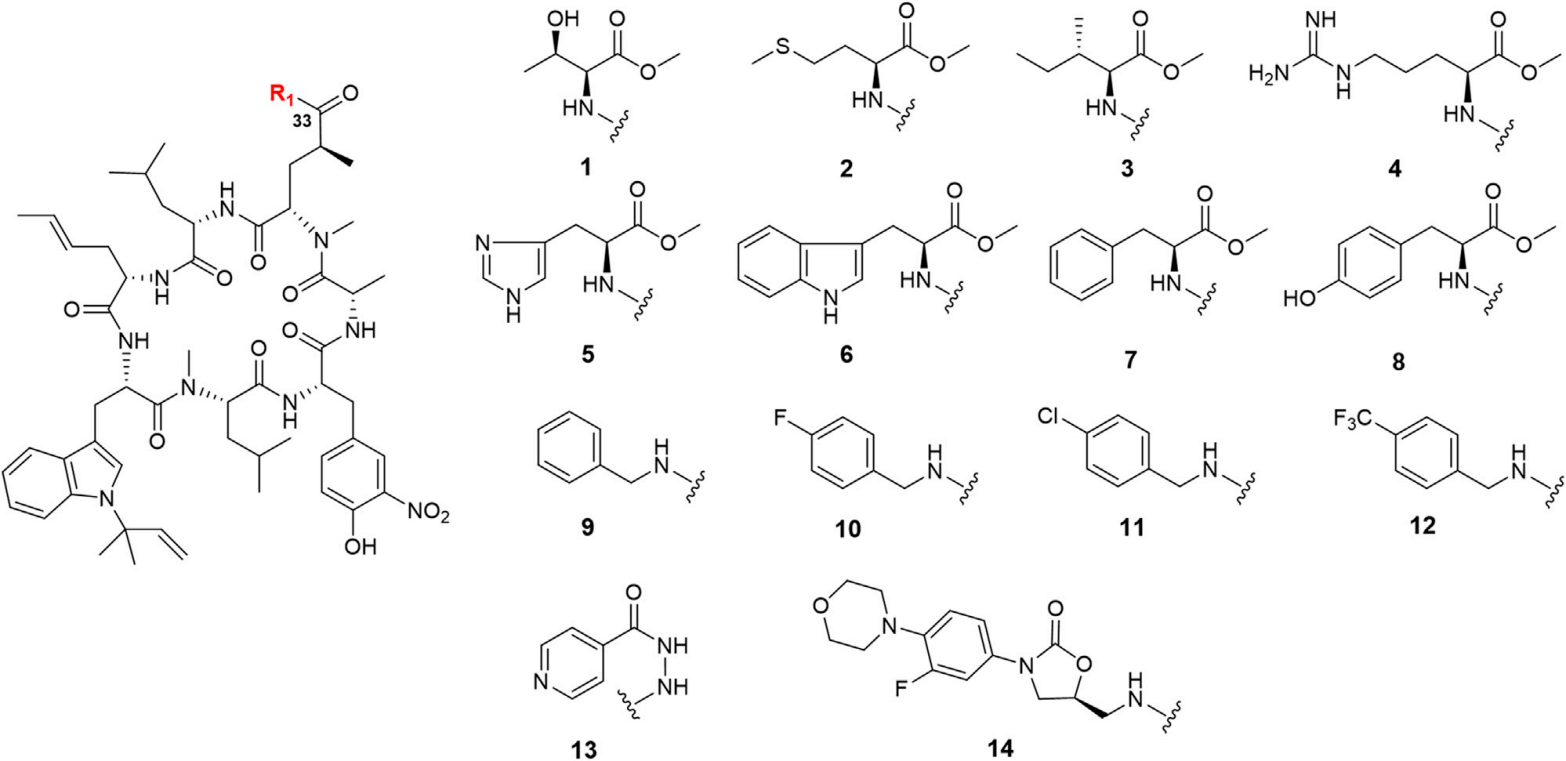
Structures of ilamycin NJLs modified at C-33 (compounds **1**–**14**).

Ilamycin F has two types of functional groups for modification, C-33 carboxyl group and C-44 hydroxyl group. With the aim of synthesizing new derivatives, several modifications in ilamycin F were introduced by acylation and esterification (Scheme 1). Ilamycin NJL1–NJL12 (**1**–**12**) were concisely synthesized by 1-(3-dimethylaminopropyl)-3-ethylcarbodiimide hydrochloride (EDC)/1-hydroxybenzotriazole (HOBt) assisted amidation at C-33 carboxyl group using l-amino acid methyl esters and benzylamine derivatives (Figure 2). The yields ranged from 74 to 83%. Moreover, according to the twin drug strategy (Contreras et al., 2008; Ibacache et al., 2018), isoniazid and N-deacetyl-linezolid, two anti-TB substrates, were respectively coupled with ilamycin F to generate ilamycin NJL13–NJL14 (**13**–**14**) using the aforementioned amidation method in 76–77% yield (Figure 2). The strategy was proposed to produce synergic effect by binding two targets.

**FIGURE 3:**
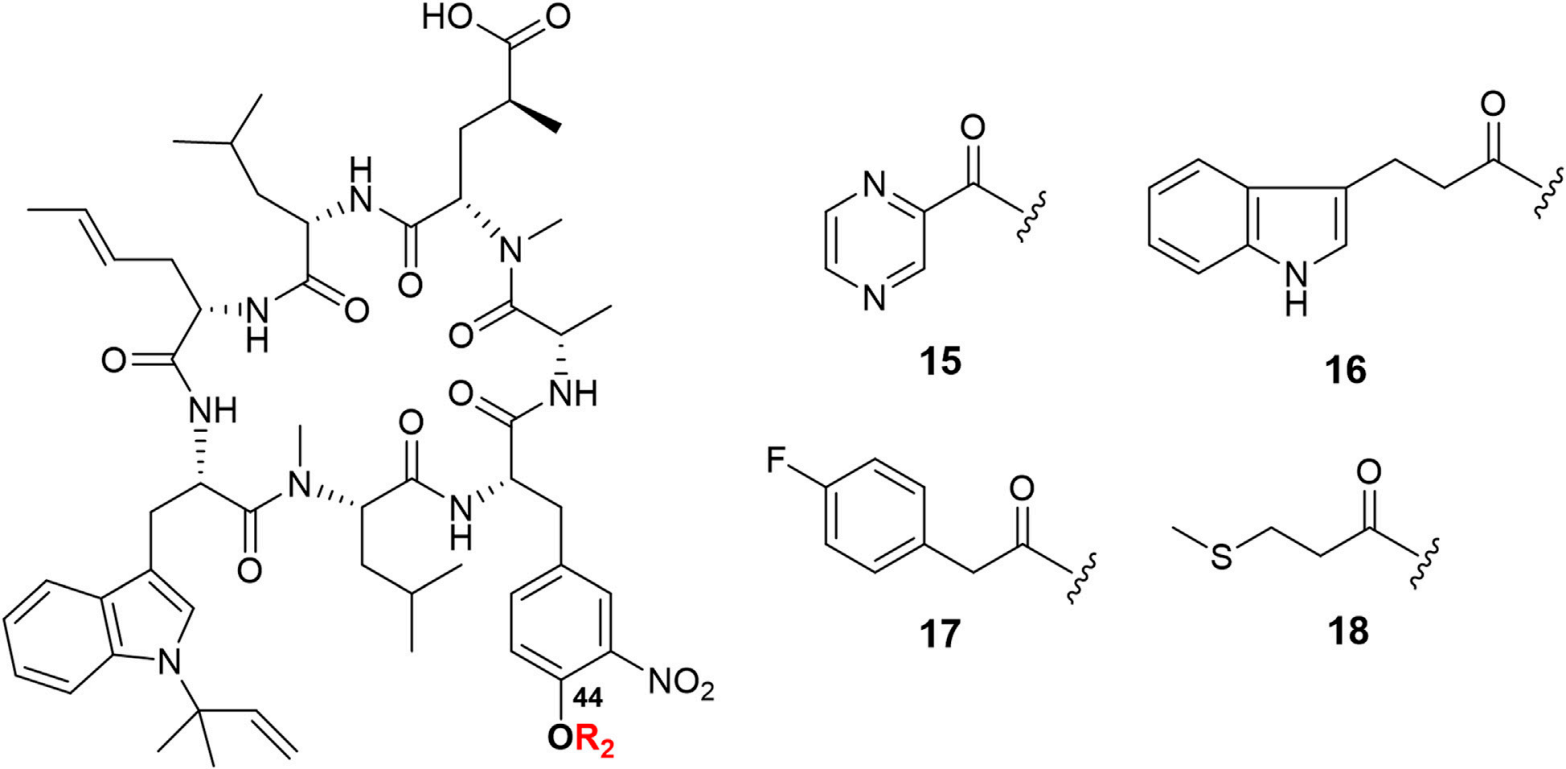
Structures of ilamycin NJLs modified at C-44 (compounds **15**–**18**).

The C-44 hydroxyl group of ilamycin F is another position for derivatization. Although various ether and aliphatic sidechains at C-44 were created through etherification or esterification by Eli Lilly and Company, all derivatives displayed low anti-TB activities (Lambooy, 2000). To further study SAR of ilamycin F, heteroaromatic rings, *p*-fluorophenylacetic acid and 3-(methylthio) propionic acid were introduced at the C-44 hydroxyl group (Scheme 1). Ilamycin NJL15–NJL18 (**15**–**18**) could be successfully obtained in the presence of EDC and 4-dimethylaminopyridine (DMAP) (Figure 3), but owing to low nucleophilicity of the C-44 hydroxyl group, the yields of the reaction were only given to 32**–**40%.

### Bioactivities of Ilamycin NJLs

The anti-TB activities of ilamycin NJL1–NJL18 (**1**–**18**) were evaluated against *M. tuberculosis* H37Rv, which has pathogenic and still popularly used in virulent laboratory (Camus et al., 2002; Målen et al., 2011). As depicted in Table 1, although basic amino acid derivatives (compounds **4–5**) at the C-33 had weak activities, the modification of neutral amino acid derivatives, in comparison with ilamycin F, displayed potent anti-TB activities with fast-onset effect. Compounds **1**–**3**, **6**, and **8**, showed efficient activities on day 3rd, which was higher than that of ilamycin F with 4–19 folds. Importantly, compounds **1, 6**, and **8** had slightly stronger activities than that of ilamycin F on day 14th (MIC, 1.6–1.7 μM), speculating that their modification did not change the interaction with targets, but would obviously facilitate to promoting penetration of membranes in *Mtb*. Moreover, benzylamine derivatives of ilamycin F, compounds **9**–**11**, were also exhibited the fast-onset effect on day 3rd except compound **12**. This finding indicated that benzylamine modified with a larger substituent significantly affected anti-TB activity of ilamycin derivatives. Although showed a similar activity with that of ilamycin F on day 14th, compound **10** was 2-fold more potent MIC value than that of **9** and **11**, which might result from the promotion of its lipophilicity by fluorine substituent and improved the penetration to cell membranes (Smart, 2001; Purser et al., 2008). However, the activities of compounds **13**–**14** were significantly decreased under the twin drug strategy, when a carboxyl group at the C-33 was replaced by isoniazid or N-deacetyl-linezolid. This result indicated that the construction of compounds **13**–**14** affected the binding to their targets, and exhibited no synergistic effect on anti-TB activity. Additionally, compounds **15**–**18** modified at the C-44 also showed lower activities. The similar groups coupling at the C-33 with beneficial effects could not produce the same promotion at the C-44 hydroxyl group of ilamycin F. The results suggested that the hydroxyl group at C-44 might serve as a pharmacophore, which was critical in achieving anti-TB activity and also consistence with our previous discoveries (Sun et al., 2020).

**TABLE 1:** Anti-tubercular activity of ilamycin NJLs (**1**–**18**) against *M. tuberculosis* H37Rv.

Compounds	MICs (μM)against H37Rv			Compounds	MICs (μM)against H37Rv		
	Day 3rd	Day 7th	Day 14th		Day 3rd	Day 7th	Day 14th
**1**	3.5	1.7	1.7	11	3.4	3.4	3.4
**2**	6.7	6.7	3.4	12	>100	>100	>100
**3**	6.8	6.8	6.8	13	27.6	27.6	27.6
**4**	26.4	26.4	26.4	14	48.5	48.5	48.5
**5**	26.8	26.8	26.8	15	27.9	27.9	27.9
**6**	1.7	1.7	1.7	16	26.4	26.4	26.4
**7**	26.6	13.3	6.6	17	27.2	27.2	27.2
**8**	1.6	1.6	1.6	18	28.0	28.0	28.0
**9**	3.5	3.5	3.5	ilamycin F	30.7	15.4	1.9
**10**	1.7	1.7	1.7	—			

To evaluate the application potential of these compounds, the cytotoxicity of ilamycin NJLs (**1**–**18**) was evaluated *in vitro* using five human cancer cell lines, including breast adenocarcinoma (MCF-7), cervical carcinoma (HeLa), hepatocellular carcinoma (HepG2), lung cancer (A549), colon cancer (HCT116); two normal cell lines including human hepatic cell line (L02) and human umbilical vein endothelial cell line (Huvec-12). Although most compounds (**2**–**18**) showed no or weak cytotoxicity, compound **1** exhibited a moderate IC_50_ value (5.7–9.0 μM) against MCF-7, A549, HCT116, and L02, which had a 3–8-fold promotion compared with that of ilamyicn F (Table 2). The result indicated that threonine methyl ester modified in C-33 carboxyl group of ilamycin F was favorable for the cytotoxic activity.

**TABLE 2:** IC_50_ value (μM) of ilamycin NJLs (**1**–**18**) against five human cancer cell lines and two normal cell lines.

Compounds	MCF-7	HeLa	HepG2	A549	HCT116	L02	Huvec-12
**1**	9.0	11.3	17.7	5.7	7.2	6.5	10.3
**2**	>50	>50	>50	>50	>50	>50	>50
**3**	>50	14.5	>50	>50	>50	12.0	>50
**4**	>50	26.0	>50	23.0	30.6	25.9	35.2
**5**	>50	>50	>50	>50	>50	>50	>50
**6**	>50	>50	>50	>50	>50	>50	>50
**7**	>50	>50	>50	>50	>50	>50	>50
**8**	>50	>50	>50	>50	>50	>50	>50
**9**	>50	>50	>50	>50	>50	>50	>50
**10**	>50	>50	>50	>50	>50	>50	>50
**11**	>50	>50	>50	>50	>50	>50	>50
**12**	>50	>50	>50	>50	>50	>50	>50
**13**	>50	>50	>50	>50	>50	>50	>50
**14**	>50	>50	>50	>50	>50	>50	>50
**15**	>50	>50	>50	>50	>50	>50	>50
**16**	>50	>50	>50	>50	>50	>50	>50
**17**	>50	>50	>50	>50	>50	>50	>50
**18**	>50	>50	>50	>50	>50	>50	>50
ilamycin F	32.2	31.0	>50	47.0	44.8	43.9	46.1
doxorubicin	4.0	0.7	0.6	1.5	4.0	7.7	12.0

## Conclusion

In this study, ilamycin F, a starting material isolated from marine-derived mutant *S. atratus* ZH16 Δ*ilaR*, was employed to semi-synthesize eighteen ilamycin F derivatives (ilamcyin NJL1–NJL18). Their inhibitory effects on *M. tuberculosis* H37Rv were tested *in vitro*. Our study revealed that compounds **1**, **6**, **8**, and **10** exhibited slightly stronger anti-TB activity (1.6–1.7 μM) with that of ilamycin F on day 14th, but displayed a 9**–**19-fold increased anti-TB activities compared with that of ilamycin F on day 3rd (MICs 1.6–3.5 μM), which indicated their rapid suppression effect on *M. tuberculosis*. In addition, most ilamycin NJLs had low cytotoxicity except compound **1** displayed a moderate cytotoxic activity (IC_50_, 5.7–11.3 μM) against five human cancer cell lines and two normal cell lines. Our results will be beneficial to further exploration for SAR of ilamycins and promote the development of anti-TB drugs.

## Data Availability

The original contributions presented in the study are included in the article/Supplementary Material, further inquiries can be directed to the corresponding author.
