# Supplementary material for: Semi-Synthesis of Marine-Derived Ilamycin F Derivatives and Their Antitubercular Activities
Source: Front Chem. 2021 Oct 29;9:774555. doi: 10.3389/fchem.2021.774555 (PMC8586704; doi:10.3389/fchem.2021.774555)
Supplement: Supplementary file 1 [file DataSheet1.pdf]

frontiers

Supplementary Material

Semi-synthesis of marine-derived ilamycin F derivatives and their antitubercular activities

Jun Li [1, 5], Zhiyong Liu [3], Mingye Hong [4], Changli Sun [1, 5], Tianyu Zhang [3], Hua Zhang [4], Jianhua Ju [1, 2, 5] and Junying Ma [1, 2, 5 *]

[1]CAS Key Laboratory of Tropical Marine Bio-Resources and Ecology, Guangdong Key Laboratory of Marine Materia Medica, RNAM Center for Marine Microbiology, South China Sea Institute of Oceanology, Chinese Academy of Sciences, Guangzhou, China, [2]Southern Marine Science and Engineering Guangdong Laboratory (Guangzhou), Guangzhou, China, [3]Tuberculosis Research Laboratory, State Key Laboratory of Respiratory Disease, Guangdong-Hong Kong-Macao Joint Laboratory of Respiratory Infectious Diseases, Guangzhou Institutes of Biomedicine and Health, Chinese Academy of Sciences, Guangzhou, China, [4]Guangdong Provincial Key Laboratory of Medical Molecular Diagnostics, Institute of Laboratory Medicine, Guangdong Medical University, Dongguan, China, [5]College of Oceanology, University of Chinese Academy of Sciences, Qingdao, China

*** Correspondence:**
Junying Ma
majunying@scsio.ac.cn

SUMMARY

General Information **1**

Fermentation and Isolation of Ilamycin F **1**

Antitubercular Activity Assay **1**

Cytotoxicity Assay **1**

General Procedure for Ilamycin NJL1–NJL14 **2**

General Procedure for Ilamycin NJL15–NJL18 **2**

References **12**

Spectra Data **13**

1D and 2D NMR spectrum of compound **1** **Figures S1-3**

1D and 2D NMR spectrum of compound **2** **Figures S4-6**

1D and 2D NMR spectrum of compound **3** **Figures S7-9**

1D and 2D NMR spectrum of compound **4** **Figures S10-12**

1D and 2D NMR spectrum of compound **5** **Figures S13-15**

1D and 2D NMR spectrum of compound **6** **Figures S16-18**

1D and 2D NMR spectrum of compound **7** **Figures S19-21**

1D and 2D NMR spectrum of compound **8** **Figures S22-24**

1D and 2D NMR spectrum of compound **9** **Figures S25-27**

1D and 2D NMR spectrum of compound **10** **Figures S28-30**

1D and 2D NMR spectrum of compound **11** **Figures S31-33**

1D and 2D NMR spectrum of compound **12** **Figures S34-36**

1D and 2D NMR spectrum of compound **13** **Figures S37-39**

1D and 2D NMR spectrum of compound **14** **Figures S40-42**

1D and 2D NMR spectrum of compound **15** **Figures S43-45**

1D and 2D NMR spectrum of compound **16** **Figures S46-48**

1D and 2D NMR spectrum of compound **17** **Figures S49-51**

1D and 2D NMR spectrum of compound **18** **Figures S52-54**

High-resolution mass spectra of compounds **1-18** **Figures S55-72**

General Information

All the reagents for synthesis were commercially available and used without further purification. NMR spectra were recorded in CD_3OD with an Avance 700 spectrometer (Bruker, Germany). High-resolution mass spectra were obtained with a MaXis quadrupole-time-of-flight mass spectrometer (Bruker, Germany). Optical rotations were detected by an MCP-500 polarimeter (Anton Paar) in methanol or chloroform at 25 °C. The progress of all reaction was monitored by TLC on silica gel 60 F254 plates (Merck, Germany). Semi-preparative HPLC were performed on a Hitachi Primaide 1430 with a diode array detector (DAD) and a YMC-Pack ODS-A column (250 × 20 mm, 5 μm), at a flow rate of 2.5 mL/min.

Fermentation and Isolation of Ilamycin F

The fermentation procedure of the mutant strain was described before (Sun et al., 2020). In brief, SCSIO ZH16 Δ*ilaR* was cultured onto YMS plates at 28 °C for 7 days to obtain matured spores. Seed cultures of SCSIO ZH16 Δ*ilaR* were grown in modified-AM2ab medium and incubated on a shaker at 200 rpm and 28 °C for 60 h, and then inoculated into modified-AM3 medium before cultured at 200 rpm and 30 °C for 7 days.

Antitubercular Activity Assay

The assay was performed as previously described (Zhang et al., 2012). Briefly, a preliminary test of anti-TB activity was conducted to determine the inhibitory effects of compounds **1**–**18** against selectable marker-free auto-luminescent *M. tuberculosis* H37Rv (UAlRv) using a broth dilution method. Subsequently, compounds **1**–**18** and ilamycin F were dissolved in dimethyl sulfoxide (DMSO) and serially diluted to 0.0078–128 μg/mL for testing with 7H9 broth (added with 0.2% glycerol and 0.05% Tween 80) to evaluate their MIC values. DMSO in 7H9 broth was used as negative control. All experiments were performed in triplicate.

Cytotoxicity Assay

The cytotoxicity assay was conducted using the standard CCK-8 method (Xu et al., 2017). Compound **1**–**18** and ilamycin F were evaluated for cytotoxicity using five human cancer cell lines and two normal human cell lines. All experiments were performed in triplicate and IC_{50} values were determined employing GraphPad Prism 5.0 software.

General Procedure for Ilamycin NJL1–NJL14

A solution of ilamycin F (20 mg, 1.0 equiv.) in dichloromethane (2 mL) was added the corresponding L-amino acid ester, benzylamine derivative, isoniazide or N-deacetyl-linezolid (1.2 equiv.). 1-(3-dimethylaminopropyl)-3-ethylcarbodiimide hydrochloride (EDC, 4.8 mg, 1.3 equiv.), 1-hydroxybenzotriazole (HOBt, 3.6 mg, 1.3 equiv.) and triethylamine (3.6 μL, 1.3 equiv.) were added with stirring at 0 °C. The reaction mixture was allowed to warm to room temperature for 4 h and then diluted with dichloromethane, washed with brine, dried over anhydrous Na_2SO_4 and evaporated under reduced pressure. The crude product was purified by semi-preparative HPLC (7:3 v/v MeCN-H_2O and 0.1% formic acid) to yield compounds **1**–**14**.

General Procedure for Ilamycin NJL15–NJL18

To a solution of the corresponding carboxylic acid (3.0 equiv.) in dichloromethane (2 mL) was added EDC (3.0 equiv.), 4-dimethylaminopyridine (DMAP, 0.2 equiv.) and ilamycin F (20 mg, 1.0 equiv.) at 0 °C. The reaction mixture was stirred at room temperature for 10 h and then diluted with dichloromethane, washed with brine and dried over anhydrous Na_2SO_4. The solvent was removed under reduced pressure and purified by semi-preparative HPLC (3:2 v/v MeCN-H_2O and 0.1% formic acid) to yield compounds **15**–**18**.

*Ilamcyin NJL1 (**1**)*. Yellow oil; 80% yield; $[\alpha]_D^{25}$ -67.2(*c* 0.1, MeOH); ^{1}H NMR (700 MHz, CD_3OD) δ 7.74 (s, 1H), 7.56 – 7.51 (m, 2H), 7.42 (dd, *J* = 8.4, 1.7 Hz, 1H), 7.13 (s, 1H), 7.10 – 7.04 (m, 3H), 6.14 (dd, *J* = 17.4, 10.7 Hz, 1H), 5.54 – 5.47 (m, 1H), 5.24 – 5.13 (m, 3H), 4.82 – 4.81 (m, 1H), 4.76 – 4.71 (m, 2H), 4.69 – 4.65 (m, 1H), 4.58 – 4.56 (m, 1H), 4.55 – 4.51 (m, 2H), 4.39 (d, *J* = 3.2 Hz, 1H), 4.31 – 4.27 (m, 1H), 3.63 (s, 3H), 3.43 – 3.39 (m, 1H), 3.18 – 3.15 (m, 1H), 3.01 (dd, *J* = 12.5, 10.7 Hz, 1H), 2.87 – 2.82 (m, 1H), 2.78 – 2.75 (m, 1H), 2.71 (s, 3H), 2.69 – 2.66 (m, 4H), 2.58 – 2.53 (m, 1H), 2.32 – 2.28 (m, 1H), 1.76 – 1.68 (m, 10H), 1.64 – 1.61 (m, 1H), 1.57 (d, *J* = 6.0 Hz, 3H), 1.21 (d, *J* = 6.9 Hz, 3H), 1.17 (d, *J* = 6.3 Hz, 3H), 1.14 (d, *J* = 6.8 Hz, 3H), 1.10 – 1.06 (m, 1H), 1.01 – 0.94 (m, 6H), 0.38 (d, *J* = 6.7 Hz, 3H), 0.11 (d, *J* = 6.6 Hz, 3H); ^{1}H NMR (700 MHz, CD_3OD) δ 7.74 (s, 1H), 7.56 – 7.51 (m, 2H), 7.42 (dd, *J* = 8.4, 1.7 Hz, 1H), 7.13 (s, 1H), 7.10 – 7.04 (m, 3H), 6.14 (dd, *J* = 17.4, 10.7 Hz, 1H), 5.54 – 5.47 (m, 1H), 5.24 – 5.13 (m, 3H), 4.82 – 4.81 (m, 1H), 4.76 – 4.71 (m, 2H), 4.69 – 4.65 (m, 1H), 4.58 – 4.56 (m, 1H), 4.55 – 4.51 (m, 2H), 4.39 (d, *J* = 3.2 Hz, 1H), 4.31 – 4.27 (m, 1H), 3.63 (s, 3H), 3.43 – 3.39 (m, 1H), 3.18 – 3.15 (m, 1H), 3.01 (dd, *J* = 12.5, 10.7 Hz, 1H), 2.87 – 2.82 (m, 1H), 2.78 – 2.75 (m, 1H), 2.71 (s, 3H), 2.69 – 2.66 (m, 4H), 2.58 – 2.53 (m, 1H), 2.32

– 2.28 (m, 1H), 1.76 – 1.68 (m, 10H), 1.64 – 1.61 (m, 1H), 1.57 (d, *J* = 6.0 Hz, 3H), 1.21 (d, *J* = 6.9 Hz, 3H), 1.17 (d, *J* = 6.3 Hz, 3H), 1.14 (d, *J* = 6.8 Hz, 3H), 1.10 – 1.06 (m, 1H), 1.01 – 0.94 (m, 6H), 0.38 (d, *J* = 6.7 Hz, 3H), 0.11 (d, *J* = 6.6 Hz, 3H); HRMS (ESI): m/z $[M+H]^+$ calcd for $C_{59}H_{85}N_{10}O_{14}$: 1157.6247; found: 1157.6243.

*Ilamcyin NJL2 (**2**).* Yellow solid; 76% yield; $[\alpha]_D^{25}$ -84.8(*c* 0.1, MeOH); ^1H NMR (700 MHz, CD_3OD) δ 7.75 (d, *J* = 2.1 Hz, 1H), 7.56 – 7.55 (m, 1H), 7.53 (d, *J* = 8.2 Hz, 1H), 7.44 (dd, *J* = 8.6, 2.2 Hz, 1H), 7.13 (s, 1H), 7.11 – 7.05 (m, 3H), 6.14 (dd, *J* = 17.5, 10.7 Hz, 1H), 5.54 – 5.48 (m, 1H), 5.25 – 5.13 (m, 3H), 4.84 (dd, *J* = 11.3, 4.4 Hz, 1H), 4.78 – 4.74 (m, 2H), 4.69 (dd, *J* = 10.9, 2.5 Hz, 1H), 4.60 (t, *J* = 4.2 Hz, 1H), 4.58 – 4.53 (m, 2H), 4.46 (dd, *J* = 9.1, 4.7 Hz, 1H), 3.59 (s, 3H), 3.41 (dd, *J* = 13.1, 11.4 Hz, 1H), 3.18 (dd, *J* = 13.2, 4.2 Hz, 1H), 3.00 (dd, *J* = 13.0, 10.5 Hz, 1H), 2.88 – 2.83 (m, 1H), 2.71 (s, 3H), 2.70 – 2.69 (m, 1H), 2.68 (s, 3H), 2.61 – 2.55 (m, 2H), 2.55 – 2.45 (m, 2H), 2.32 – 2.26 (m, 1H), 2.11 (s, 3H), 2.10 – 2.05 (m, 2H), 2.00 – 1.94 (m, 1H), 1.77 – 1.70 (m, 9H), 1.68 – 1.60 (m, 3H), 1.56 (d, *J* = 6.3 Hz, 3H), 1.18 – 1.13 (m, 6H), 1.11 – 1.04 (m, 1H), 0.99 (d, *J* = 5.8 Hz, 3H), 0.96 (d, *J* = 5.9 Hz, 3H), 0.38 (d, *J* = 6.7 Hz, 3H), 0.12 (d, *J* = 6.7 Hz, 3H); ^{13}C NMR (176 MHz, CD_3OD) δ 179.06, 174.42, 173.92, 173.84, 173.82, 172.85, 172.24, 170.65, 169.44, 154.70, 145.42, 139.12, 136.96, 135.56, 131.47, 130.43, 129.56, 126.73, 125.49, 125.08, 122.23, 121.18, 120.42, 119.43, 115.40, 114.14, 108.89, 60.23, 59.84, 59.28, 55.80, 54.94, 53.39, 52.90, 52.79, 51.97, 46.77, 45.77, 39.00, 38.48, 38.46, 36.15, 35.54, 31.22, 31.09, 29.80, 29.69, 28.54, 28.43, 28.18, 25.94, 25.51, 23.51, 23.14, 22.22, 21.11, 19.40, 18.71, 16.66, 15.31; HRMS (ESI): m/z $[M+H]^+$ calcd for $C_{60}H_{87}N_{10}O_{13}S_1$: 1187.6175; found: 1187.6191.

*Ilamcyin NJL3 (**3**).* Yellow solid; 78% yield; $[\alpha]_D^{25}$ -78.4(*c* 0.1, MeOH); ^1H NMR (700 MHz, CD_3OD) δ 7.75 (d, *J* = 2.1 Hz, 1H), 7.54 (d, *J* = 8.4 Hz, 1H), 7.52 (d, *J* = 7.9 Hz, 1H), 7.43 (dd, *J* = 8.6, 2.2 Hz, 1H), 7.13 (s, 1H), 7.11 – 7.05 (m, 3H), 6.14 (dd, *J* = 17.5, 10.7 Hz, 1H), 5.53 – 5.47 (m, 1H), 5.24 – 5.14 (m, 3H), 4.83 (dd, *J* = 11.3, 4.4 Hz, 1H), 4.80 – 4.76 (m, 1H), 4.76 – 4.73 (m, 1H), 4.69 (dd, *J* = 11.0, 2.6 Hz, 1H), 4.57 – 4.52 (m, 3H), 4.23 (d, *J* = 5.5 Hz, 1H), 3.57 (s, 3H), 3.41 (dd, *J* = 13.2, 11.3 Hz, 1H), 3.16 (dd, *J* = 13.2, 4.1 Hz, 1H), 3.00 (dd, *J* = 13.0, 10.5 Hz, 1H), 2.89 – 2.84 (m, 1H), 2.76 – 2.72 (m, 1H), 2.71 (s, 3H), 2.70 – 2.68 (m, 4H), 2.60 – 2.55 (m, 1H), 2.32 – 2.27 (m, 1H), 1.89 – 1.84 (m, 1H), 1.75 – 1.70 (m, 9H), 1.67 – 1.60 (m, 3H), 1.56 (d, *J* = 6.4 Hz, 3H), 1.20 – 1.13 (m, 9H), 1.11 – 1.05 (m, 1H), 0.99 (d, *J* = 5.9 Hz, 3H), 0.97 – 0.94 (m, 6H), 0.91 (d, *J* = 6.8 Hz, 3H), 0.39 (d, *J* = 6.7 Hz, 3H), 0.12 (d, *J* = 6.7 Hz, 3H); ^{13}C NMR (176 MHz, CD_3OD) δ 179.01, 174.56, 173.94, 173.91, 173.29, 172.77, 172.21, 170.68, 169.48, 154.90, 145.44, 139.13, 137.01, 135.60, 131.48, 130.44,

129.51, 126.76, 125.52, 125.09, 122.27, 121.29, 120.40, 119.32, 115.45, 114.17, 108.87, 60.25, 59.89, 59.34, 58.58, 55.83, 54.91, 53.32, 52.37, 51.97, 46.73, 45.67, 38.67, 38.54, 38.46, 37.64, 36.15, 35.56, 29.83, 29.72, 28.54, 28.43, 28.21, 26.62, 25.95, 25.52, 23.53, 23.16, 22.19, 21.23, 19.39, 18.71, 16.75, 16.08, 12.01; HRMS (ESI): m/z $[M+H]^+$ calcd for $C_{61}H_{89}N_{10}O_{13}$: 1169.6611; found: 1169.6594.

*Ilamcyin NJL4 (**4**).* Yellow solid; 83% yield; $[\alpha]_D^{25}$ -79.6(*c* 0.1, $CHCl_3$); ^{1}H NMR (700 MHz, CD_3OD) δ 7.74 – 7.73 (m, 1H), 7.55 – 7.52 (m, 2H), 7.43 (dd, *J* = 8.7, 2.1 Hz, 1H), 7.12 (s, 1H), 7.10 – 7.04 (m, 3H), 6.13 (dd, *J* = 17.5, 10.7 Hz, 1H), 5.55 – 5.49 (m, 1H), 5.24 – 5.14 (m, 3H), 4.82 (dd, *J* = 11.3, 4.3 Hz, 1H), 4.74 (t, *J* = 7.6 Hz, 1H), 4.70 (t, *J* = 6.9 Hz, 1H), 4.66 (dd, *J* = 10.3, 2.9 Hz, 1H), 4.57 (t, *J* = 4.2 Hz, 1H), 4.55 – 4.52 (m, 2H), 4.42 (dd, *J* = 9.6, 4.7 Hz, 1H), 3.60 (s, 3H), 3.44 – 3.38 (m, 1H), 3.22 – 3.17 (m, 3H), 3.00 (dd, *J* = 13.0, 10.4 Hz, 1H), 2.86 – 2.80 (m, 1H), 2.71 – 2.70 (m, 3H), 2.69 – 2.65 (m, 5H), 2.61 – 2.55 (m, 1H), 2.31 – 2.25 (m, 1H), 1.75 – 1.70 (m, 12H), 1.67 – 1.60 (m, 4H), 1.57 (d, *J* = 6.3 Hz, 3H), 1.21 – 1.15 (m, 6H), 1.11 – 1.05 (m, 1H), 0.99 (d, *J* = 5.8 Hz, 3H), 0.96 (d, *J* = 5.8 Hz, 3H), 0.37 (d, *J* = 6.7 Hz, 3H), 0.11 (d, *J* = 6.6 Hz, 3H); ^{13}C NMR (176 MHz, CD_3OD) δ 179.00, 174.28, 173.86, 173.69, 172.92, 172.35, 170.76, 169.40, 158.69, 154.89, 145.39, 139.08, 136.95, 135.61, 131.54, 130.41, 129.35, 126.76, 125.50, 125.03, 122.19, 121.27, 120.36, 119.32, 115.42, 114.13, 108.80, 60.22, 59.81, 59.25, 55.71, 54.97, 53.36, 53.07, 52.85, 51.89, 46.80, 45.60, 41.79, 39.13, 38.37, 36.22, 35.37, 29.76, 29.70, 29.13, 28.51, 28.39, 28.17, 26.40, 25.92, 25.45, 23.47, 23.08, 22.18, 20.98, 19.53, 18.67, 16.50; HRMS (ESI): m/z $[M+H]^+$ calcd for $C_{61}H_{90}N_{13}O_{13}$: 1212.6781; found: 1212.6799.

*Ilamcyin NJL5 (**5**).* Yellow solid; 83% yield; $[\alpha]_D^{25}$ -81.4(*c* 0.1, $CHCl_3$); ^{1}H NMR (700 MHz, CD_3OD) δ 7.74 (d, *J* = 2.1 Hz, 1H), 7.72 (s, 1H), 7.54 (d, *J* = 7.6 Hz, 1H), 7.52 (d, *J* = 8.3 Hz, 1H), 7.43 (dd, *J* = 8.7, 2.2 Hz, 1H), 7.12 (s, 1H), 7.10 – 7.04 (m, 3H), 6.89 (s, 1H), 6.13 (dd, *J* = 17.5, 10.7 Hz, 1H), 5.54 – 5.48 (m, 1H), 5.23 – 5.14 (m, 3H), 4.83 (dd, *J* = 11.3, 4.3 Hz, 1H), 4.73 (t, *J* = 7.5 Hz, 1H), 4.71 – 4.68 (m, 1H), 4.67 – 4.63 (m, 2H), 4.57 (t, *J* = 4.2 Hz, 1H), 4.54 – 4.53 (m, 1H), 4.53 – 4.51 (m, 1H), 3.59 (s, 3H), 3.40 (dd, *J* = 13.1, 11.4 Hz, 1H), 3.20 – 3.16 (m, 1H), 3.13 (dd, *J* = 15.0, 4.6 Hz, 1H), 2.99 (dd, *J* = 13.0, 10.3 Hz, 1H), 2.95 – 2.91 (m, 1H), 2.86 – 2.81 (m, 1H), 2.70 (d, *J* = 4.6 Hz, 1H), 2.68 (s, 3H), 2.66 (s, 3H), 2.61 – 2.55 (m, 2H), 2.26 – 2.21 (m, 1H), 1.74 – 1.70 (m, 9H), 1.67 – 1.59 (m, 3H), 1.56 (d, *J* = 6.4 Hz, 3H), 1.15 (d, *J* = 6.9 Hz, 3H), 1.09 – 1.06 (m, 1H), 1.05 (d, *J* = 6.9 Hz, 3H), 0.98 (d, *J* = 5.9 Hz, 3H), 0.95 (d, *J* = 5.8 Hz, 3H), 0.37 (d, *J* = 6.7 Hz, 3H), 0.10 (d, *J* = 6.7 Hz, 3H); ^{13}C NMR (176 MHz, CD_3OD) δ 178.58, 174.33, 173.89, 173.85, 173.33, 172.90, 172.31, 170.72,

169.43, 154.66, 145.40, 139.11, 136.94, 136.31, 135.53, 134.52, 131.45, 130.42, 129.56, 126.73, 125.46, 125.09, 122.21, 121.14, 120.40, 119.41, 117.83, 115.36, 114.11, 108.85, 60.20, 59.83, 59.27, 55.70, 54.96, 53.70, 53.37, 52.82, 51.90, 46.82, 45.54, 39.10, 38.38, 36.16, 35.36, 29.76, 29.68, 29.50, 28.50, 28.39, 28.18, 25.92, 25.46, 23.47, 23.09, 22.17, 21.02, 19.24, 18.67, 16.54; HRMS (ESI): m/z $[M+H]^+$ calcd for $C_{61}H_{85}N_{12}O_{13}$: 1193.6359; found: 1193.6359.

*Ilamcyin NJL6 (**6**)*. Yellow solid; 82% yield; $[\alpha]_D^{25}$ -71.8(*c* 0.1, MeOH); ^1H NMR (700 MHz, CD_3OD) δ 7.74 (d, *J* = 2.1 Hz, 1H), 7.54 – 7.51 (m, 2H), 7.49 (d, *J* = 7.8 Hz, 1H), 7.43 (dd, *J* = 8.7, 2.2 Hz, 1H), 7.35 (d, *J* = 8.1 Hz, 1H), 7.13 – 7.09 (m, 2H), 7.08 – 7.02 (m, 5H), 6.12 (dd, *J* = 17.5, 10.7 Hz, 1H), 5.55 – 5.49 (m, 1H), 5.23 – 5.16 (m, 3H), 4.80 (dd, *J* = 11.2, 4.4 Hz, 1H), 4.74 (t, *J* = 7.5 Hz, 1H), 4.71 – 4.68 (m, 1H), 4.68 – 4.65 (m, 1H), 4.64 (dd, *J* = 9.2, 5.2 Hz, 1H), 4.58 (t, *J* = 4.3 Hz, 1H), 4.55 (dd, *J* = 10.1, 4.7 Hz, 1H), 4.51 (dd, *J* = 11.9, 3.0 Hz, 1H), 3.55 (s, 3H), 3.44 – 3.35 (m, 2H), 3.17 – 3.14 (m, 1H), 3.13 – 3.08 (m, 1H), 3.00 (dd, *J* = 13.0, 10.2 Hz, 1H), 2.87 – 2.83 (m, 1H), 2.70 (d, *J* = 4.7 Hz, 1H), 2.67 (s, 3H), 2.64 (s, 3H), 2.61 – 2.56 (m, 2H), 2.25 – 2.21 (m, 1H), 1.75 – 1.70 (m, 9H), 1.63 – 1.59 (m, 2H), 1.57 (d, *J* = 6.4 Hz, 3H), 1.14 (d, *J* = 6.9 Hz, 3H), 1.08 – 1.04 (m, 1H), 1.01 – 0.94 (m, 9H), 0.37 (d, *J* = 6.7 Hz, 3H), 0.09 (d, *J* = 6.7 Hz, 3H); ^{13}C NMR (176 MHz, CD_3OD) δ 178.54, 174.40, 173.96, 173.92, 173.88, 172.86, 172.34, 170.73, 169.44, 154.66, 145.41, 139.11, 138.07, 136.91, 135.53, 131.41, 130.41, 129.59, 128.64, 126.73, 125.43, 125.16, 124.37, 122.52, 122.19, 121.14, 120.42, 119.86, 119.49, 119.12, 115.32, 114.10, 112.38, 110.98, 108.85, 60.19, 59.82, 59.31, 55.64, 54.94, 54.83, 53.43, 52.70, 51.93, 46.83, 45.45, 38.98, 38.39, 38.36, 36.12, 35.41, 29.74, 29.69, 28.49, 28.40, 28.21, 28.00, 25.94, 25.46, 23.48, 23.09, 22.19, 21.03, 19.11, 18.65, 16.58; HRMS (ESI): m/z $[M+H]^+$ calcd for $C_{66}H_{87}N_{11}O_{13}$: 1242.6563; found: 1242.6557.

*Ilamcyin NJL7 (**7**)*. Yellow solid; 76% yield; $[\alpha]_D^{25}$ -58.3(*c* 0.1, MeOH); ^1H NMR (700 MHz, CD_3OD) δ 7.74 (d, *J* = 2.2 Hz, 1H), 7.54 (t, *J* = 7.6 Hz, 2H), 7.44 (dd, *J* = 8.7, 2.2 Hz, 1H), 7.30 – 7.19 (m, 5H), 7.13 (s, 1H), 7.11 – 7.08 (m, 1H), 7.08 – 7.06 (m, 2H), 6.14 (dd, *J* = 17.5, 10.7 Hz, 1H), 5.55 – 5.49 (m, 1H), 5.25 – 5.15 (m, 3H), 4.83 (dd, *J* = 11.3, 4.4 Hz, 1H), 4.75 (t, *J* = 7.5 Hz, 1H), 4.70 (q, *J* = 7.0 Hz, 1H), 4.62 (dd, *J* = 10.7, 2.6 Hz, 1H), 4.60 – 4.58 (m, 2H), 4.56 – 4.53 (m, 2H), 3.56 (s, 3H), 3.42 (dd, *J* = 13.2, 11.4 Hz, 1H), 3.19 (dd, *J* = 13.2, 4.1 Hz, 1H), 3.16 (dd, *J* = 13.9, 5.4 Hz, 1H), 3.00 (dd, *J* = 13.0, 10.4 Hz, 1H), 2.92 (dd, *J* = 9.4, 4.5 Hz, 1H), 2.88 – 2.83 (m, 1H), 2.75 – 2.73 (m, 1H), 2.71 (d, *J* = 4.5 Hz, 1H), 2.68 (s, 5H), 2.62 – 2.55 (m, 2H), 2.26 – 2.21 (m, 1H), 1.77 – 1.70 (m, 8H), 1.66 – 1.59 (m, 3H), 1.57 (d, *J* = 6.4 Hz, 3H), 1.14 (d, *J* = 6.9 Hz, 3H), 1.10 – 1.05 (m, 1H), 0.97 (tt, *J* = 9.5,

4.7 Hz, 8H), 0.38 (d, *J* = 6.7 Hz, 3H), 0.11 (d, *J* = 6.7 Hz, 3H); ^{13}C NMR (176 MHz, CD_3OD) δ 178.57, 174.33, 173.93, 173.88, 173.60, 172.89, 172.35, 170.69, 169.46, 154.74, 145.44, 139.13, 138.41, 136.98, 135.58, 131.51, 130.21, 129.53, 127.91, 126.76, 125.50, 125.10, 122.26, 121.20, 120.44, 119.44, 115.42, 114.16, 108.88, 60.25, 59.87, 59.33, 55.76, 55.26, 54.98, 53.39, 52.72, 51.98, 46.83, 45.68, 39.07, 38.44, 37.95, 36.24, 35.38, 29.81, 29.70, 28.55, 28.43, 28.23, 25.96, 25.51, 23.52, 23.15, 22.20, 21.10, 19.24, 18.71, 16.59; HRMS (ESI): m/z $[M+H]^+$ calcd for $C_{64}H_{87}N_{10}O_{13}$: 1203.6454; found: 1203.6452.

*Ilamcyin NJL8 (**8**).* Yellow solid; 79% yield; $[\alpha]_D^{25}$ -58.0(*c* 0.1, MeOH); ^{1}H NMR (700 MHz, CD_3OD) δ 7.74 (d, *J* = 2.1 Hz, 1H), 7.57 (d, *J* = 7.4 Hz, 1H), 7.53 (d, *J* = 8.3 Hz, 1H), 7.43 (dd, *J* = 8.7, 2.2 Hz, 1H), 7.14 (s, 1H), 7.11 – 7.05 (m, 3H), 7.02 – 6.99 (m, 2H), 6.74 – 6.70 (m, 2H), 6.14 (dd, *J* = 17.5, 10.7 Hz, 1H), 5.54 – 5.49 (m, 1H), 5.25 – 5.15 (m, 3H), 4.83 (dd, *J* = 11.3, 4.4 Hz, 1H), 4.75 (t, *J* = 7.5 Hz, 1H), 4.70 (q, *J* = 6.9 Hz, 1H), 4.63 (dd, *J* = 10.8, 2.7 Hz, 1H), 4.59 (t, *J* = 4.2 Hz, 1H), 4.56 – 4.53 (m, 2H), 4.50 (dd, *J* = 9.7, 5.7 Hz, 1H), 3.55 (s, 3H), 3.42 (dd, *J* = 13.2, 11.4 Hz, 1H), 3.21 (dd, *J* = 13.2, 4.2 Hz, 1H), 3.03 (dd, *J* = 14.1, 5.7 Hz, 1H), 3.01 – 2.98 (m, 1H), 2.89 – 2.84 (m, 1H), 2.81 (dd, *J* = 14.0, 9.7 Hz, 1H), 2.76 – 2.73 (m, 1H), 2.70 (d, *J* = 4.7 Hz, 1H), 2.69 (s, 3H), 2.67 (s, 3H), 2.62 – 2.56 (m, 2H), 2.27 – 2.22 (m, 1H), 1.77 – 1.70 (m, 10H), 1.65 – 1.59 (m, 3H), 1.57 (d, *J* = 6.4 Hz, 3H), 1.14 (d, *J* = 6.9 Hz, 3H), 1.10 – 1.06 (m, 1H), 1.01 (d, *J* = 6.9 Hz, 3H), 0.99 (d, *J* = 5.9 Hz, 3H), 0.96 (d, *J* = 5.9 Hz, 3H), 0.38 (d, *J* = 6.7 Hz, 3H), 0.12 (d, *J* = 6.7 Hz, 3H); ^{13}C NMR (176 MHz, CD_3OD) δ 178.55, 174.37, 173.97, 173.90, 173.78, 172.87, 172.34, 170.72, 169.48, 157.51, 154.78, 145.47, 139.15, 136.98, 135.58, 131.50, 131.19, 130.48, 129.57, 128.91, 126.76, 125.51, 125.12, 122.26, 121.22, 120.47, 119.55, 116.27, 115.38, 114.15, 108.92, 60.24, 59.87, 59.34, 55.75, 55.63, 54.99, 53.40, 52.65, 52.02, 46.83, 45.66, 39.05, 38.43, 37.26, 36.20, 35.40, 29.82, 29.71, 28.54, 28.45, 28.25, 25.95, 25.50, 23.53, 23.16, 22.20, 21.10, 19.25, 18.71, 16.60; HRMS (ESI): m/z $[M+H]^+$ calcd for $C_{64}H_{87}N_{10}O_{14}$: 1219.6403; found: 1219.6408.

*Ilamcyin NJL9 (**9**).* Yellow solid; 76% yield; $[\alpha]_D^{25}$ -82.5(*c* 0.1, $CHCl_3$); ^{1}H NMR (700 MHz, CD_3OD) δ 7.73 (d, *J* = 1.9 Hz, 1H), 7.49 (d, *J* = 8.5 Hz, 1H), 7.42 (dd, *J* = 8.6, 2.0 Hz, 1H), 7.35 – 7.31 (m, 3H), 7.27 – 7.23 (m, 2H), 7.19 (t, *J* = 7.4 Hz, 1H), 7.08 (s, 1H), 7.07 (d, *J* = 8.7 Hz, 1H), 7.06 – 7.02 (m, 1H), 6.93 (t, *J* = 7.4 Hz, 1H), 6.11 (dd, *J* = 17.5, 10.7 Hz, 1H), 5.57 – 5.51 (m, 1H), 5.22 – 5.15 (m, 3H), 4.82 (dd, *J* = 11.3, 4.3 Hz, 1H), 4.76 – 4.73 (m, 1H), 4.68 – 4.65 (m, 2H), 4.61 – 4.56 (m, 3H), 4.26 (s, 2H), 3.46 – 3.44 (m, 1H), 3.14 (dd, *J* = 13.2, 4.2 Hz, 1H), 3.02 – 2.96 (m, 1H), 2.90 – 2.84 (m, 1H), 2.77 – 2.75 (m, 1H), 2.72 (dd, *J* = 13.1, 4.8 Hz, 1H), 2.68 (s, 3H), 2.66 (s, 3H), 2.64 – 2.58 (m,

2H), 2.29 – 2.23 (m, 1H), 1.73 – 1.69 (m, 9H), 1.64 – 1.60 (m, 3H), 1.58 (d, *J* = 6.4 Hz, 3H), 1.17 (d, *J* = 6.9 Hz, 3H), 1.11 – 1.07 (m, 1H), 1.01 – 0.96 (m, 9H), 0.35 (d, *J* = 6.7 Hz, 3H), 0.08 (d, *J* = 6.6 Hz, 3H); ^{13}C NMR (176 MHz, CD_3OD) δ 178.24, 174.42, 173.97, 173.87, 173.09, 172.45, 170.46, 169.54, 154.78, 145.42, 140.61, 139.09, 136.90, 135.59, 131.60, 130.41, 129.72, 128.73, 128.28, 126.72, 125.43, 125.15, 122.17, 121.27, 120.39, 119.52, 115.30, 114.14, 108.79, 60.22, 59.89, 59.51, 55.51, 54.80, 53.42, 52.16, 46.97, 45.60, 44.37, 39.21, 38.75, 38.50, 36.29, 35.37, 29.83, 29.78, 28.52, 28.42, 28.23, 26.00, 25.47, 23.56, 23.14, 22.21, 21.18, 19.36, 18.68, 16.61; HRMS (ESI): m/z $[M+H]^+$ calcd for $C_{61}H_{83}N_{10}O_{11}$: 1131.6243; found: 1131.6239.

*Ilamcyin NJL10 (**10**)*. Yellow solid; 77% yield; $[\alpha]_D^{25}$ -89.0(*c* 0.1, $CHCl_3$); ^{1}H NMR (700 MHz, CD_3OD) δ 7.72 (d, *J* = 2.0 Hz, 1H), 7.49 (d, *J* = 8.4 Hz, 1H), 7.43 (dd, *J* = 8.6, 2.1 Hz, 1H), 7.39 (d, *J* = 8.0 Hz, 1H), 7.24 (dd, *J* = 8.4, 5.5 Hz, 2H), 7.08 (s, 1H), 7.08 – 7.06 (m, 2H), 7.06 – 7.02 (m, 2H), 6.94 (t, *J* = 7.5 Hz, 1H), 6.14 – 6.08 (m, 1H), 5.56 – 5.52 (m, 1H), 5.22 – 5.14 (m, 3H), 4.88 – 4.84 (m, 1H), 4.75 (t, *J* = 7.5 Hz, 1H), 4.68 – 4.63 (m, 2H), 4.62 – 4.54 (m, 3H), 4.30 (d, *J* = 14.9 Hz, 1H), 4.18 (d, *J* = 14.7 Hz, 1H), 3.39 (dd, *J* = 13.1, 11.2 Hz, 1H), 3.16 (dd, *J* = 13.4, 4.4 Hz, 1H), 3.00 – 2.95 (m, 1H), 2.89 – 2.84 (m, 1H), 2.74 (d, *J* = 2.7 Hz, 1H), 2.72 (dd, *J* = 13.1, 4.9 Hz, 1H), 2.68 (s, 3H), 2.65 (s, 3H), 2.64 – 2.57 (m, 2H), 2.29 – 2.24 (m, 1H), 1.72 – 1.68 (m, 9H), 1.66 – 1.60 (m, 3H), 1.58 (d, *J* = 6.6 Hz, 3H), 1.17 (d, *J* = 6.9 Hz, 3H), 1.10 – 1.07 (m, 1H), 1.02 – 0.96 (m, 9H), 0.35 (d, *J* = 6.6 Hz, 3H), 0.07 (d, *J* = 6.6 Hz, 3H); ^{13}C NMR (176 MHz, CD_3OD) δ 178.23, 174.33, 174.03, 173.79, 173.10, 172.55, 170.40, 169.63, 164.61, 154.71, 145.42, 139.12, 136.89, 136.67, 135.57, 131.54, 130.55, 130.50, 130.46, 129.51, 126.73, 125.40, 125.25, 122.15, 121.22, 120.31, 119.54, 116.44, 116.31, 115.25, 114.12, 108.83, 60.20, 59.83, 59.42, 55.48, 54.83, 53.45, 52.13, 46.93, 45.52, 43.59, 39.13, 38.83, 38.50, 36.26, 35.33, 29.82, 29.75, 28.49, 28.41, 28.22, 25.99, 25.35, 23.52, 23.11, 22.26, 21.12, 19.38, 18.66, 16.61; HRMS (ESI): m/z $[M+H]^+$ calcd for $C_{61}H_{82}F_1N_{10}O_{11}$: 1149.6149; found: 1149.6162.

*Ilamcyin NJL11 (**11**)*. Yellow solid; 74% yield; $[\alpha]_D^{25}$ -88.2(*c* 0.1, $CHCl_3$); ^{1}H NMR (700 MHz, CD_3OD) δ 7.74 (d, *J* = 1.8 Hz, 1H), 7.49 (d, *J* = 8.5 Hz, 1H), 7.42 (dd, *J* = 8.6, 1.9 Hz, 1H), 7.37 – 7.34 (m, 3H), 7.25 – 7.20 (m, 2H), 7.08 (s, 1H), 7.08 – 7.03 (m, 2H), 6.94 (t, *J* = 7.4 Hz, 1H), 6.11 (dd, *J* = 17.5, 10.7 Hz, 1H), 5.54 (mf, 1H), 5.24 – 5.14 (m, 3H), 4.84 – 4.83 (m, 1H), 4.76 – 4.70 (m, 2H), 4.67 – 4.61 (m, 3H), 4.47 (q, *J* = 6.7 Hz, 1H), 4.27 (d, *J* = 14.7 Hz, 1H), 4.20 (d, *J* = 14.7 Hz, 1H), 3.40 (dd, *J* = 13.1, 11.2 Hz, 1H), 3.16 (dd, *J* = 13.3, 4.4 Hz, 1H), 2.99 – 2.95 (m, 1H), 2.91 – 2.84 (m, 1H), 2.75 – 2.71

(m, 1H), 2.70 (s, 3H), 2.64 (s, 3H), 2.63 – 2.60 (m, 1H), 2.60 – 2.56 (m, 1H), 2.30 – 2.23 (m, 1H), 1.75 – 1.67 (m, 10H), 1.66 – 1.62 (m, 1H), 1.58 (d, *J* = 6.2 Hz, 3H), 1.17 (d, *J* = 6.9 Hz, 3H), 1.08 (d, *J* = 6.8 Hz, 3H), 1.06 (d, *J* = 6.9 Hz, 1H), 1.01 – 0.95 (m, 6H), 0.34 (d, *J* = 6.6 Hz, 3H), 0.10 (d, *J* = 6.6 Hz, 3H); ^{13}C NMR (176 MHz, CD_3OD) δ 178.24, 174.50, 173.96, 173.83, 173.17, 172.62, 170.33, 169.53, 154.63, 145.37, 139.04, 136.85, 135.60, 134.05, 131.52, 130.53, 130.39, 130.16, 129.76, 129.62, 126.69, 125.36, 125.21, 122.10, 121.30, 120.29, 119.49, 115.24, 114.08, 108.80, 60.17, 59.79, 59.52, 55.45, 54.70, 53.48, 52.07, 46.91, 45.45, 43.76, 38.98, 38.85, 38.39, 36.17, 35.14, 29.79, 29.76, 28.45, 28.38, 28.21, 25.97, 25.38, 23.45, 23.12, 22.17, 21.19, 19.27, 18.63, 16.76; HRMS (ESI): m/z $[M+H]^+$ calcd for $C_{61}H_{82}Cl_1N_{10}O_{11}$: 1165.5853; found: 1165.5855.

*Ilamcyin NJL12 (**12**)*. Yellow solid; 78% yield; $[\alpha]_D^{25}$ -90.9(*c* 0.1, $CHCl_3$); ^{1}H NMR (700 MHz, CD_3OD) δ 7.75 (d, *J* = 2.1 Hz, 1H), 7.66 (d, *J* = 8.2 Hz, 2H), 7.50 (d, *J* = 8.5 Hz, 1H), 7.43 (d, *J* = 8.4 Hz, 2H), 7.42 (d, *J* = 2.2 Hz, 1H), 7.33 (d, *J* = 8.0 Hz, 1H), 7.09 (s, 1H), 7.08 (d, *J* = 8.6 Hz, 1H), 7.06 – 7.03 (m, 1H), 6.92 (t, *J* = 7.4 Hz, 1H), 6.12 (dd, *J* = 17.5, 10.7 Hz, 1H), 5.56 – 5.51 (m, 1H), 5.22 – 5.14 (m, 3H), 4.85 – 4.83 (m, 1H), 4.71 (t, *J* = 7.5 Hz, 1H), 4.66 – 4.58 (m, 5H), 4.50 (d, *J* = 15.0 Hz, 1H), 4.21 (d, *J* = 15.0 Hz, 1H), 3.41 (dd, *J* = 13.1, 11.4 Hz, 1H), 3.13 (dd, *J* = 13.3, 4.3 Hz, 1H), 3.00 – 2.96 (m, 1H), 2.89 – 2.83 (m, 1H), 2.74 (d, *J* = 4.9 Hz, 1H), 2.72 (s, 3H), 2.65 (s, 3H), 2.62 – 2.56 (m, 2H), 2.29 – 2.24 (m, 1H), 1.88 – 1.77 (m, 1H), 1.73 – 1.68 (m, 9H), 1.68 – 1.59 (m, 2H), 1.57 (d, *J* = 6.5 Hz, 3H), 1.19 (d, *J* = 6.9 Hz, 3H), 1.16 (d, *J* = 6.8 Hz, 3H), 1.09 – 1.06 (m, 1H), 0.99 (d, *J* = 6.1 Hz, 3H), 0.97 (d, *J* = 6.2 Hz, 3H), 0.34 (d, *J* = 6.7 Hz, 3H), 0.11 (d, *J* = 6.7 Hz, 3H); ^{13}C NMR (176 MHz, CD_3OD) δ 178.39, 174.71, 173.96, 173.92, 172.68, 170.53, 169.53, 154.72, 145.41, 144.75, 139.12, 136.90, 135.58, 131.55, 130.40, 129.51, 129.37, 128.89, 126.70, 126.61, 126.59, 125.45, 125.34, 125.23, 122.15, 121.24, 120.31, 119.40, 118.18, 115.32, 114.13, 108.80, 60.21, 59.78, 59.67, 55.47, 54.72, 53.49, 52.02, 46.92, 45.36, 43.86, 38.94, 38.72, 38.41, 36.22, 34.93, 29.92, 29.77, 28.50, 28.42, 28.25, 26.02, 25.44, 23.48, 23.20, 22.14, 21.05, 19.32, 18.67, 16.96; HRMS (ESI): m/z $[M+H]^+$ calcd for $C_{62}H_{83}F_3N_{10}O_{11}$: 1199.6117; found: 1199.6112.

*Ilamcyin NJL13 (**13**)*. Yellow solid; 76% yield; $[\alpha]_D^{25}$ -26.8(*c* 0.1, $CHCl_3$); ^{1}H NMR (700 MHz, CD_3OD) δ 8.69 (d, *J* = 5.7 Hz, 2H), 7.77 – 7.75 (m, 3H), 7.52 – 7.49 (m, 2H), 7.43 (dd, *J* = 8.6, 2.1 Hz, 1H), 7.11 (s, 1H), 7.08 – 7.04 (m, 2H), 7.04 – 7.00 (m, 1H), 6.12 (dd, *J* = 17.5, 10.7 Hz, 1H), 5.53 – 5.47 (m, 1H), 5.22 – 5.16 (m, 3H), 4.95 – 4.94 (m, 1H), 4.79 (dd, *J* = 11.2, 4.5 Hz, 1H), 4.72 (t, *J* = 7.4 Hz, 1H), 4.60 (d, *J* = 3.2 Hz, 1H), 4.58 (d, *J* = 4.5 Hz, 1H), 4.58 – 4.56 (m, 1H), 4.54 (t, *J* = 4.3 Hz, 1H),

3.37 (dd, J = 13.0, 11.5 Hz, 1H), 3.12 (d, J = 4.1 Hz, 1H), 3.02 (dd, J = 13.0, 10.2 Hz, 1H), 2.85 – 2.80 (m, 1H), 2.73 (s, 3H), 2.72 – 2.70 (m, 1H), 2.69 (d, J = 4.3 Hz, 1H), 2.65 (s, 3H), 2.57 – 2.52 (m, 1H), 2.35 – 2.30 (m, 1H), 1.84 – 1.79 (m, 1H), 1.74 – 1.70 (m, 9H), 1.66 – 1.58 (m, 2H), 1.57 (d, J = 6.2 Hz, 3H), 1.27 (d, J = 7.1 Hz, 3H), 1.16 (d, J = 6.9 Hz, 3H), 1.05 – 1.01 (m, 1H), 0.99 (d, J = 5.8 Hz, 3H), 0.96 (d, J = 5.9 Hz, 3H), 0.31 (d, J = 6.7 Hz, 3H), 0.04 (d, J = 6.6 Hz, 3H); ^{13}C NMR (176 MHz, CD_3OD) δ 179.27, 177.86, 174.84, 174.00, 173.90, 172.88, 172.26, 170.93, 169.59, 166.30, 154.86, 151.08, 145.42, 142.02, 139.13, 136.90, 135.65, 131.32, 130.46, 129.49, 126.81, 125.48, 125.27, 123.07, 122.17, 121.24, 120.37, 119.52, 115.31, 114.12, 108.88, 60.21, 59.76, 59.35, 55.64, 54.99, 53.43, 52.03, 46.95, 45.42, 38.43, 36.15, 35.13, 29.82, 28.51, 28.42, 28.20, 25.96, 25.42, 23.52, 23.18, 22.18, 21.11, 19.43, 18.66, 16.86; HRMS (ESI): m/z $[M+H]^+$ calcd for $C_{60}H_{81}N_{12}O_{12}$: 1161.6097; found: 1161.6107.

*Ilamcyin NJL14 (**14**).* Yellow solid; 77% yield; $[\alpha]_D^{25}$ -34.9(c 0.1, $CHCl_3$); ^{1}H NMR (700 MHz, CD_3OD) δ 7.68 (d, J = 2.0 Hz, 1H), 7.52 (d, J = 2.5 Hz, 1H), 7.33 (d, J = 7.9 Hz, 1H), 7.32 – 7.28 (m, 1H), 7.19 (dd, J = 8.9, 2.2 Hz, 1H), 7.07 (s, 1H), 7.06 – 7.02 (m, 2H), 6.99 – 6.95 (m, 2H), 6.10 (dd, J = 17.5, 10.7 Hz, 1H), 5.48 (dq, J = 13.1, 6.5 Hz, 1H), 5.20 – 5.13 (m, 3H), 4.81 – 4.77 (m, 2H), 4.70 – 4.67 (m, 2H), 4.67 – 4.63 (m, 1H), 4.61 (d, J = 3.0 Hz, 1H), 4.58 (t, J = 5.0 Hz, 2H), 4.03 (t, J = 9.0 Hz, 1H), 3.82 – 3.80 (m, 1H), 3.79 – 3.77 (m, 4H), 3.53 (dd, J = 14.4, 4.0 Hz, 1H), 3.44 (dd, J = 14.5, 5.5 Hz, 1H), 3.33 (t, J = 12.2 Hz, 1H), 3.04 (dd, J = 13.1, 4.2 Hz, 1H), 3.00 – 2.98 (m, 4H), 2.87 – 2.82 (m, 1H), 2.76 – 2.72 (m, 1H), 2.69 (s, 3H), 2.66 – 2.62 (m, 1H), 2.60 (s, 3H), 2.59 – 2.55 (m, 1H), 2.53 – 2.50 (m, 1H), 2.19 (s, 1H), 1.71 (s, 1H), 1.70 – 1.67 (m, 9H), 1.61 – 1.55 (m, 2H), 1.54 (d, J = 6.1 Hz, 3H), 1.25 (d, J = 6.8 Hz, 3H), 1.08 (d, J = 7.0 Hz, 3H), 1.06 – 1.03 (m, 1H), 0.96 (d, J = 6.0 Hz, 3H), 0.93 (d, J = 6.1 Hz, 3H), 0.34 (d, J = 6.7 Hz, 3H), 0.14 (d, J = 6.7 Hz, 3H); ^{13}C NMR (176 MHz, CD_3OD) δ 179.21, 174.59, 173.96, 173.78, 173.20, 172.66, 170.49, 169.49, 157.21, 156.20, 156.14, 145.39, 138.63, 136.85, 136.20, 135.01, 134.95, 131.38, 130.41, 127.55, 126.84, 125.52, 125.25, 122.50, 122.09, 120.42, 120.30, 119.56, 115.35, 115.25, 114.08, 108.81, 108.43, 73.24, 67.97, 60.17, 59.74, 59.69, 55.48, 54.75, 53.44, 52.30, 52.00, 47.40, 46.71, 45.52, 43.16, 38.88, 38.72, 38.37, 36.08, 34.64, 29.87, 29.77, 28.48, 28.41, 28.13, 25.93, 25.44, 23.50, 23.21, 22.08, 21.06, 19.57, 18.63, 17.10; HRMS (ESI): m/z $[M+H]^+$ calcd for $C_{68}H_{92}FN_{12}O_{14}$: 1319.6840; found: 1319.6847.

*Ilamcyin NJL15 (**15**).* Yellow solid; 40% yield; $[\alpha]_D^{25}$ -32.7(c 0.1, MeOH); ^{1}H NMR (700 MHz, CD_3OD) δ 7.82 (d, J = 2.1 Hz, 1H), 7.57 (d, J = 8.0 Hz, 1H), 7.54 (d, J = 7.7 Hz, 1H), 7.52 (d, J = 8.3

Hz, 1H), 7.47 (dd, J = 8.4, 2.0 Hz, 1H), 7.34 (d, J = 8.1 Hz, 1H), 7.11 (t, J = 6.4 Hz, 2H), 7.09 – 7.01 (m, 5H), 6.13 (dd, J = 17.5, 10.7 Hz, 1H), 5.51 (dt, J = 19.8, 5.5 Hz, 1H), 5.23 – 5.15 (m, 3H), 4.97 (dd, J = 8.6, 5.2 Hz, 1H), 4.89 – 4.89 (m, 1H), 4.80 (dd, J = 13.9, 7.0 Hz, 1H), 4.66 – 4.60 (m, 3H), 4.58 (t, J = 4.6 Hz, 1H), 3.39 (dd, J = 13.1, 11.3 Hz, 1H), 3.20 – 3.18 (m, 3H), 3.14 (dd, J = 13.2, 9.3 Hz, 1H), 3.00 (t, J = 7.6 Hz, 2H), 2.84 – 2.78 (m, 2H), 2.74 (d, J = 9.3 Hz, 3H), 2.59 (s, 3H), 2.55 – 2.49 (m, 1H), 2.49 – 2.42 (m, 1H), 2.21 – 2.15 (m, 1H), 1.89 – 1.84 (m, 1H), 1.73 – 1.65 (m, 9H), 1.63 – 1.59 (m, 1H), 1.58 (d, J = 6.5 Hz, 3H), 1.29 (d, J = 6.9 Hz, 3H), 1.23 (d, J = 7.1 Hz, 3H), 1.15 – 1.13 (m, 4H), 1.10 – 1.05 (m, 1H), 0.99 – 0.92 (m, 6H), 0.37 (d, J = 6.7 Hz, 3H), 0.16 (d, J = 6.7 Hz, 3H); ^{13}C NMR (176 MHz, CD_3OD) δ 180.51, 175.01, 174.02, 173.90, 173.19, 172.44, 172.32, 170.92, 169.68, 145.42, 144.20, 143.13, 138.19, 136.91, 136.83, 131.35, 130.85, 130.47, 128.43, 127.52, 126.43, 125.43, 125.30, 123.18, 122.41, 122.13, 120.35, 119.68, 119.50, 119.16, 115.29, 114.41, 114.08, 112.29, 108.88, 60.18, 60.04, 59.77, 55.03, 54.85, 53.45, 51.99, 46.98, 44.81, 38.53, 38.45, 38.39, 36.12, 36.01, 34.79, 29.88, 29.82, 28.49, 28.39, 28.28, 25.93, 25.46, 23.47, 23.27, 21.97, 21.43, 21.00, 19.04, 18.63, 16.97; HRMS (ESI): m/z $[M+H]^+$ calcd for $C_{65}H_{85}N_{10}O_{13}$: 1213.6298; found: 1213.6300.

*Ilamcyin NJL16 (**16**).* Yellow solid; 35% yield; $[\alpha]_D^{25}$ -12.2(c 0.1, MeOH); ^{1}H NMR (700 MHz, CD_3OD) δ 9.27 (d, J = 1.4 Hz, 1H), 8.83 (d, J = 2.4 Hz, 1H), 8.75 (dd, J = 2.4, 1.5 Hz, 1H), 7.77 (d, J = 2.1 Hz, 1H), 7.55 (d, J = 7.7 Hz, 1H), 7.52 (d, J = 8.3 Hz, 1H), 7.40 (dd, J = 8.6, 2.2 Hz, 1H), 7.13 (s, 1H), 7.09 – 7.02 (m, 3H), 6.14 (dd, J = 17.5, 10.7 Hz, 1H), 5.52 – 5.47 (m, 1H), 5.23 – 5.15 (m, 3H), 4.98 (dd, J = 8.7, 5.2 Hz, 1H), 4.91 (d, J = 4.4 Hz, 1H), 4.80 (q, J = 6.8 Hz, 1H), 4.66 (dd, J = 11.7, 3.2 Hz, 1H), 4.63 – 4.60 (m, 1H), 4.60 – 4.58 (m, 1H), 4.58 – 4.56 (m, 1H), 3.41 (dd, J = 13.1, 11.4 Hz, 1H), 3.17 (dd, J = 12.7, 4.6 Hz, 1H), 3.02 (dd, J = 13.1, 9.9 Hz, 1H), 2.81 – 2.74 (m, 2H), 2.73 (s, 3H), 2.66 (s, 3H), 2.52 – 2.43 (m, 2H), 2.21 – 2.15 (m, 1H), 1.90 – 1.84 (m, 1H), 1.75 – 1.71 (m, 9H), 1.63 – 1.60 (m, 1H), 1.56 (d, J = 6.3 Hz, 3H), 1.26 (d, J = 6.9 Hz, 3H), 1.23 (d, J = 7.1 Hz, 3H), 1.11 – 1.05 (m, 1H), 0.98 (d, J = 5.9 Hz, 3H), 0.95 (d, J = 5.9 Hz, 3H), 0.38 (d, J = 6.7 Hz, 3H), 0.17 (d, J = 6.7 Hz, 3H); ^{13}C NMR (176 MHz, CD_3OD) δ 179.93, 174.89, 173.99, 173.88, 173.23, 172.33, 170.89, 169.62, 154.67, 149.29, 147.07, 145.82, 145.43, 145.37, 144.54, 142.59, 139.17, 136.92, 135.53, 131.30, 130.45, 129.54, 126.74, 125.42, 125.28, 122.13, 121.14, 120.34, 119.50, 115.29, 114.08, 108.93, 60.18, 59.97, 59.75, 55.45, 54.84, 53.40, 51.92, 49.00, 46.83, 44.86, 38.45, 38.31, 38.14, 36.14, 34.72, 29.88, 29.76, 28.49, 28.39, 28.25, 25.96, 25.47, 23.48, 23.18, 22.02, 21.01, 18.97, 18.61, 16.93; HRMS (ESI): m/z $[M+H]^+$ calcd for $C_{59}H_{78}N_{11}O_{13}$: 1148.5781; found: 1148.5795.

*Ilamcyin NJL17 (**17**).* Yellow solid; 40% yield; $[\alpha]_D^{25}$ -12.6(c 0.1, MeOH); ^{1}H NMR (700 MHz, CD_3OD) δ 7.82 (s, 1H), 7.54 (d, J = 7.8 Hz, 1H), 7.51 (d, J = 8.4 Hz, 2H), 7.40 – 7.37 (m, 2H), 7.11 (s, 1H), 7.09 – 7.01 (m, 5H), 6.13 (dd, J = 17.4, 10.7 Hz, 1H), 5.53 – 5.46 (m, 1H), 5.23 – 5.15 (m, 3H), 4.97 (dd, J = 8.3, 5.1 Hz, 1H), 4.89 – 4.88 (m, 1H), 4.80 (dd, J = 13.8, 7.0 Hz, 1H), 4.66 – 4.59 (m, 3H), 4.57 (t, J = 4.5 Hz, 1H), 3.97 (s, 2H), 3.41 – 3.36 (m, 1H), 3.18 (d, J = 4.4 Hz, 1H), 3.17 – 3.12 (m, 1H), 2.83 – 2.76 (m, 2H), 2.72 (s, 3H), 2.58 (s, 3H), 2.54 – 2.48 (m, 1H), 2.47 – 2.42 (m, 1H), 2.21 – 2.14 (m, 1H), 1.89 – 1.83 (m, 1H), 1.72 – 1.65 (m, 9H), 1.62 – 1.59 (m, 1H), 1.28 (d, J = 7.1 Hz, 3H), 1.23 (d, J = 7.1 Hz, 3H), 1.10 – 1.04 (m, 1H), 0.94 (dd, J = 12.2, 5.6 Hz, 6H), 0.37 (d, J = 6.7 Hz, 3H), 0.16 (d, J = 6.7 Hz, 3H); ^{13}C NMR (176 MHz, CD_3OD) δ 180.64, 175.01, 174.01, 173.89, 173.18, 172.29, 170.92, 170.81, 169.68, 162.93, 145.42, 144.16, 138.17, 136.91, 136.83, 132.66, 131.32, 130.85, 130.46, 129.53, 127.63, 126.33, 125.43, 125.30, 122.13, 120.34, 119.50, 116.18, 115.28, 114.08, 108.88, 60.18, 60.04, 59.76, 55.02, 54.84, 53.45, 51.99, 46.98, 44.80, 40.46, 38.53, 38.46, 38.38, 36.11, 34.82, 29.86, 29.80, 28.48, 28.39, 28.27, 25.92, 25.46, 23.47, 23.26, 21.96, 21.00, 19.05, 18.62, 16.96; HRMS (ESI): m/z $[M+H]^+$ calcd for $C_{62}H_{81}FN_9O_{13}$: 1178.5938; found: 1178.5962.

*Ilamcyin NJL18 (**18**).* Yellow solid; 32% yield; $[\alpha]_D^{25}$ -53.4(c 0.1, MeOH); ^{1}H NMR (700 MHz, CD_3OD) δ 7.83 (d, J = 1.9 Hz, 1H), 7.55 (d, J = 7.8 Hz, 1H), 7.54 – 7.51 (m, 2H), 7.27 (d, J = 8.3 Hz, 1H), 7.12 (s, 1H), 7.09 – 7.03 (m, 2H), 6.14 (dd, J = 17.5, 10.7 Hz, 1H), 5.54 – 5.48 (m, 1H), 5.25 – 5.16 (m, 3H), 4.98 (dd, J = 8.6, 4.9 Hz, 1H), 4.92 – 4.90 (m, 1H), 4.81 (dd, J = 13.9, 6.9 Hz, 1H), 4.67 – 4.60 (m, 3H), 4.58 (t, J = 4.5 Hz, 1H), 3.39 (dd, J = 13.1, 11.5 Hz, 1H), 3.19 (d, J = 4.6 Hz, 1H), 3.18 – 3.14 (m, 1H), 2.95 (t, 2H), 2.85 (t, J = 7.2 Hz, 2H), 2.83 – 2.78 (m, 2H), 2.73 (s, 3H), 2.60 (s, 3H), 2.55 – 2.50 (m, 1H), 2.49 – 2.42 (m, 1H), 2.21 – 2.17 (m, 1H), 2.16 (s, 3H), 1.89 – 1.83 (m, 1H), 1.73 – 1.66 (m, 9H), 1.64 – 1.60 (m, 1H), 1.59 (d, J = 6.4 Hz, 3H), 1.29 (d, J = 6.9 Hz, 3H), 1.23 (d, J = 7.1 Hz, 3H), 1.11 – 1.04 (m, 1H), 0.97 – 0.92 (m, 6H), 0.37 (d, J = 6.7 Hz, 3H), 0.16 (d, J = 6.6 Hz, 3H); ^{13}C NMR (176 MHz, CD_3OD) δ 180.38, 175.00, 174.03, 173.90, 173.18, 172.31, 171.18, 170.92, 169.68, 145.43, 144.07, 143.03, 137.08, 136.91, 131.34, 130.47, 130.38, 127.58, 126.46, 125.43, 125.31, 122.13, 120.35, 119.51, 115.29, 114.08, 108.89, 60.19, 60.04, 59.78, 55.02, 54.85, 53.45, 52.00, 46.98, 44.83, 38.54, 38.38, 36.13, 35.28, 34.81, 29.85, 29.80, 29.48, 28.49, 28.39, 28.29, 25.93, 25.47, 23.47, 23.26, 21.97, 21.00, 19.04, 18.62, 16.97, 15.29. HRMS (ESI): m/z $[M+H]^+$ calcd for $C_{58}H_{82}N_9O_{13}S$: 1144.5753; found: 1144.5753.

References

Sun, C., Liu, Z., Zhu, X., Fan, Z., Huang, H., Wu, Q., et al. (2020). Antitubercular Ilamycins from Marine-Derived *Streptomyces atratus* SCSIO ZH16 Δ *ilaR*. *J. Nat. Prod.* 83, 1646–1657. doi: 10.1021/acs.jnatprod.0c00151.

Xu, J., Liu, D., Niu, H., Zhu, G., Xu, Y., Ye, D., et al. (2017). Resveratrol reverses Doxorubicin resistance by inhibiting epithelial-mesenchymal transition (EMT) through modulating PTEN/Akt signaling pathway in gastric cancer. *J. Exp. Clin. Canc. Res*. 36, 1–14. doi: 10.1186/s13046-016-0487-8.

Zhang, T., Li, S. and Nuermberger, E. L. (2012). Autoluminescent *Mycobacterium Tuberculosis* for Rapid, Real-Time, Non-Invasive Assessment of Drug and Vaccine Efficacy. *PLoS One* 7, e29774. doi: 10.1371/journal.pone.0029774

Spectra Data

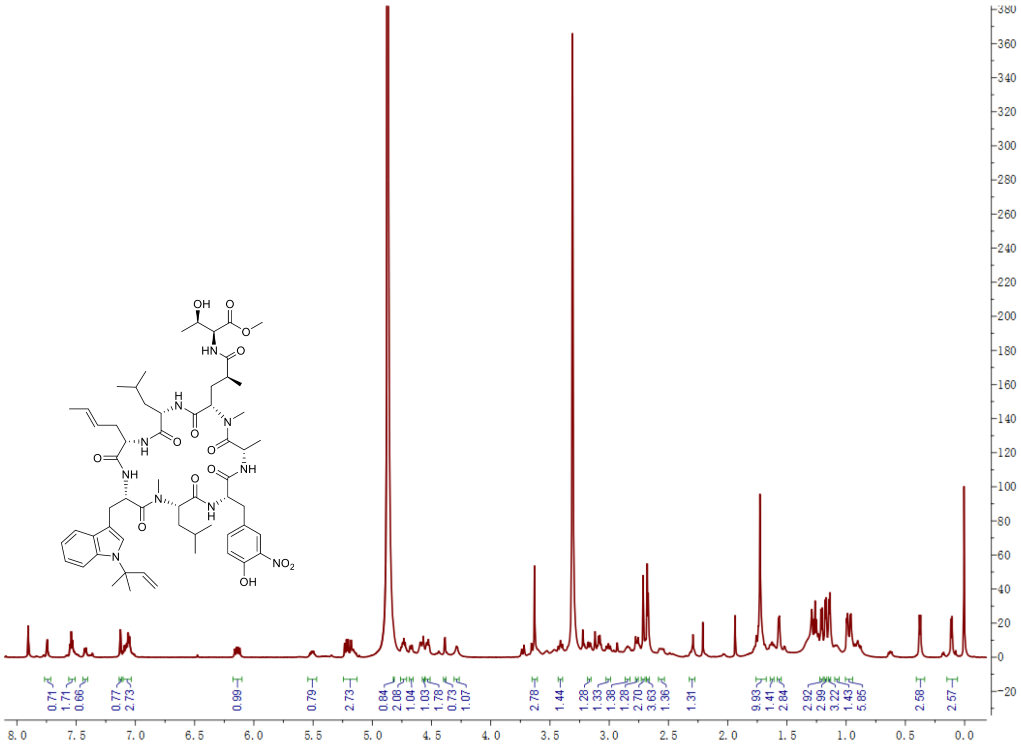

Figure S1. ^{1}H NMR spectrum of compound **1** in CD_3OD

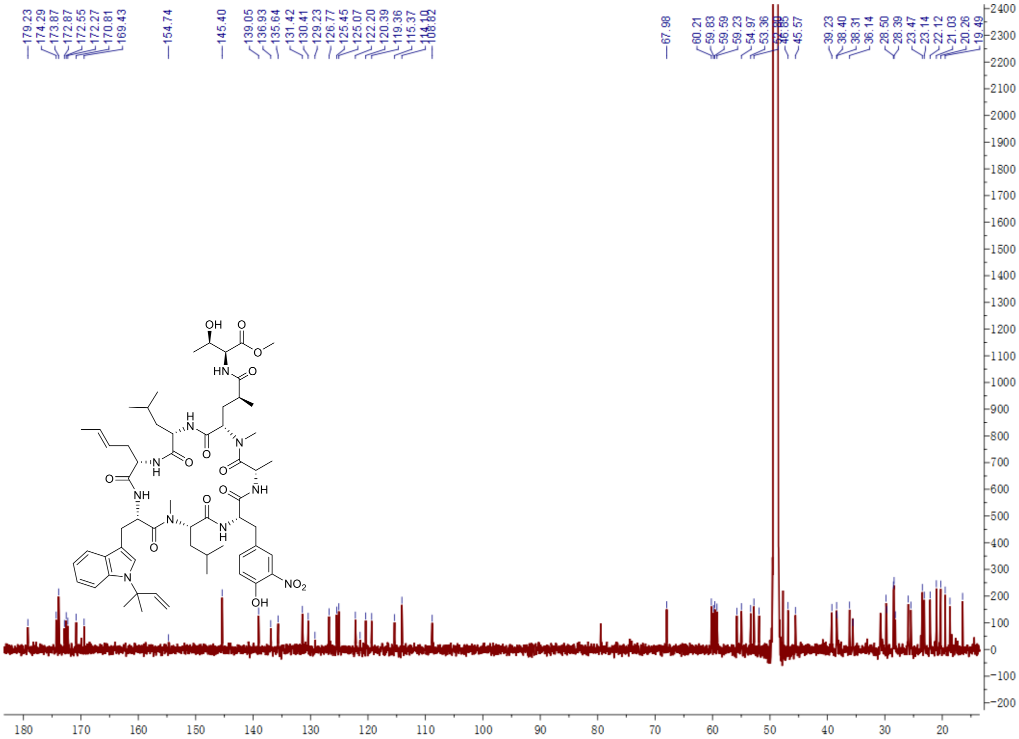

Figure S2. ^{13}C NMR spectrum of compound **1** in CD_3OD

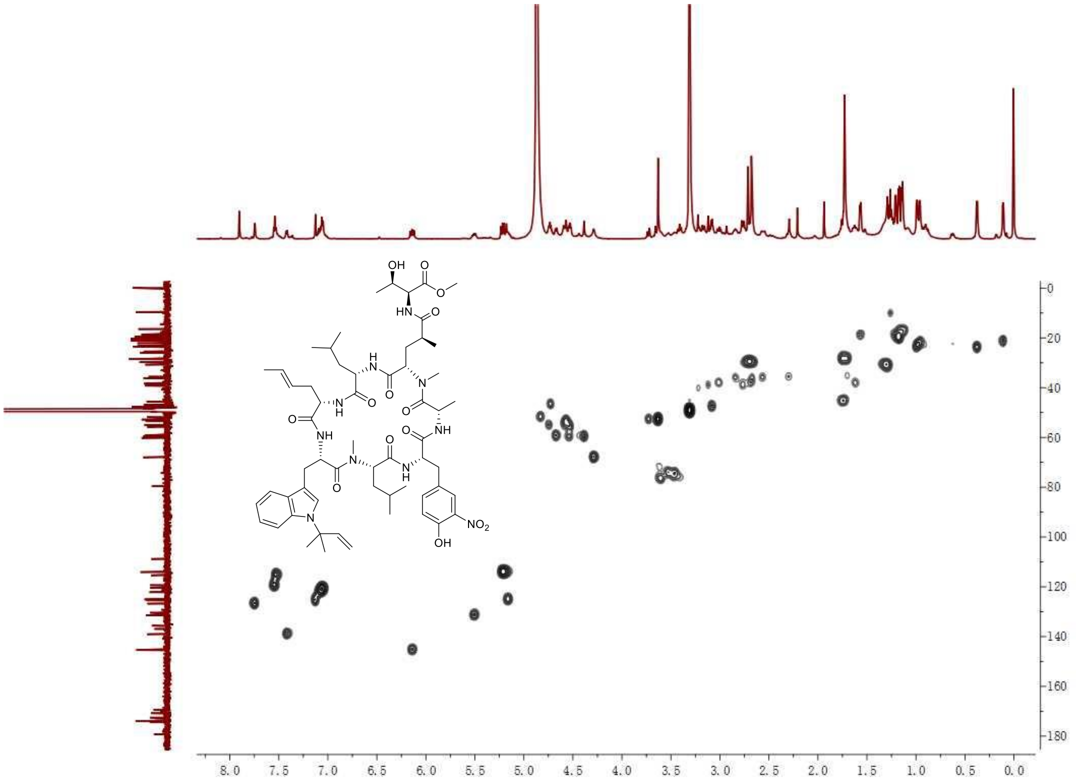

Figure S3. HSQC spectrum of compound **1** in CD_3OD

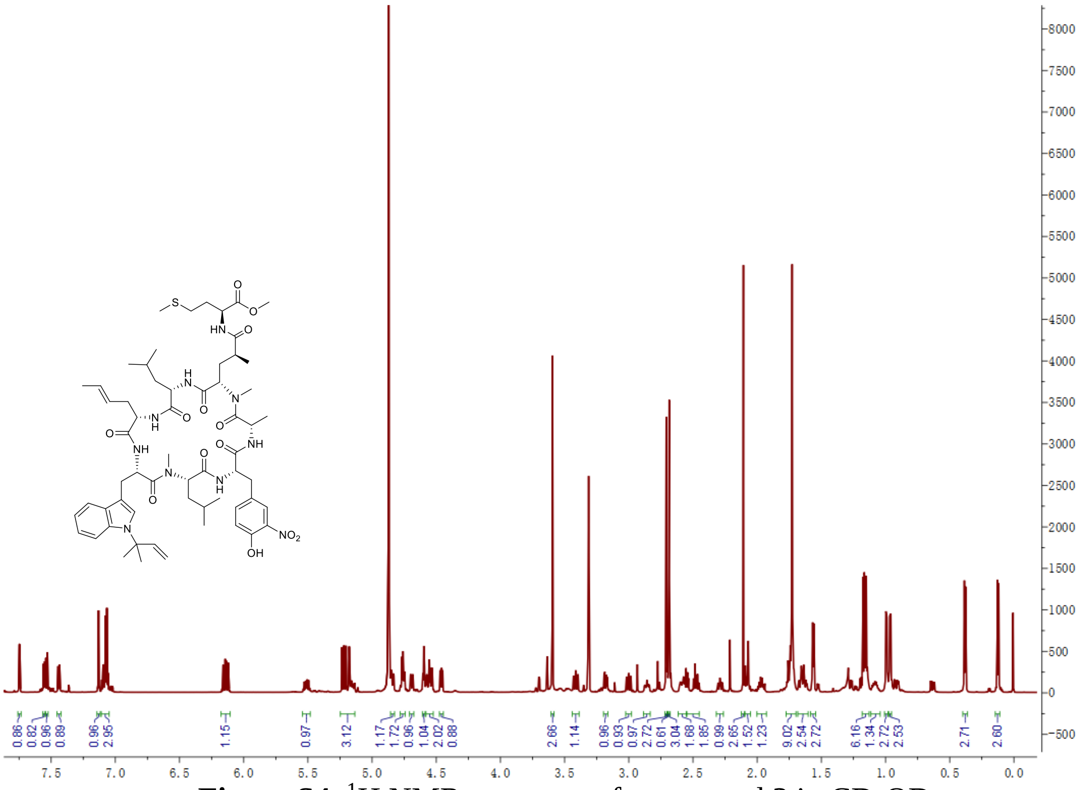

Figure S4. ^{1}H NMR spectrum of compound **2** in CD_3OD

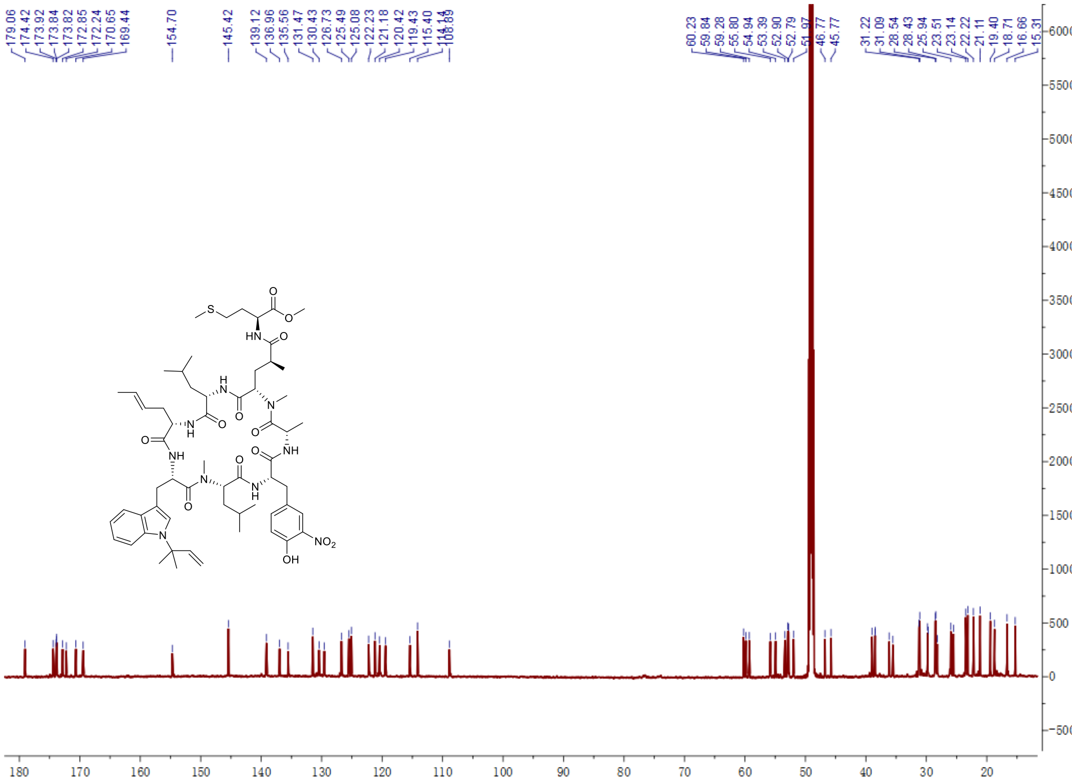

Figure S5. ^{13}C NMR spectrum of compound **2** in CD_3OD

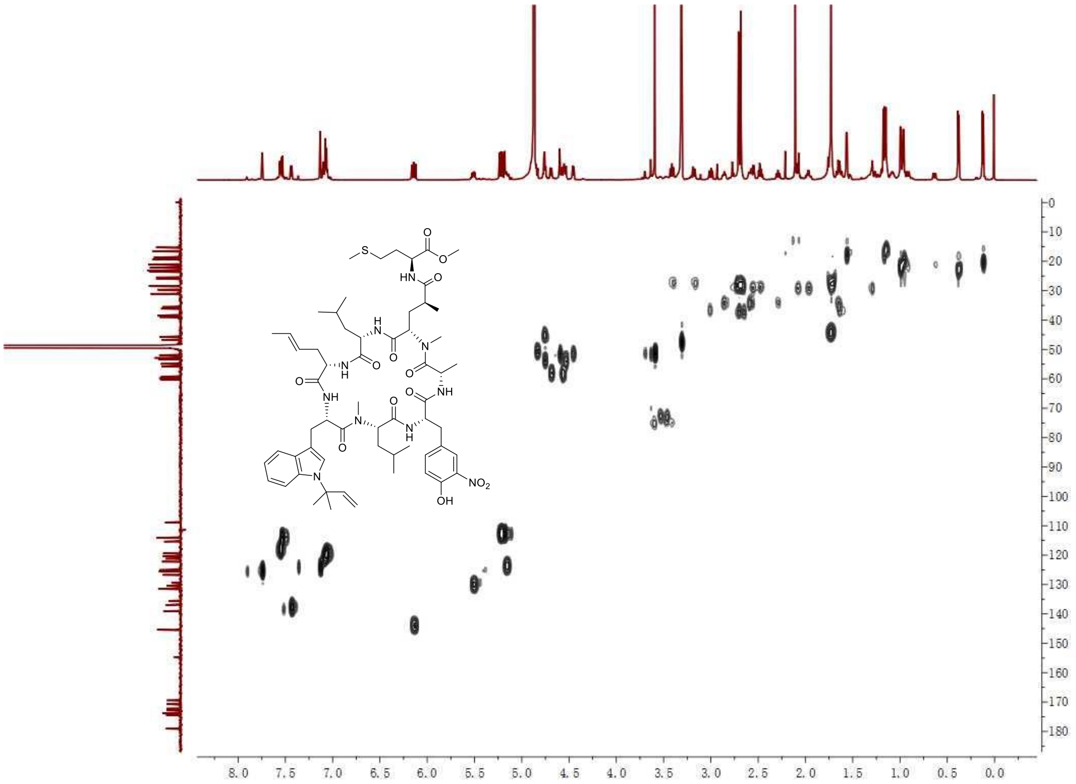

Figure S6. HSQC spectrum of compound **2** in CD_3OD

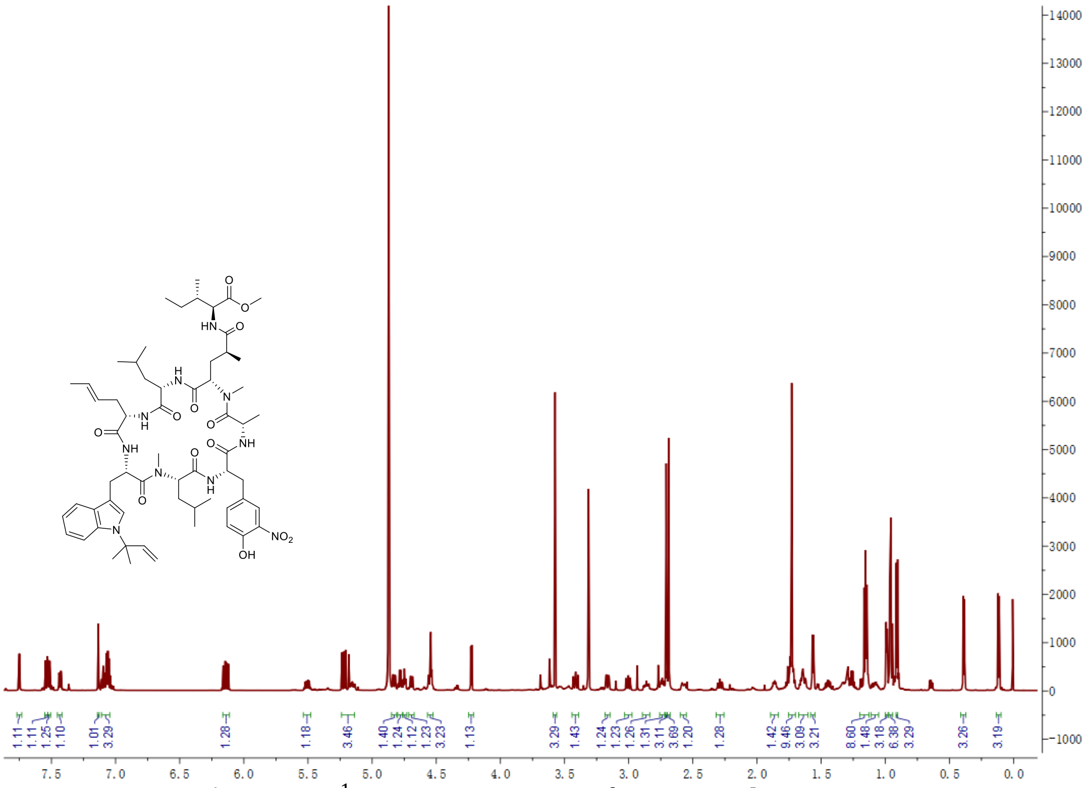

Figure S7. ^{1}H NMR spectrum of compound **3** in CD_3OD

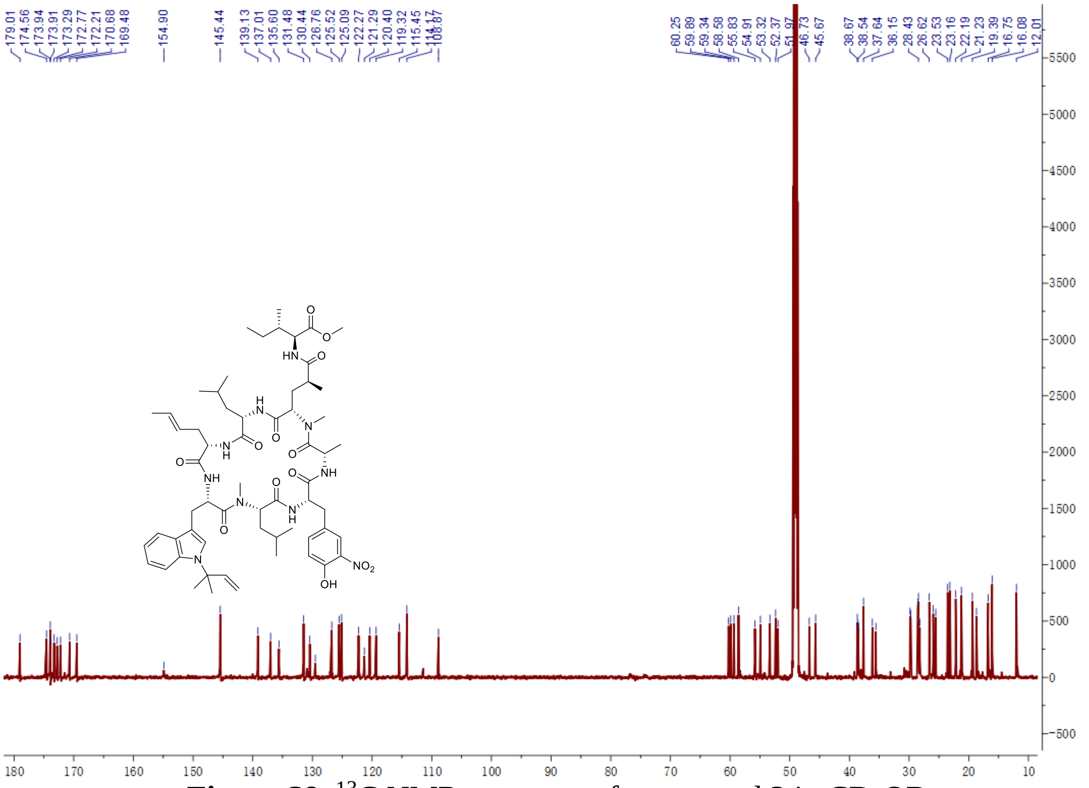

Figure S8. ^{13}C NMR spectrum of compound **3** in CD_3OD

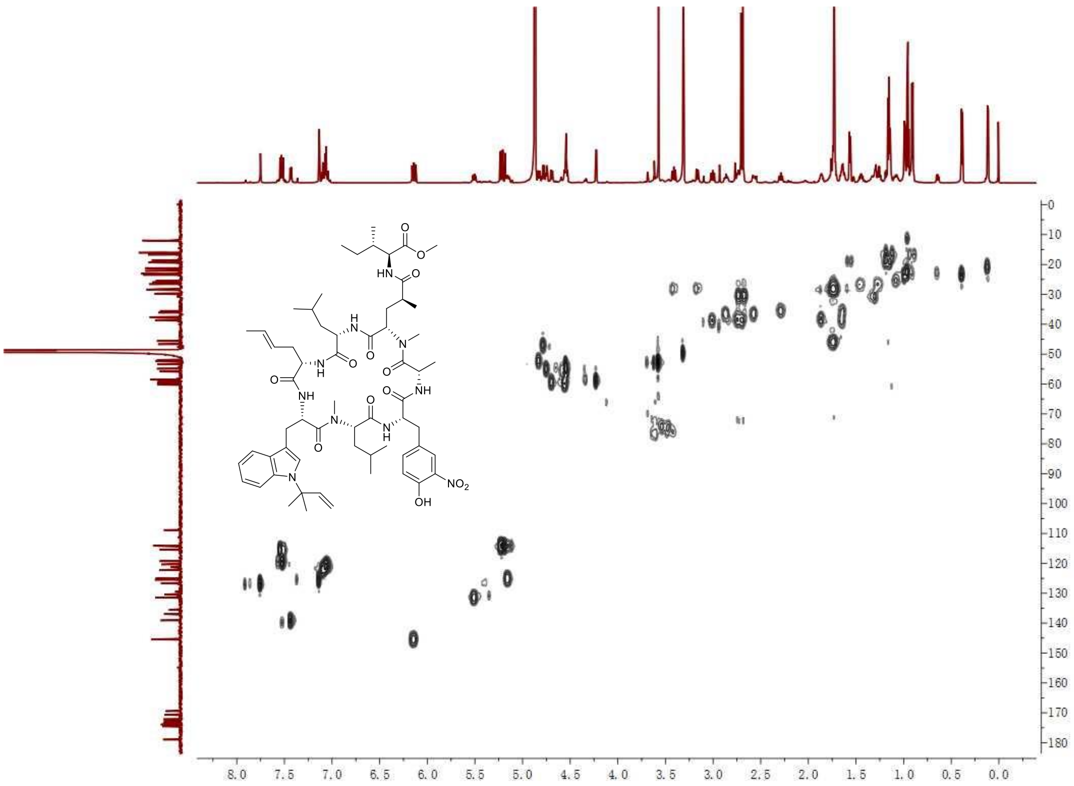

Figure S9. HSQC spectrum of compound **3** in CD_3OD

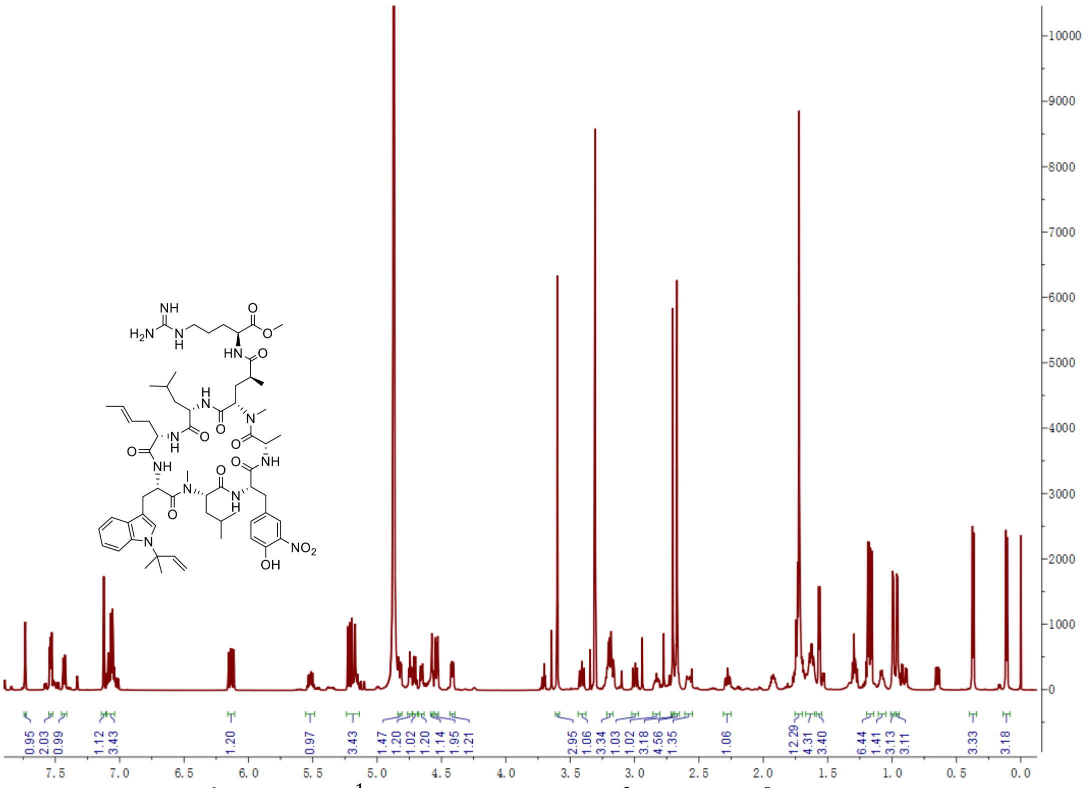

Figure S10. ^1H NMR spectrum of compound **4** in CD_3OD

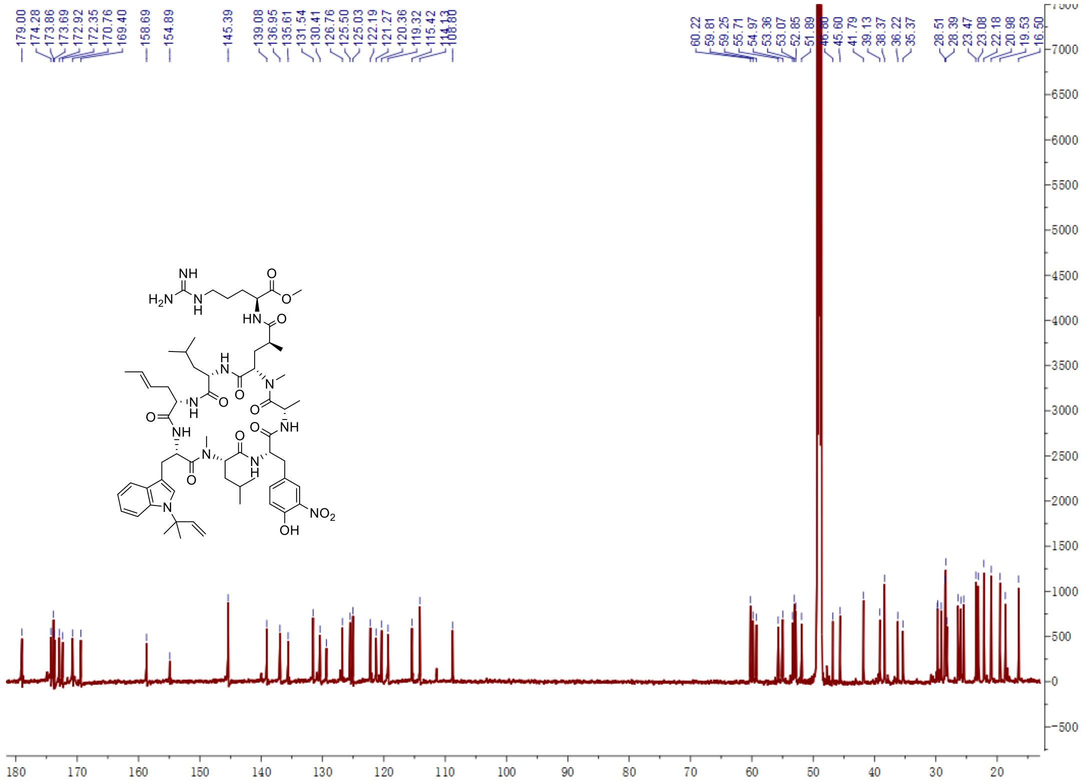

Figure S11. ^{13}C NMR spectrum of compound **4** in CD_3OD

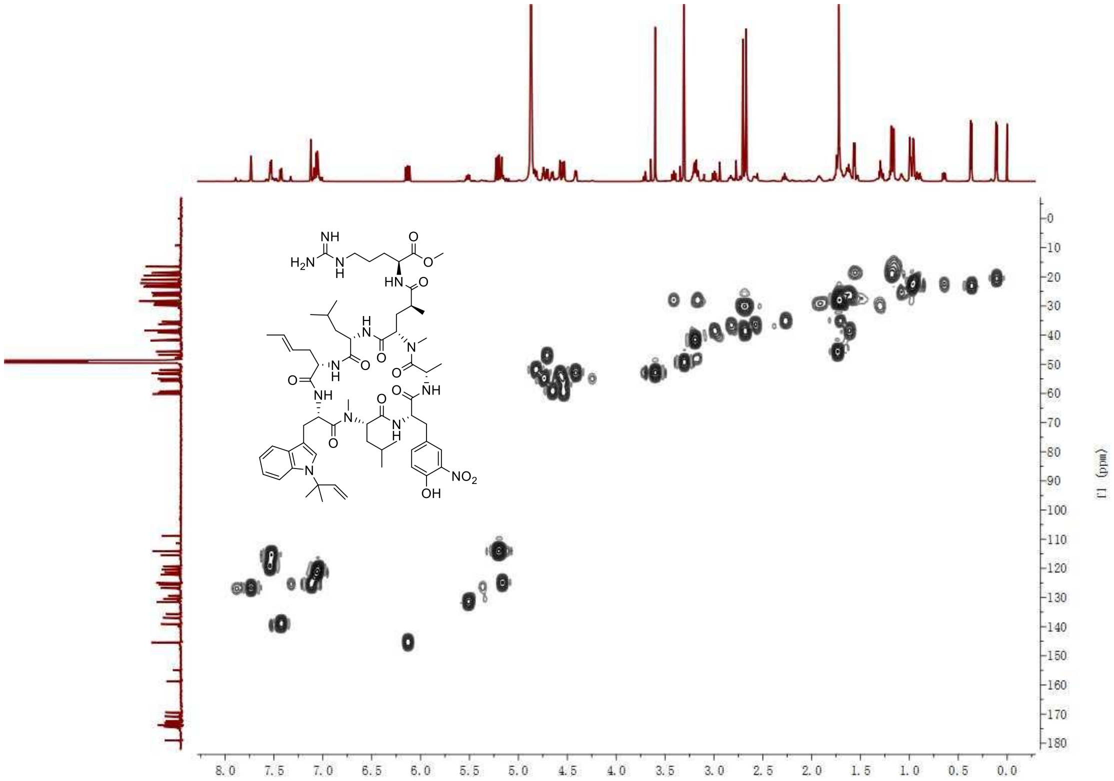

Figure S12. HSQC spectrum of compound **4** in CD_3OD

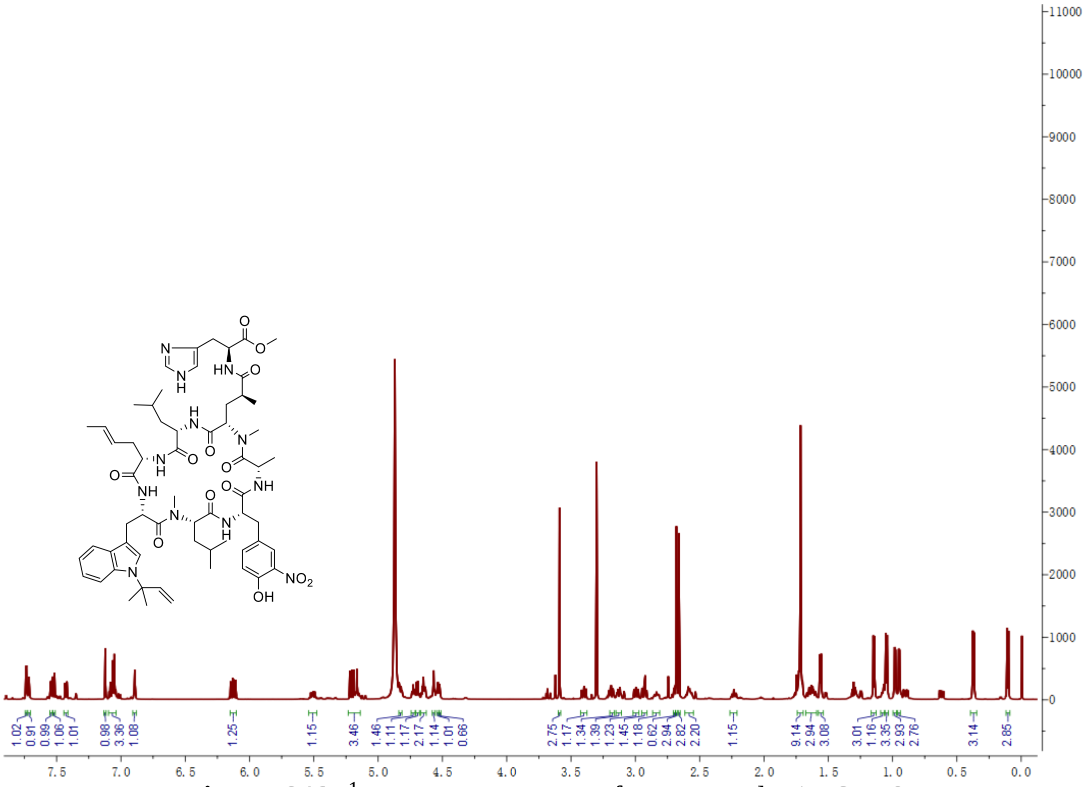

Figure S13. ^{1}H NMR spectrum of compound **5** in CD_3OD

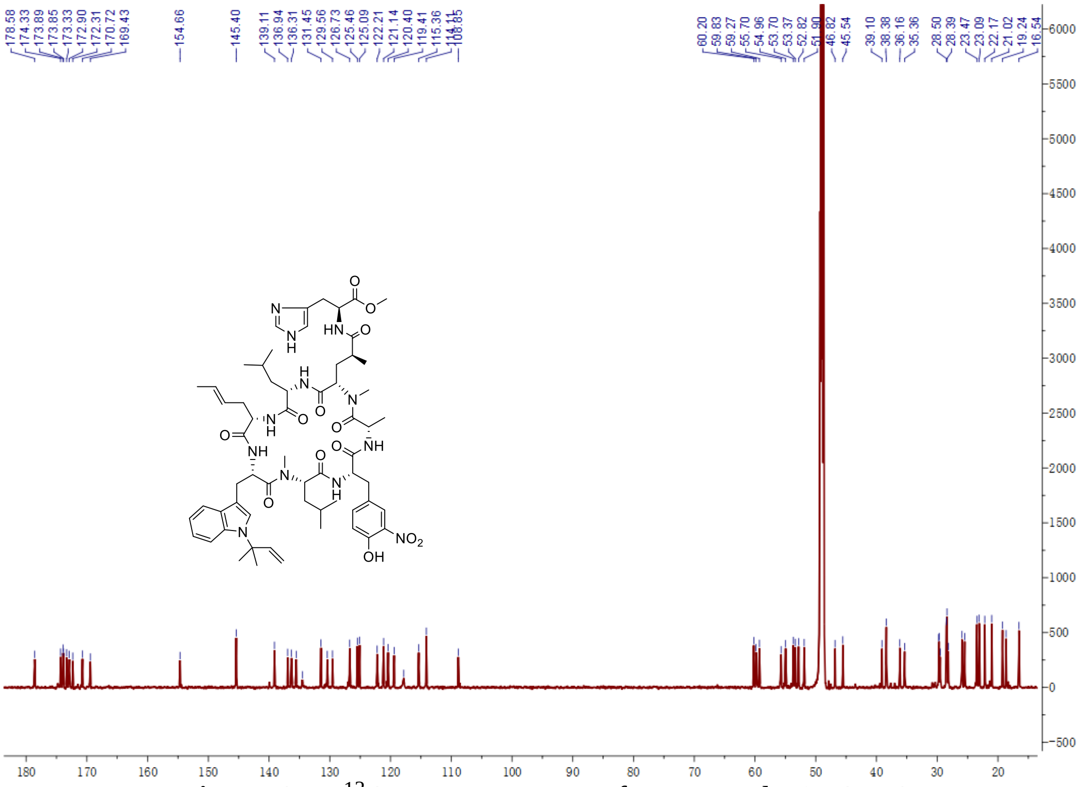

Figure S14. ^{13}C NMR spectrum of compound **5** in CD_3OD

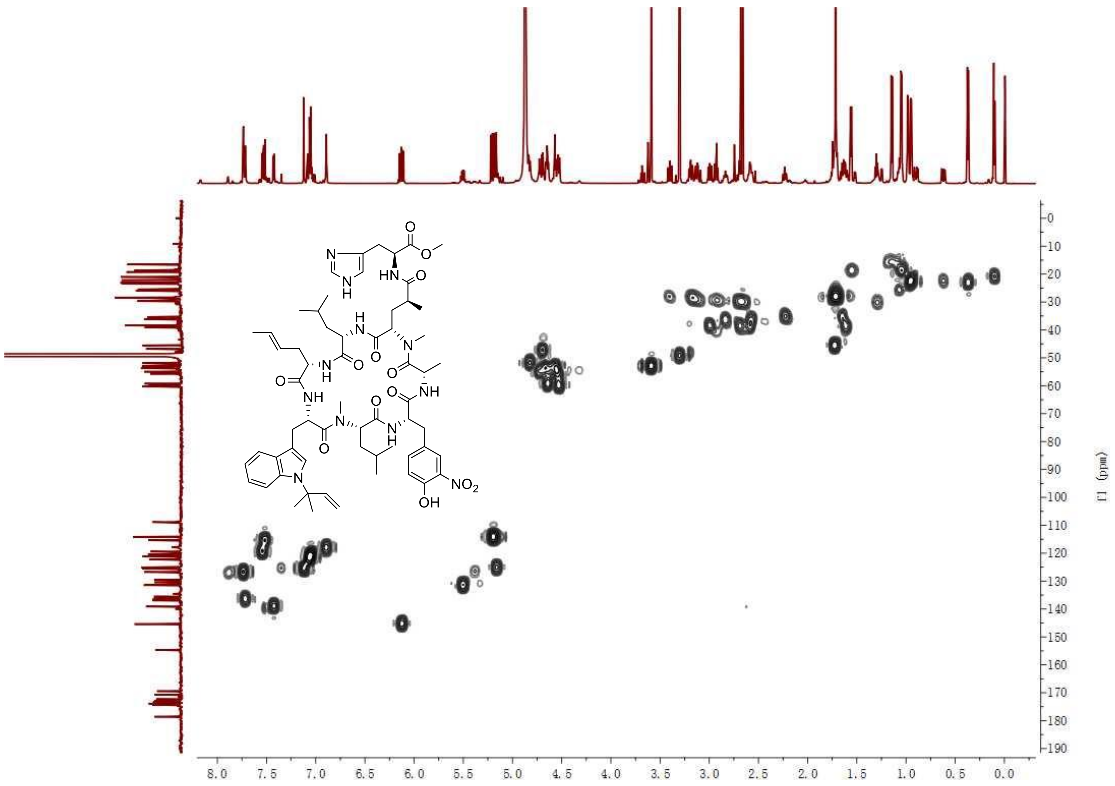

Figure S15. HSQC spectrum of compound **5** in CD_3OD

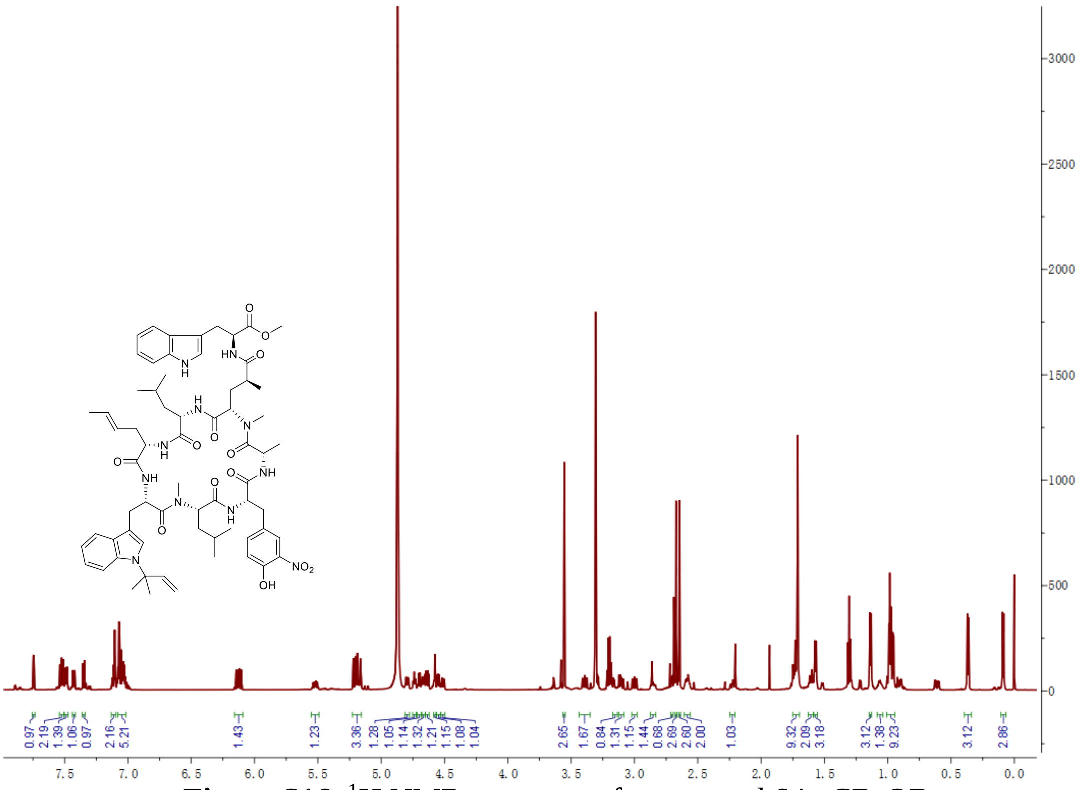

Figure S16. ^1H NMR spectrum of compound **6** in CD_3OD

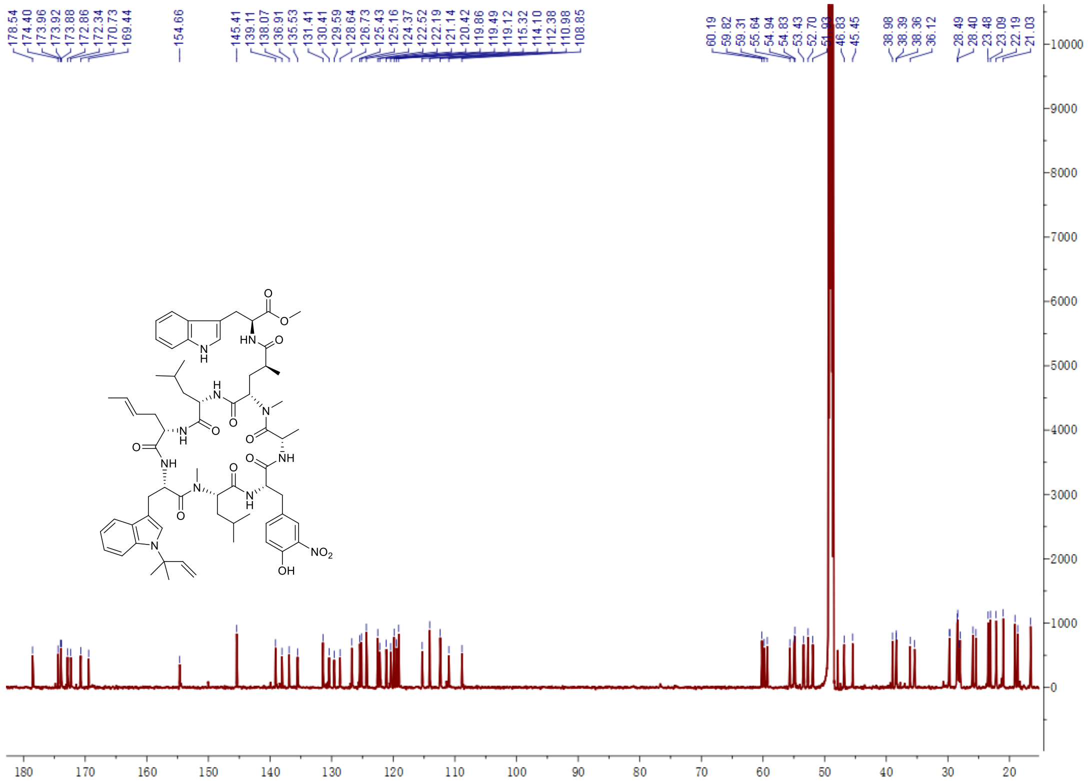

Figure S17. ^{13}C NMR spectrum of compound **6** in CD_3OD

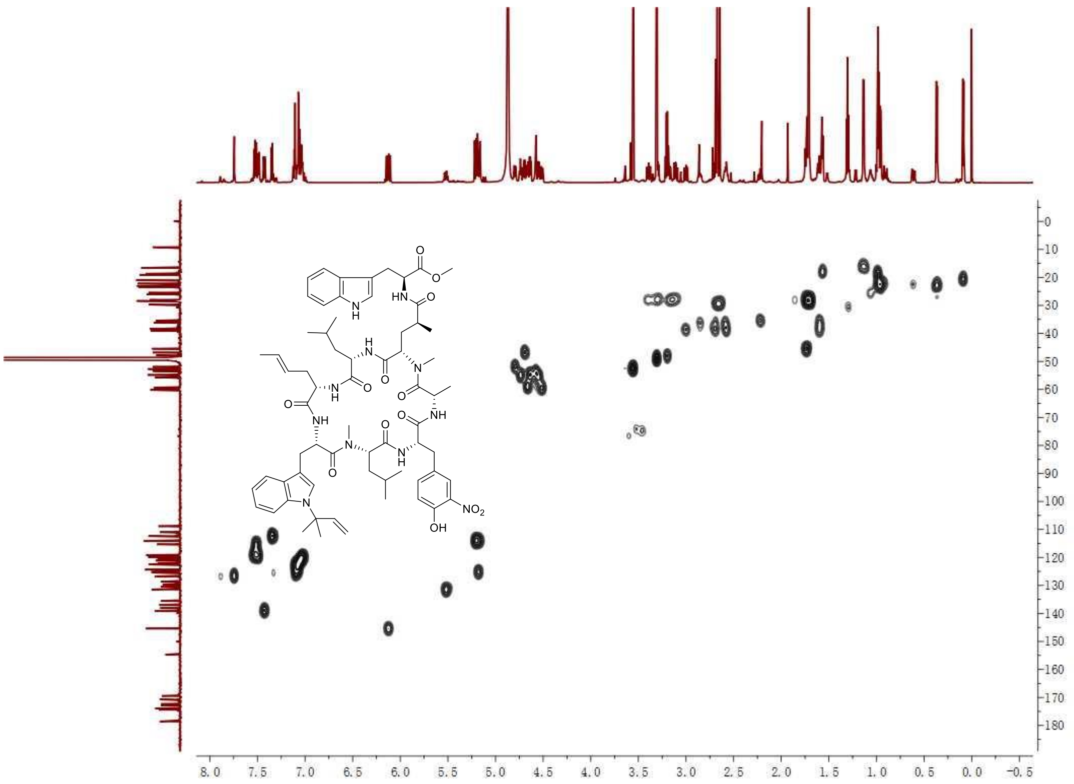

Figure S18. HSQC spectrum of compound **6** in CD_3OD

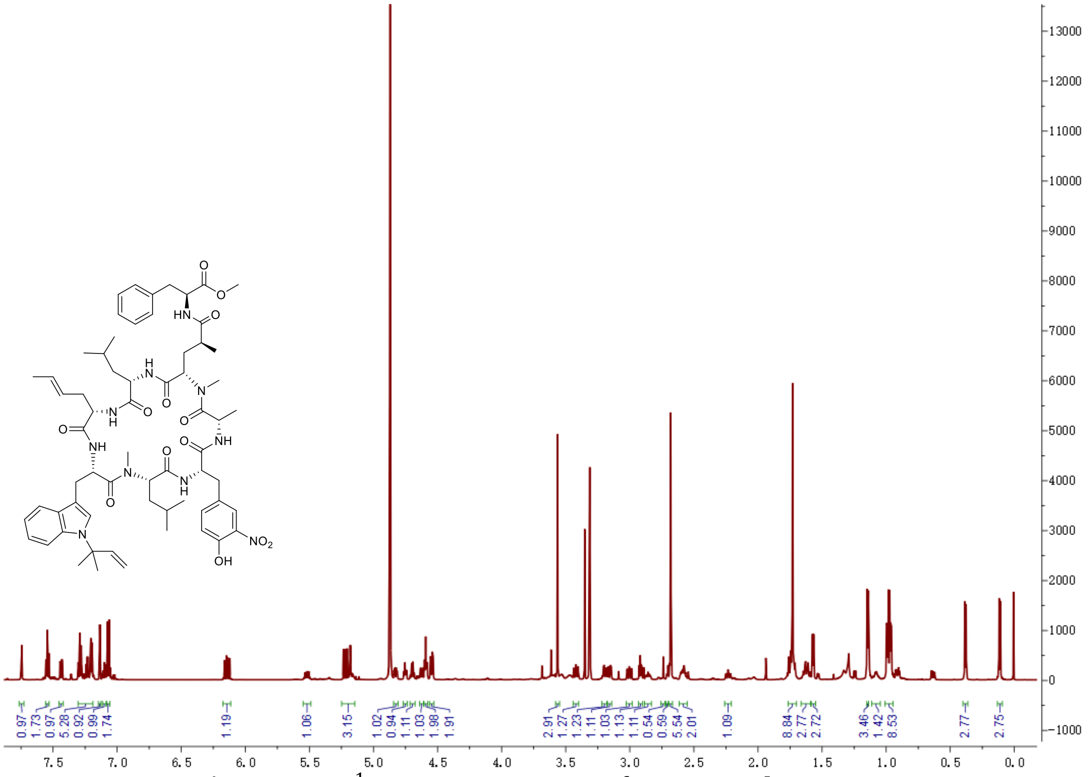

Figure S19. ^{1}H NMR spectrum of compound **7** in CD_3OD

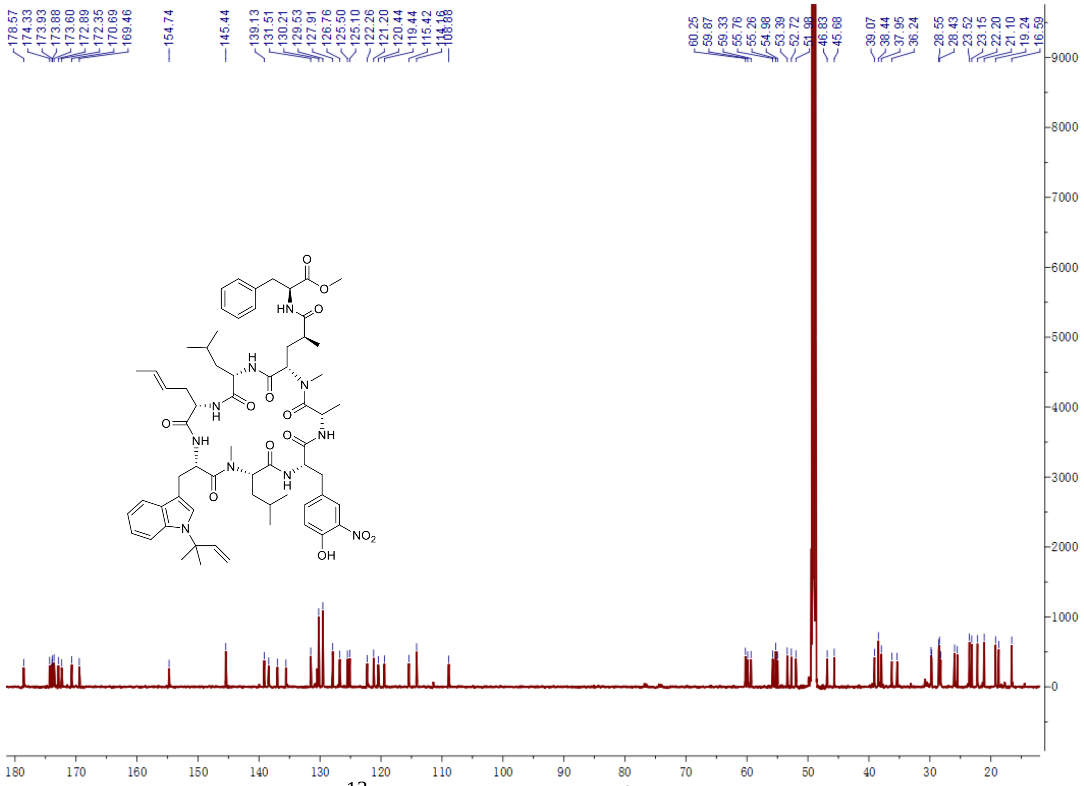

Figure S20. ^{13}C NMR spectrum of compound **7** in CD_3OD

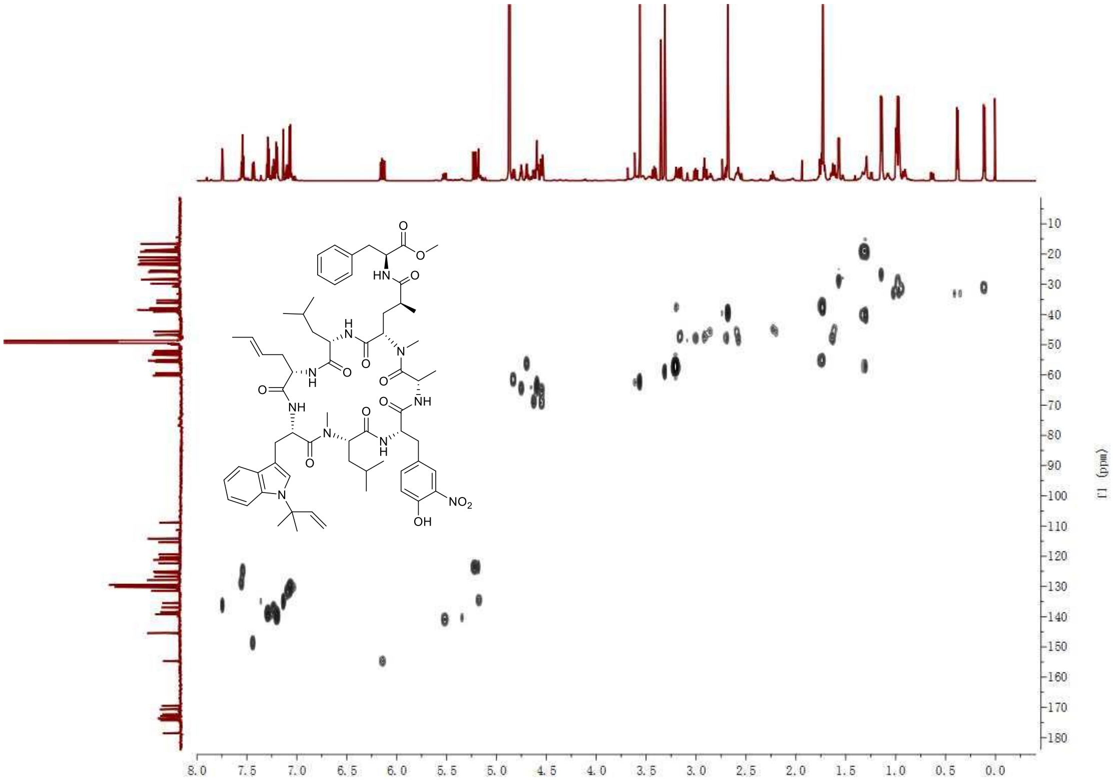

Figure S21. HSQC spectrum of compound **7** in CD_3OD

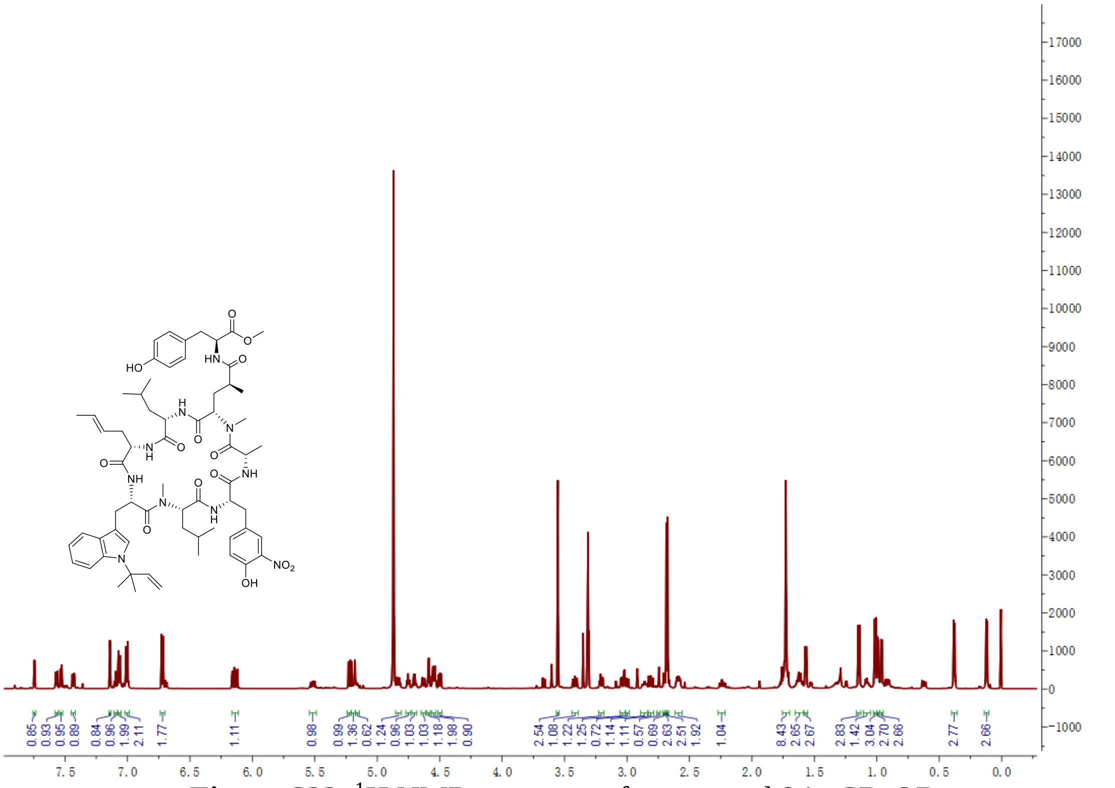

Figure S22. ^1H NMR spectrum of compound **8** in CD_3OD

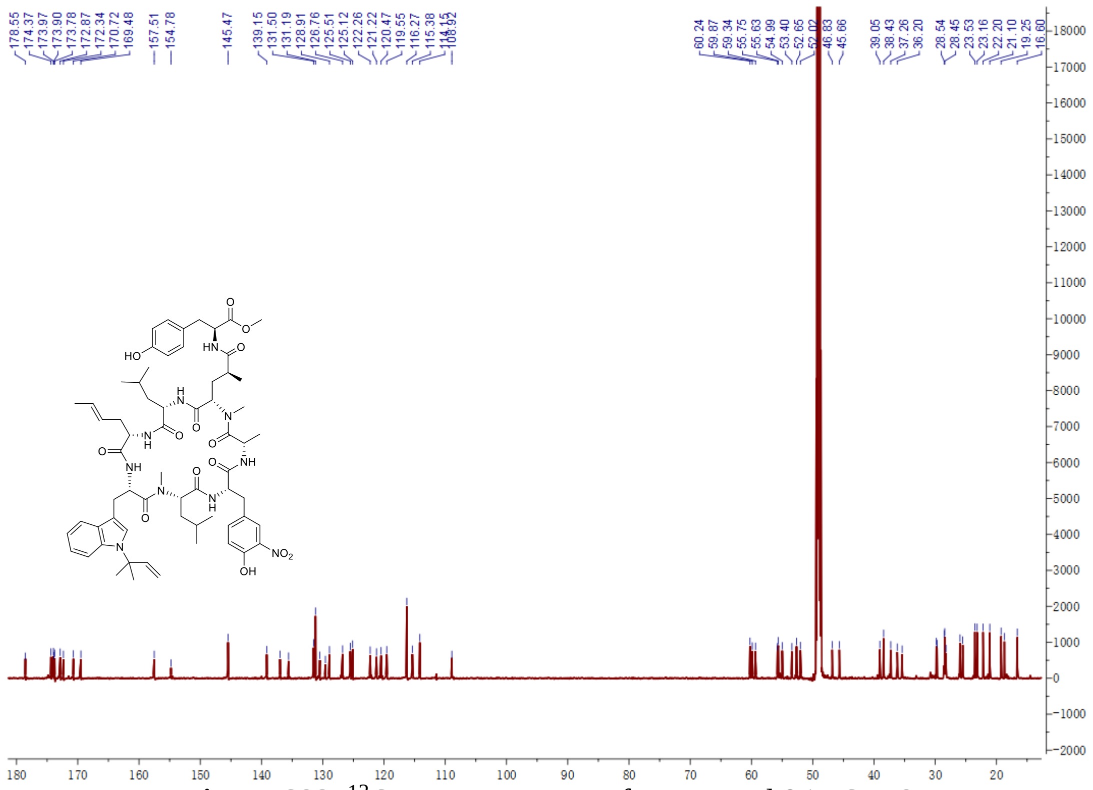

Figure S23. ^{13}C NMR spectrum of compound **8** in CD_3OD

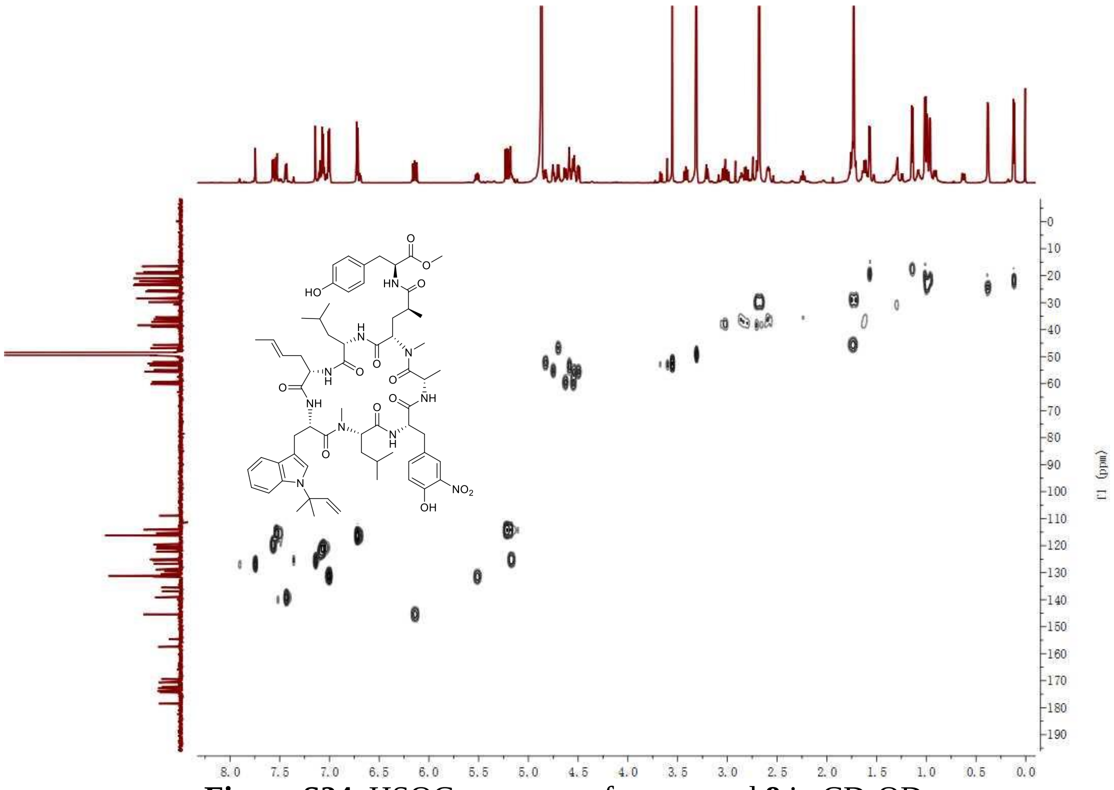

Figure S24. HSQC spectrum of compound **8** in CD_3OD

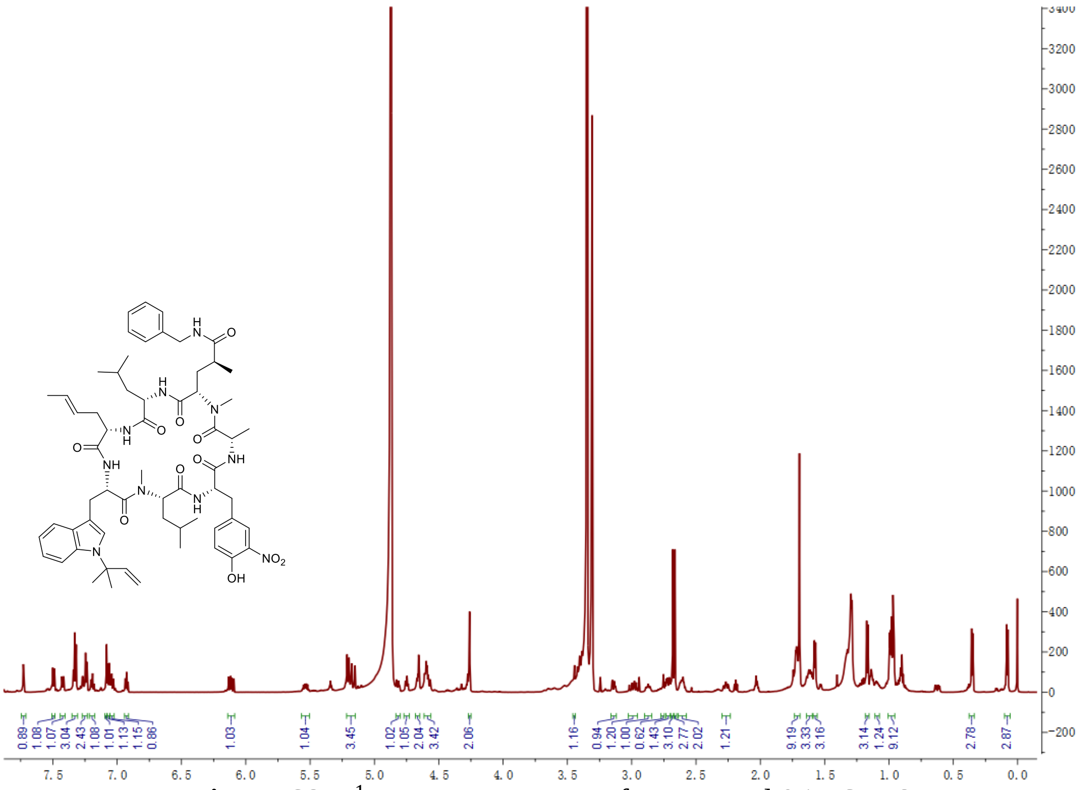

Figure S25. ^{1}H NMR spectrum of compound **9** in CD_3OD

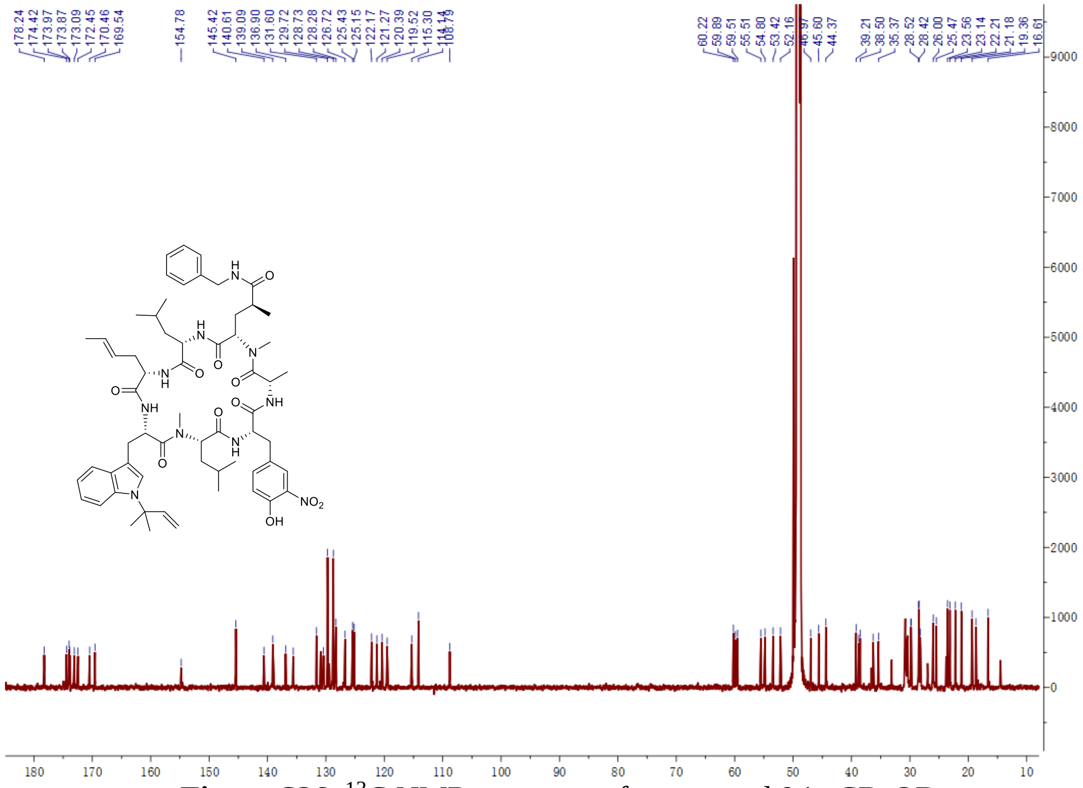

Figure S26. ^{13}C NMR spectrum of compound **9** in CD_3OD

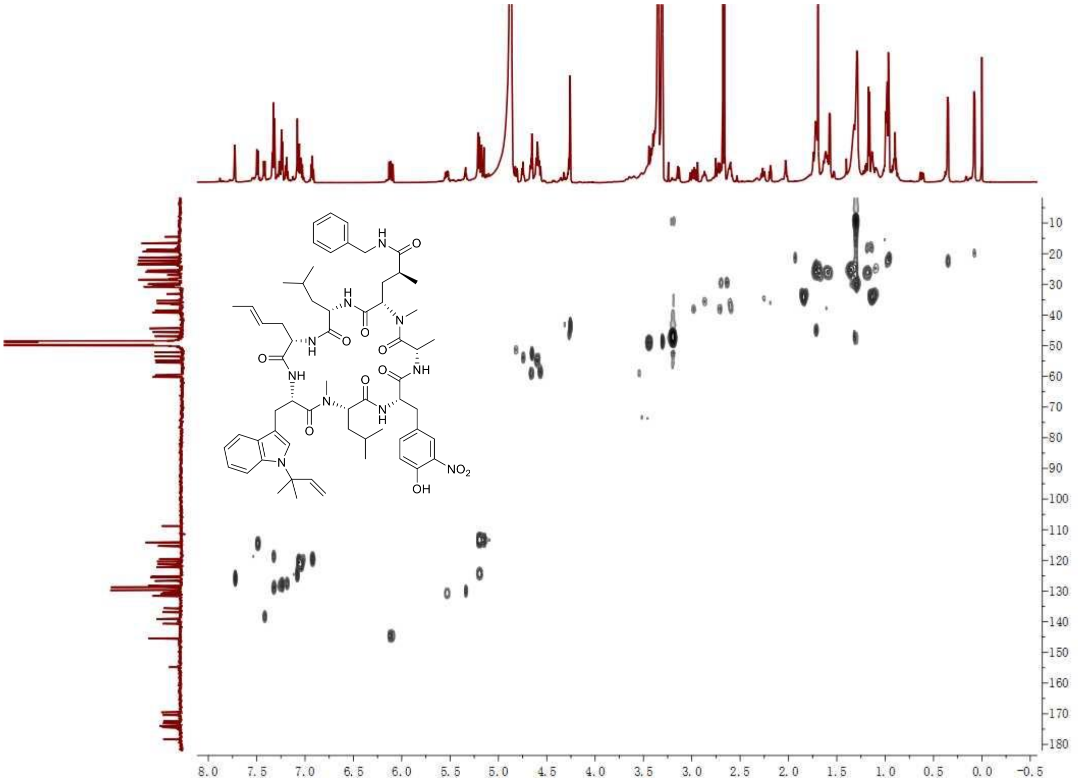

Figure S27. HSQC spectrum of compound **9** in CD_3OD

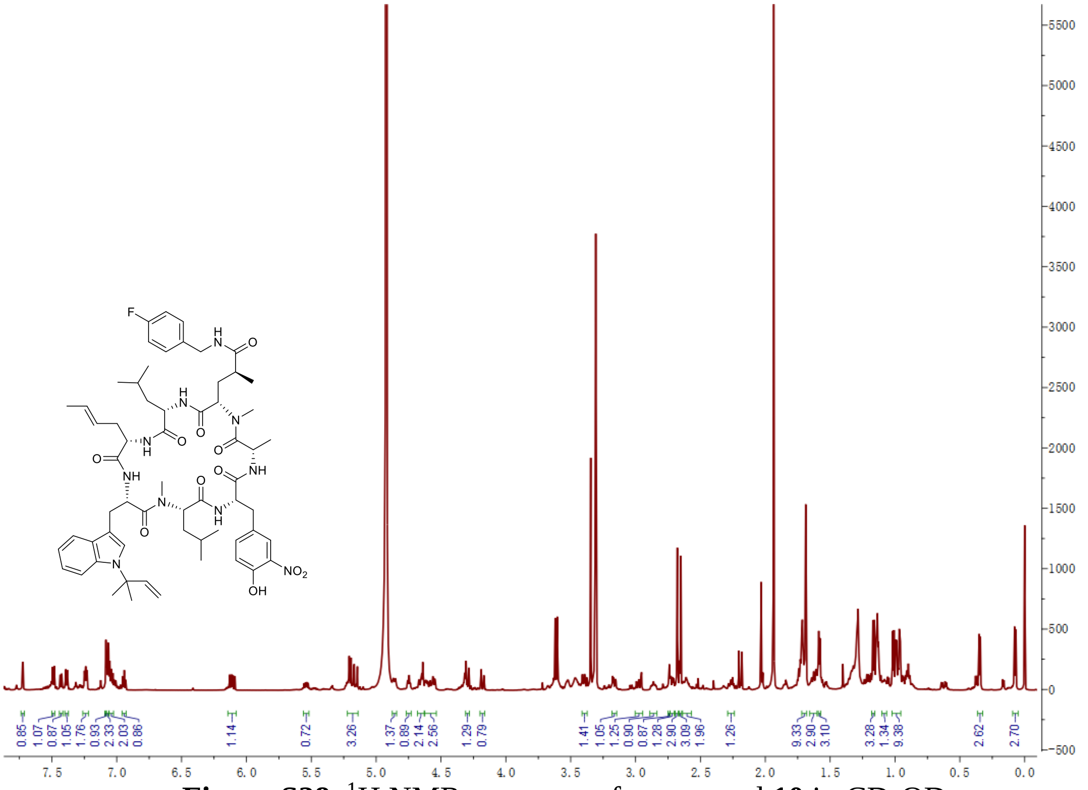

Figure S28. ^1H NMR spectrum of compound **10** in CD_3OD

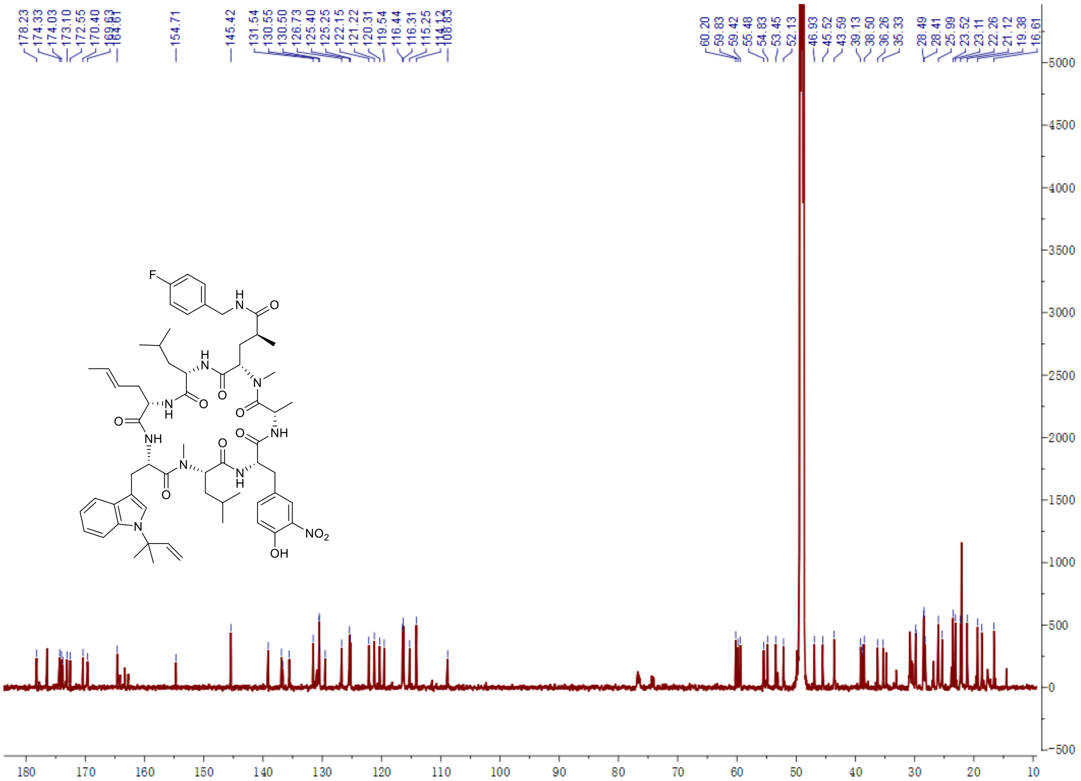

Figure S29. ^{13}C NMR spectrum of compound **10** in CD_3OD

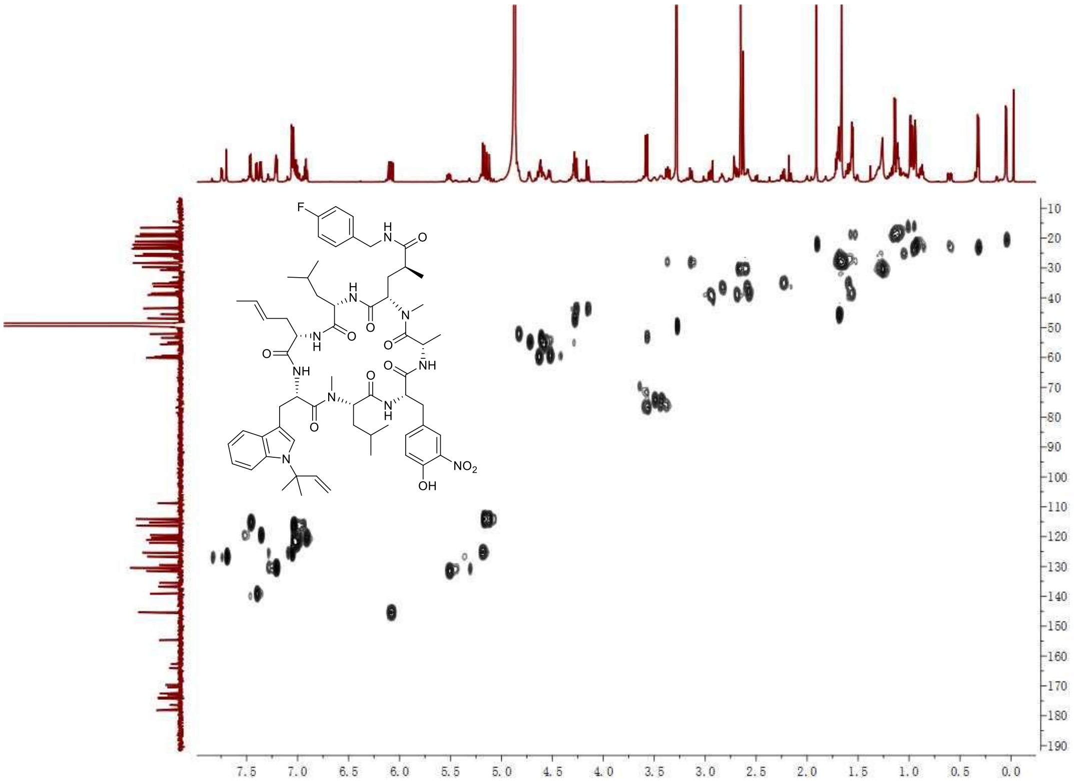

Figure S30. HSQC spectrum of compound **10** in CD_3OD

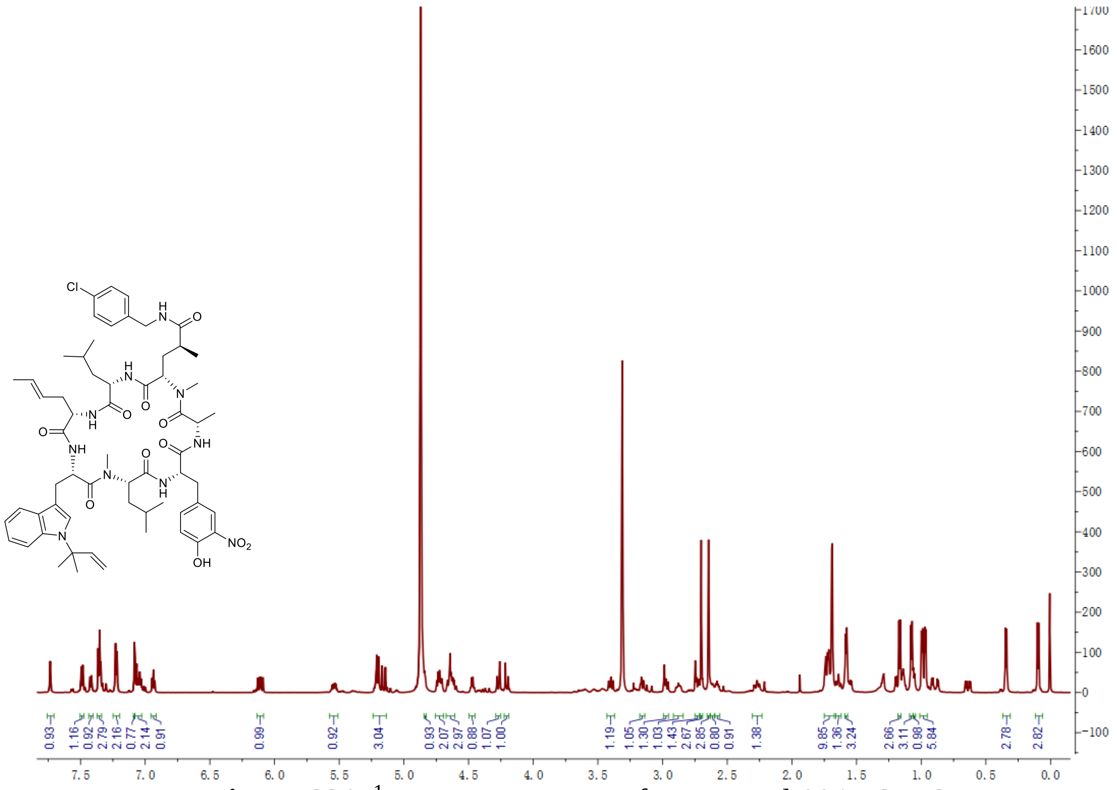

Figure S31. ^{1}H NMR spectrum of compound **11** in CD_3OD

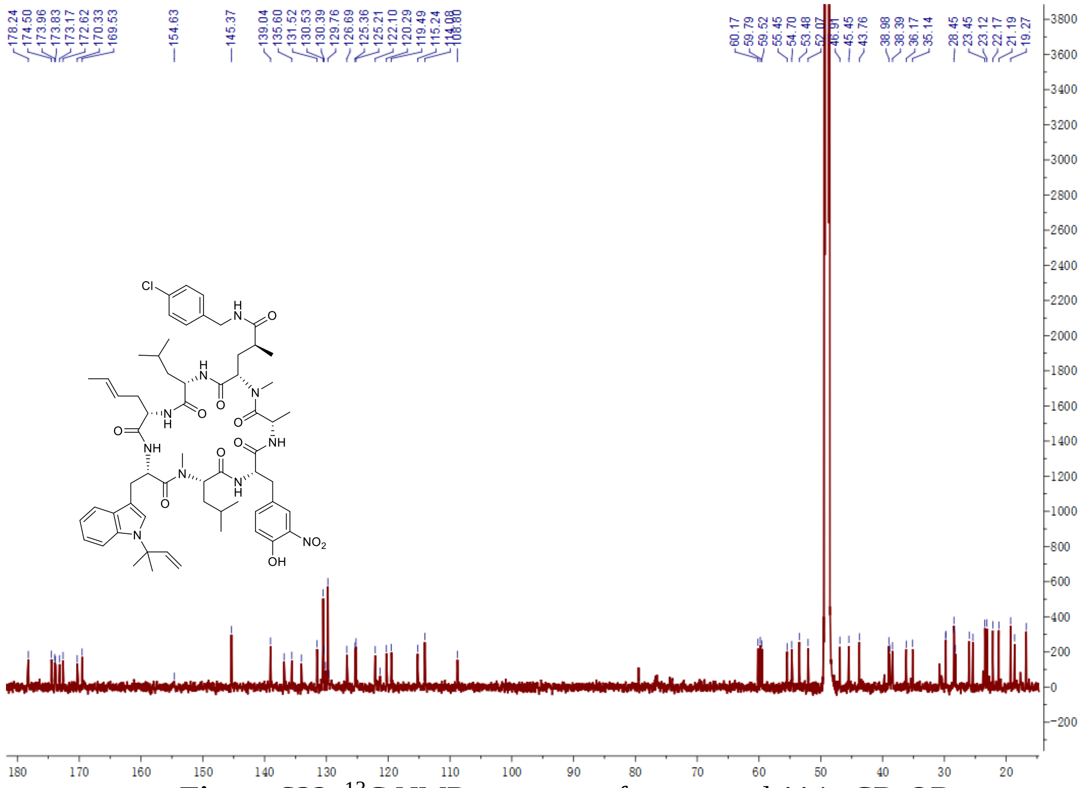

Figure S32. ^{13}C NMR spectrum of compound **11** in CD_3OD

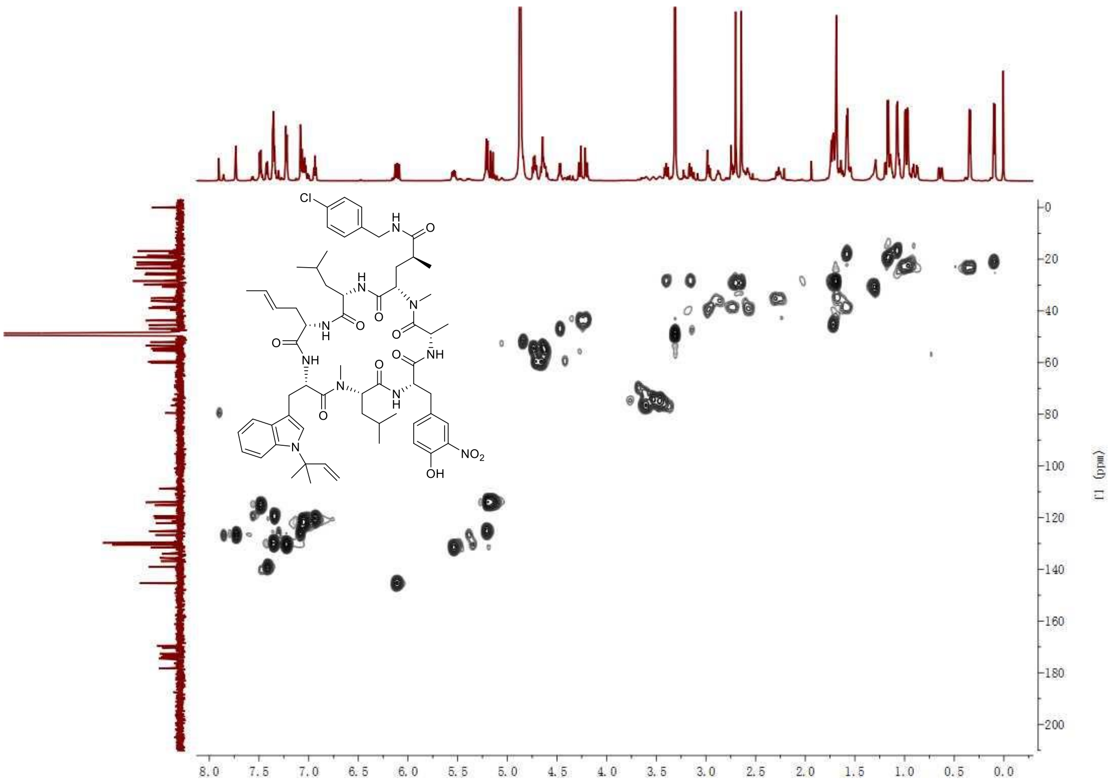

Figure S33. HSQC spectrum of compound **11** in CD_3OD

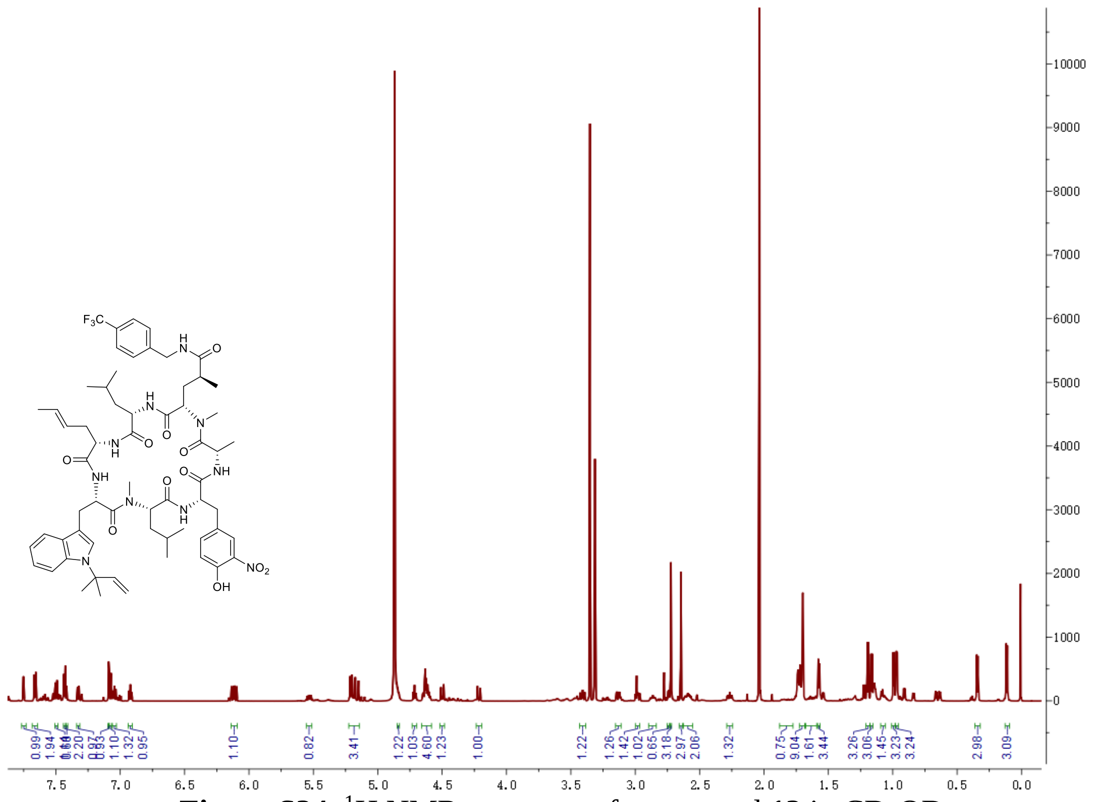

Figure S34. ^{1}H NMR spectrum of compound **12** in CD_3OD

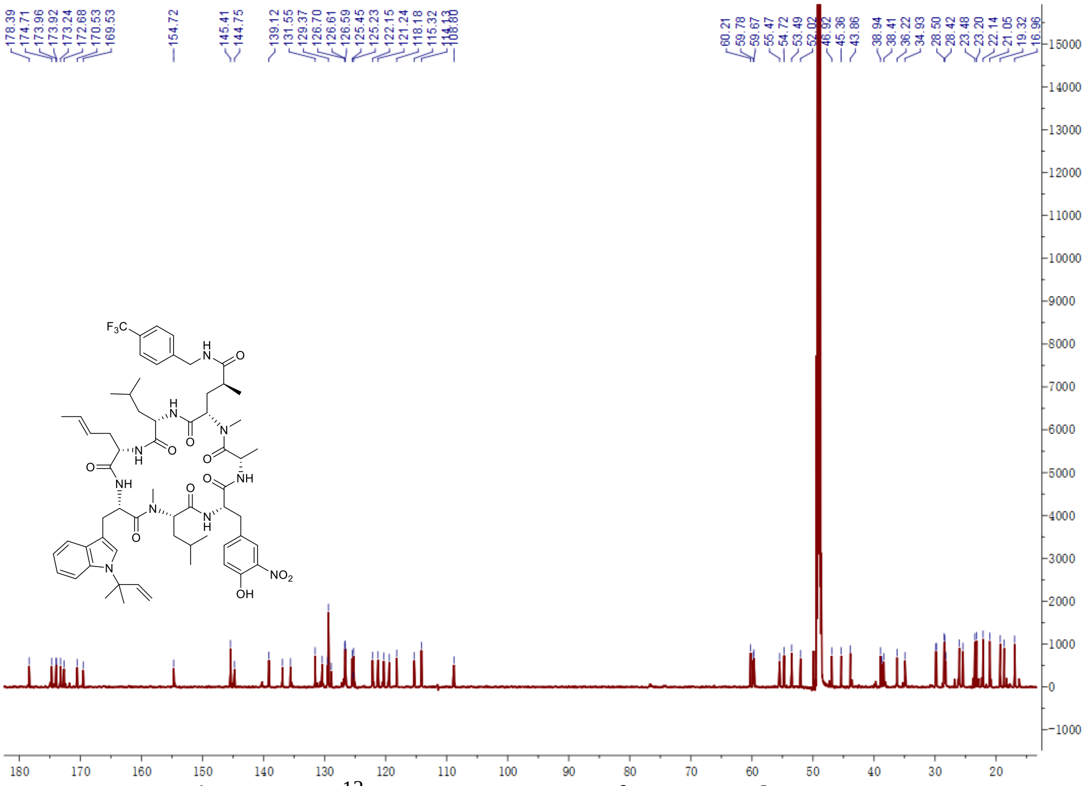

Figure S35. ^{13}C NMR spectrum of compound **12** in CD_3OD

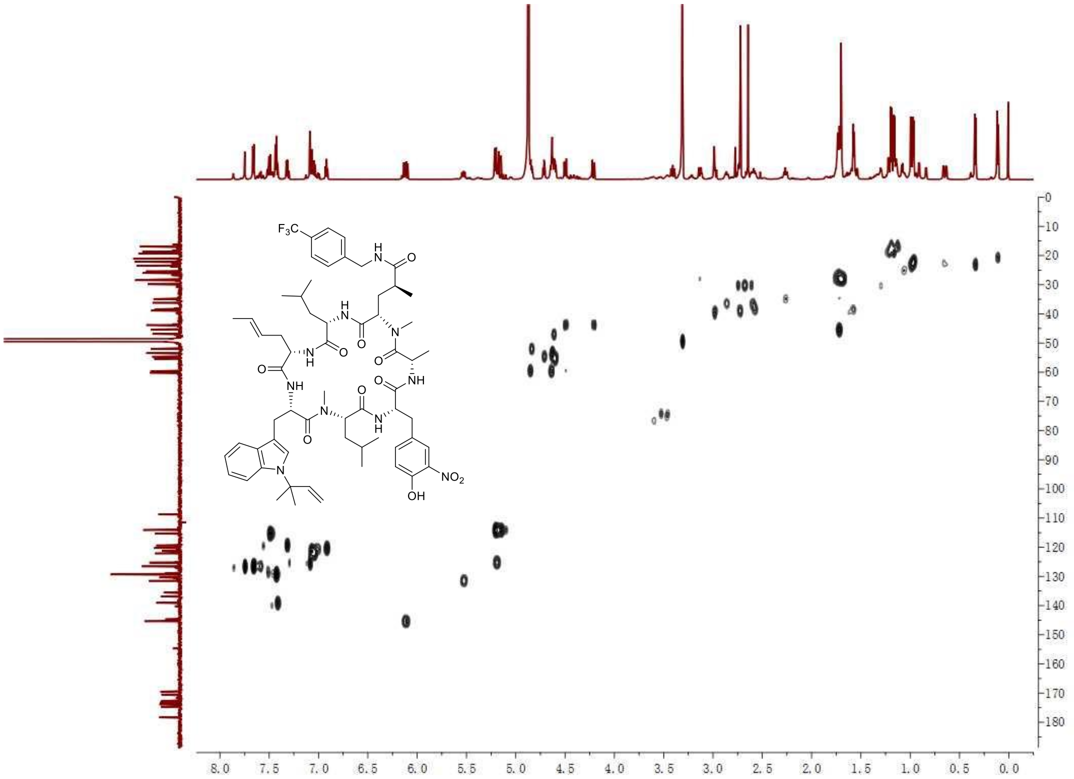

Figure S36. HSQC spectrum of compound **12** in CD_3OD

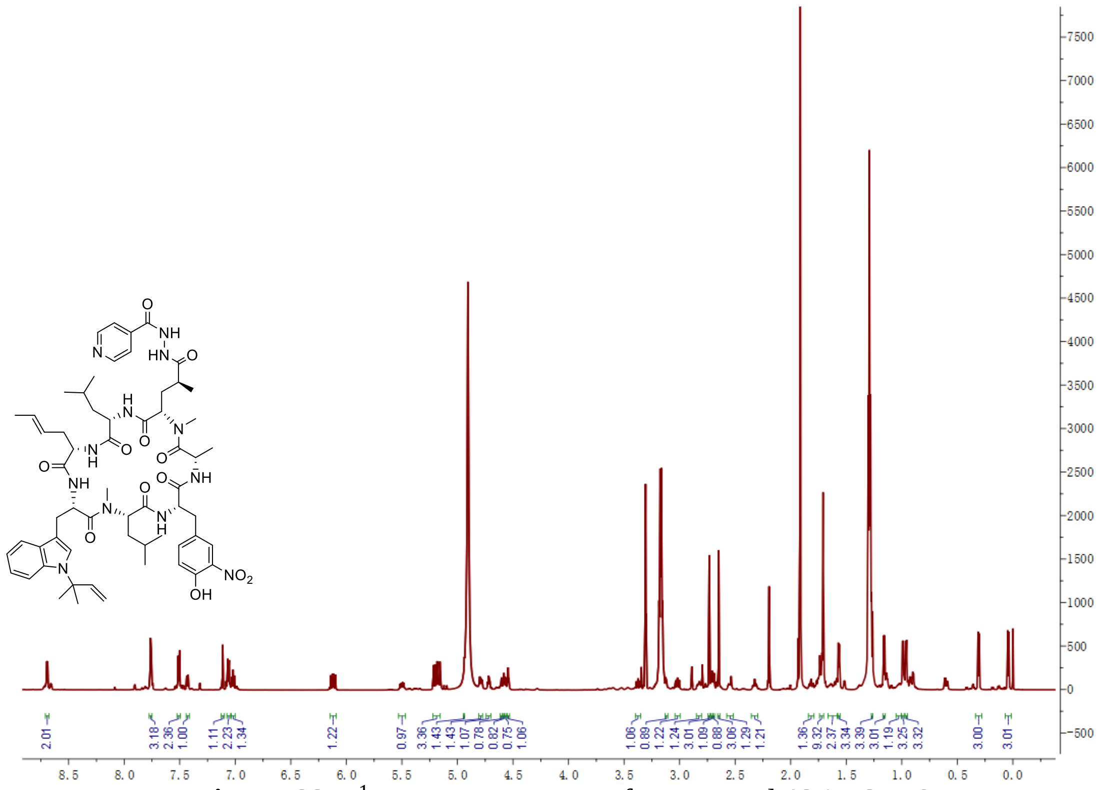

Figure S37. ^{1}H NMR spectrum of compound **13** in CD_3OD

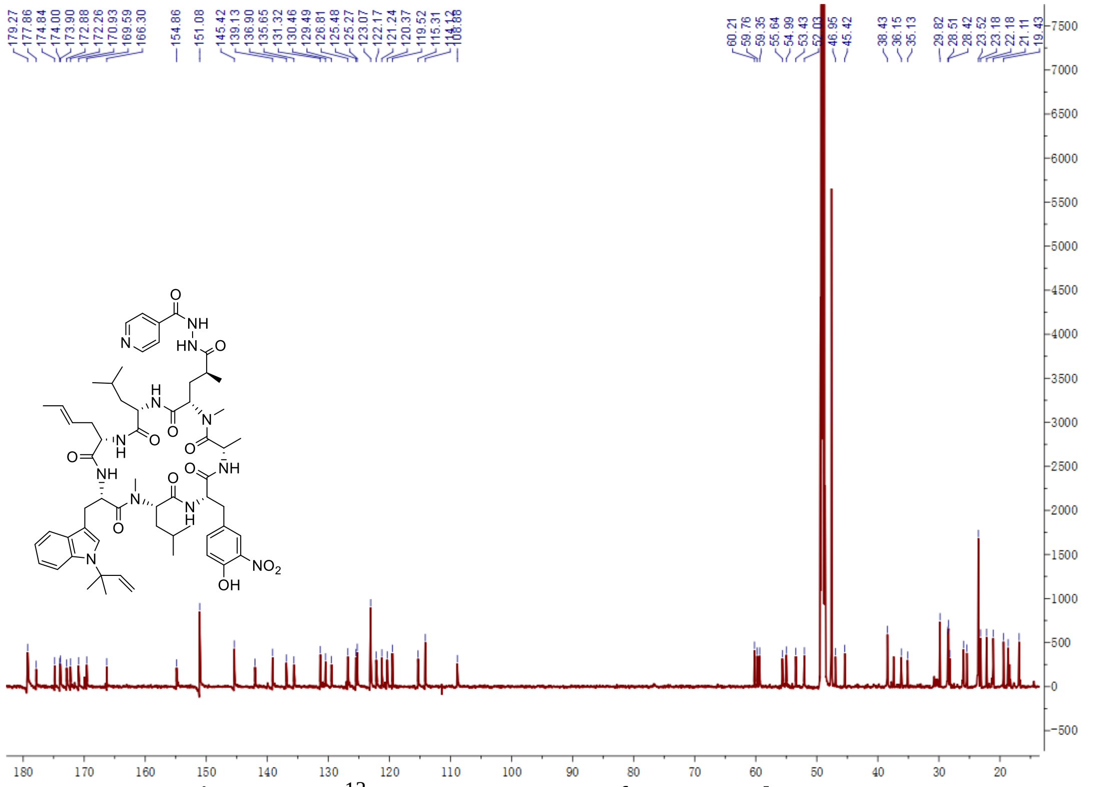

Figure S38. ^{13}C NMR spectrum of compound **13** in CD_3OD

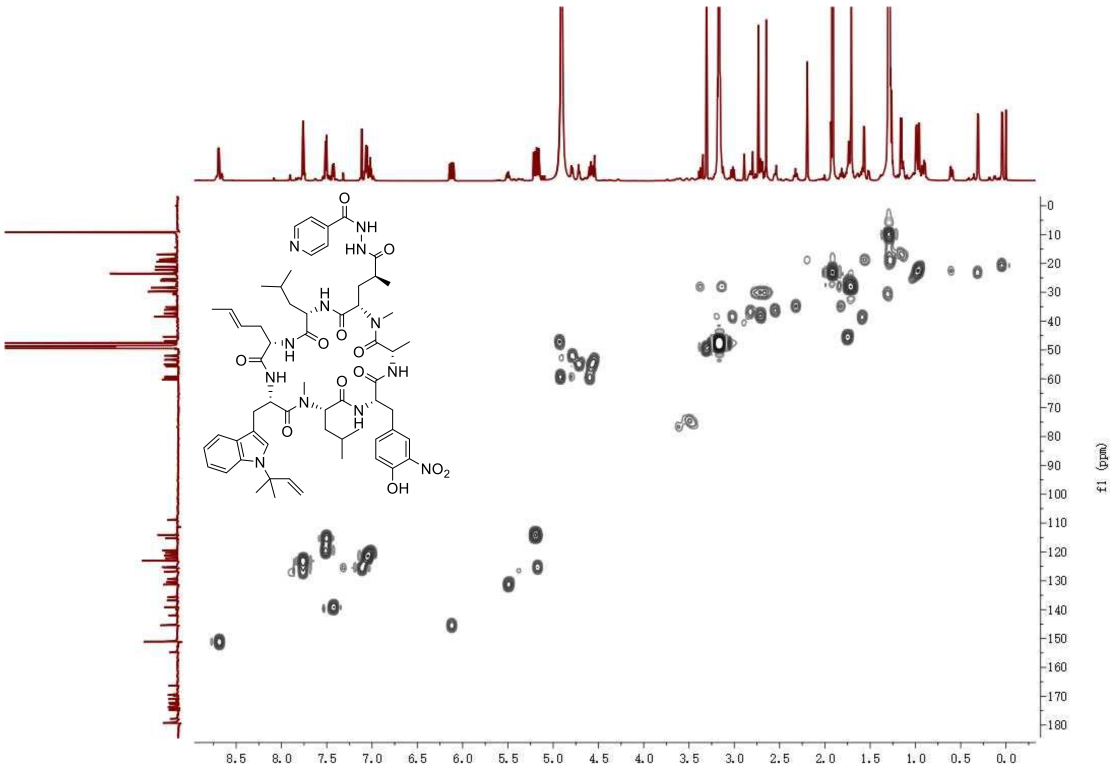

Figure S39. HSQC spectrum of compound **13** in CD_3OD

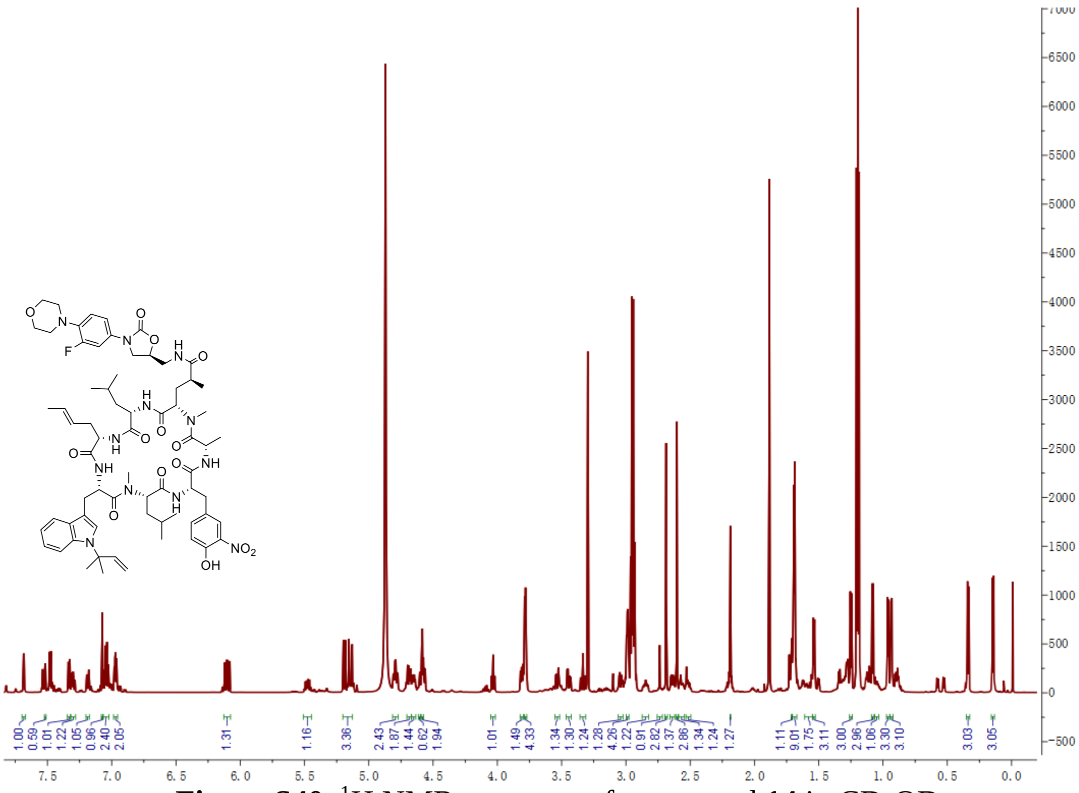

Figure S40. ^{1}H NMR spectrum of compound **14** in CD_3OD

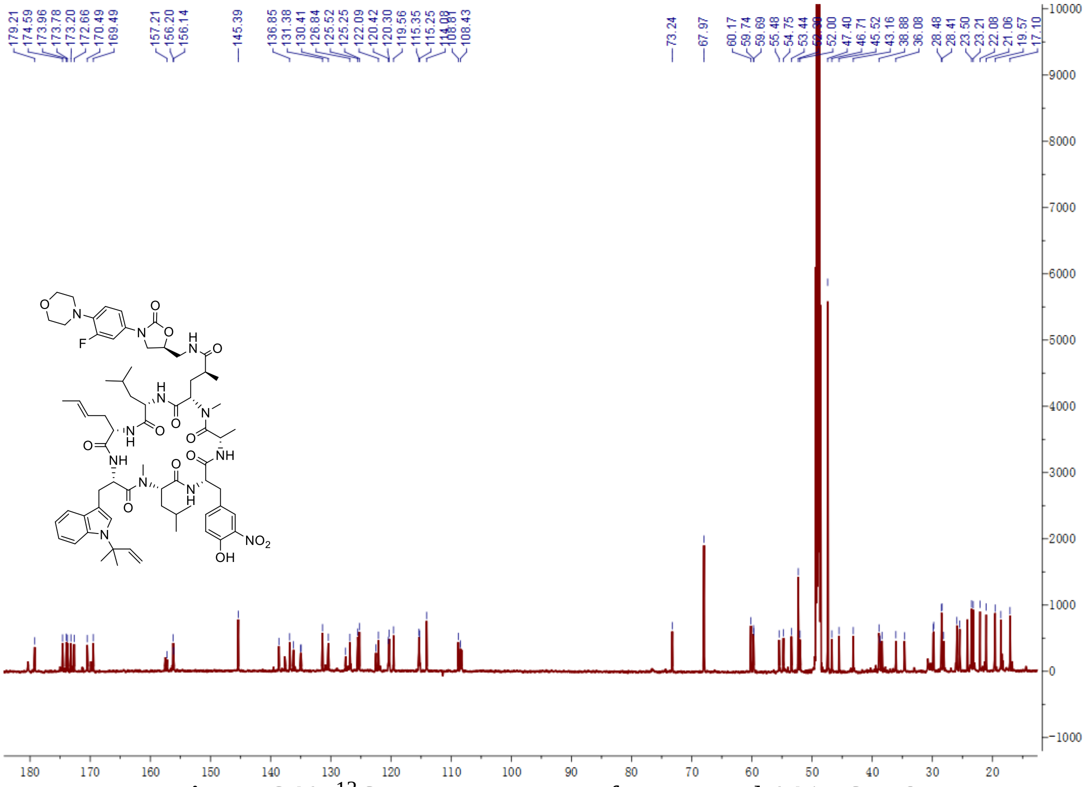

Figure S41. ^{13}C NMR spectrum of compound **14** in CD_3OD

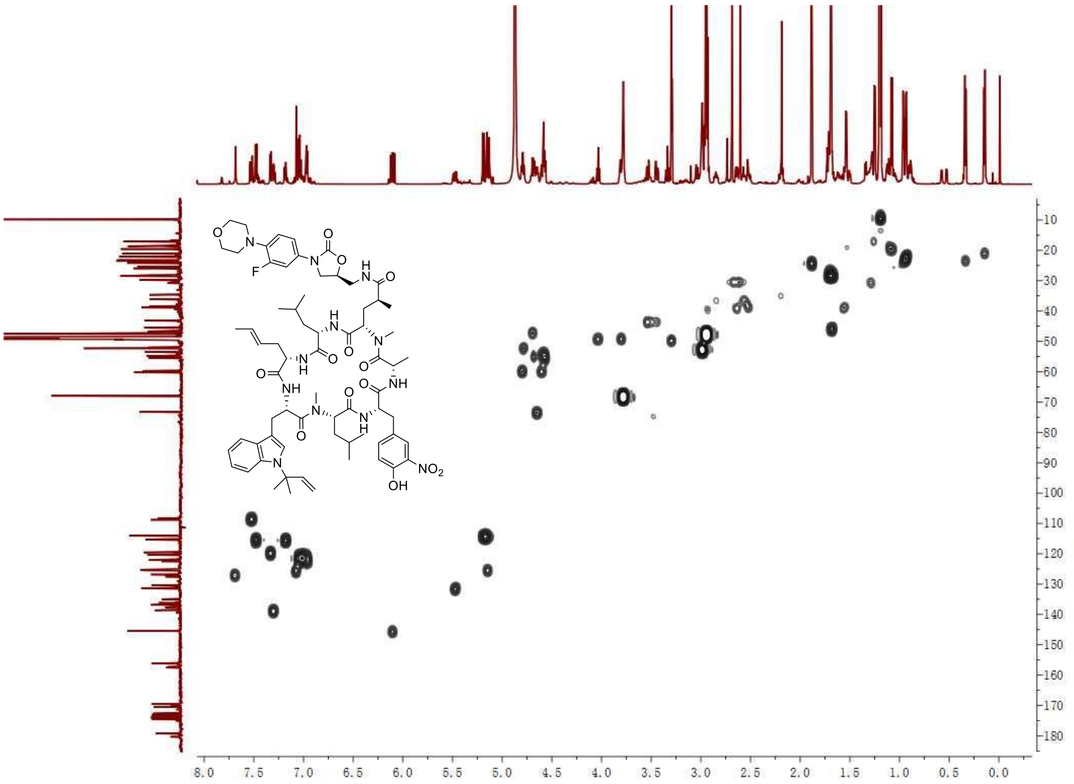

Figure S42. HSQC spectrum of compound **14** in CD_3OD

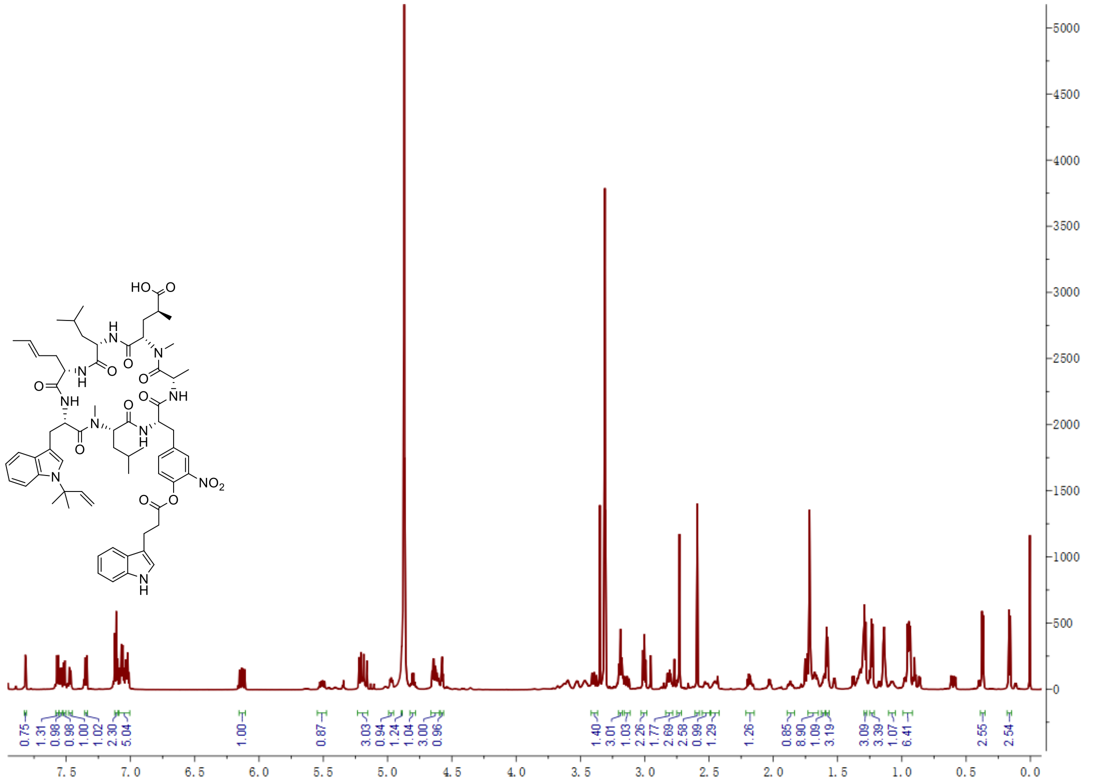

Figure S43. ^{1}H NMR spectrum of compound **15** in CD_3OD

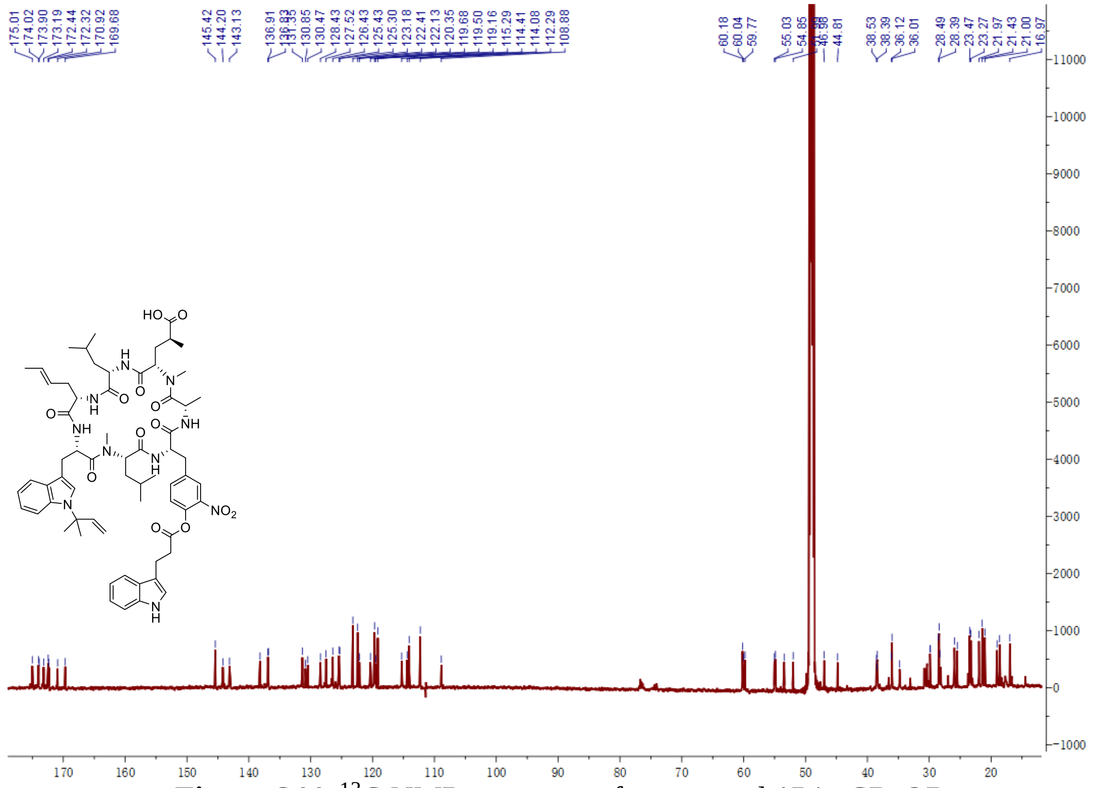

Figure S44. ^{13}C NMR spectrum of compound **15** in CD_3OD

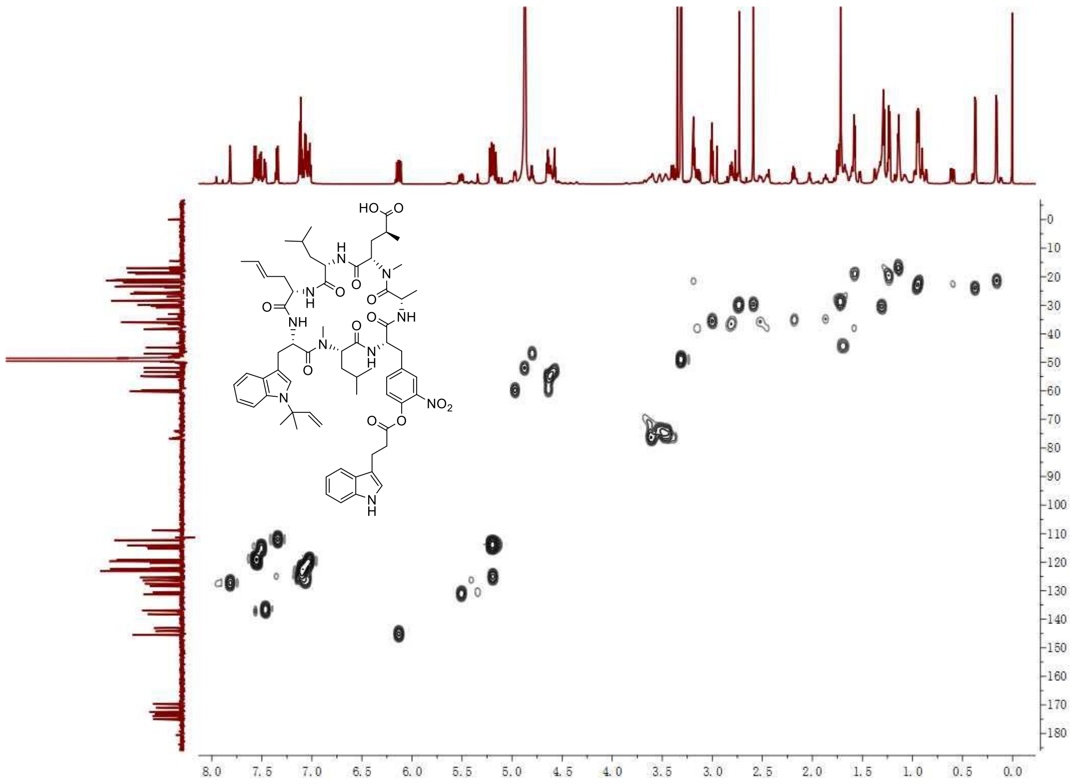

Figure S45. HSQC spectrum of compound **15** in CD_3OD

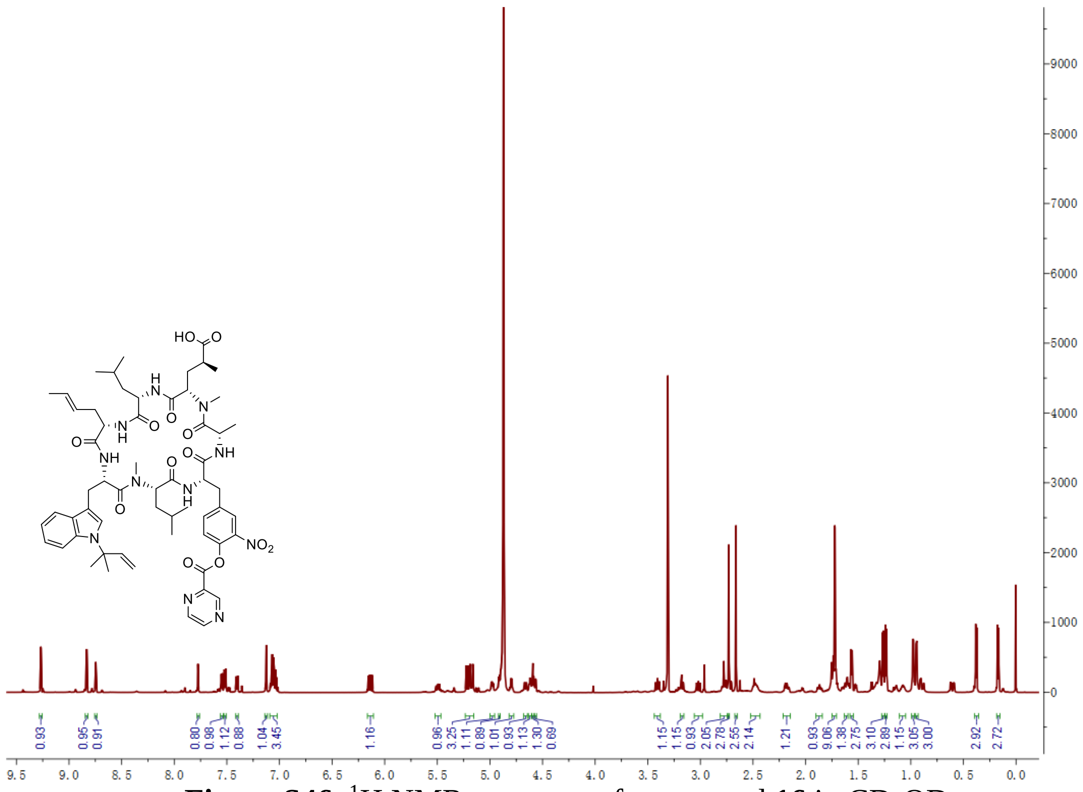

Figure S46. ^{1}H NMR spectrum of compound **16** in CD_3OD

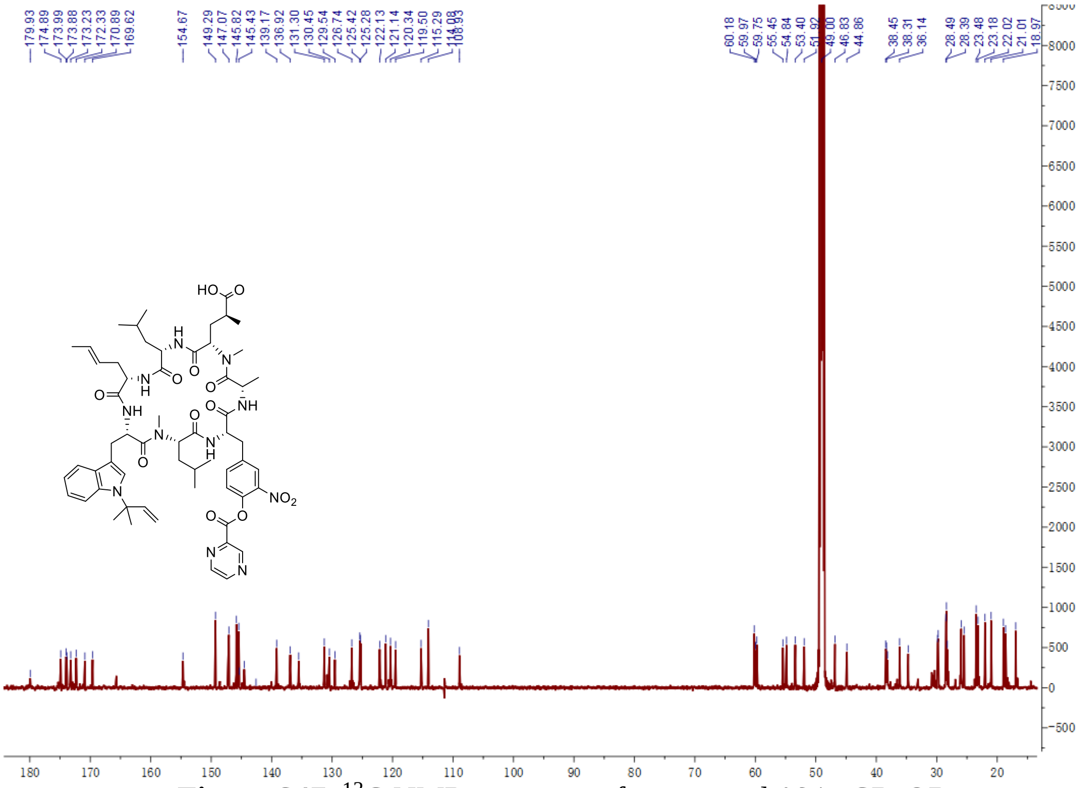

Figure S47. ^{13}C NMR spectrum of compound **16** in CD_3OD

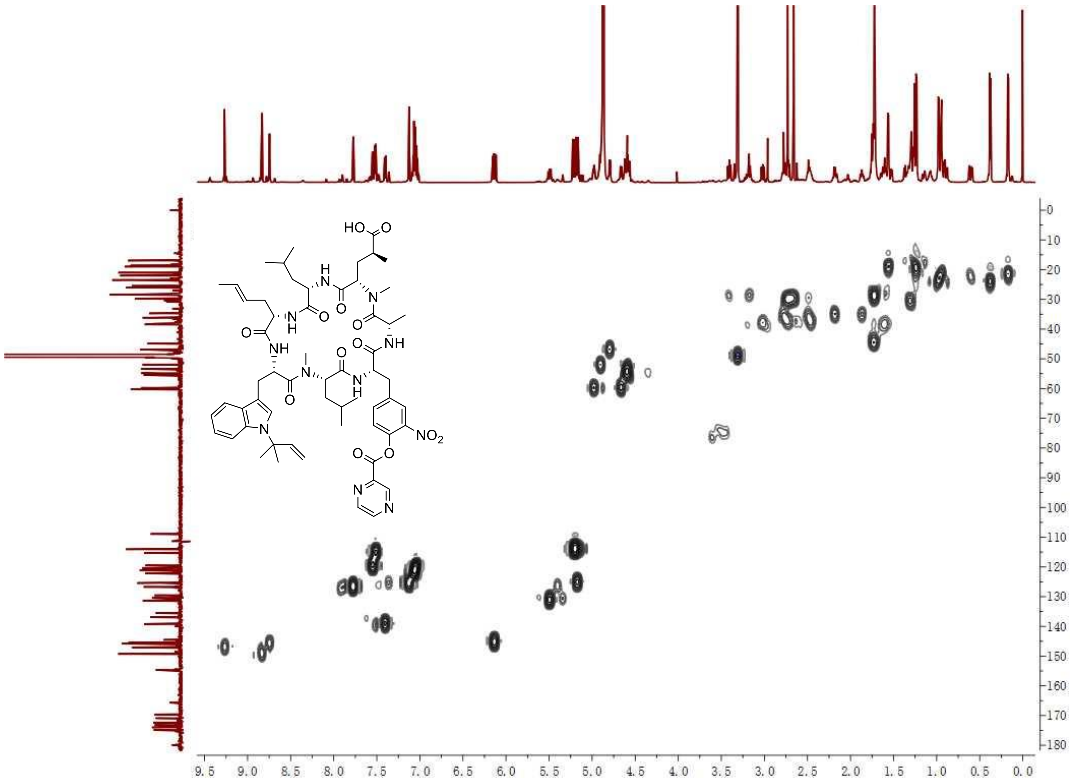

Figure S48. HSQC spectrum of compound **16** in CD_3OD

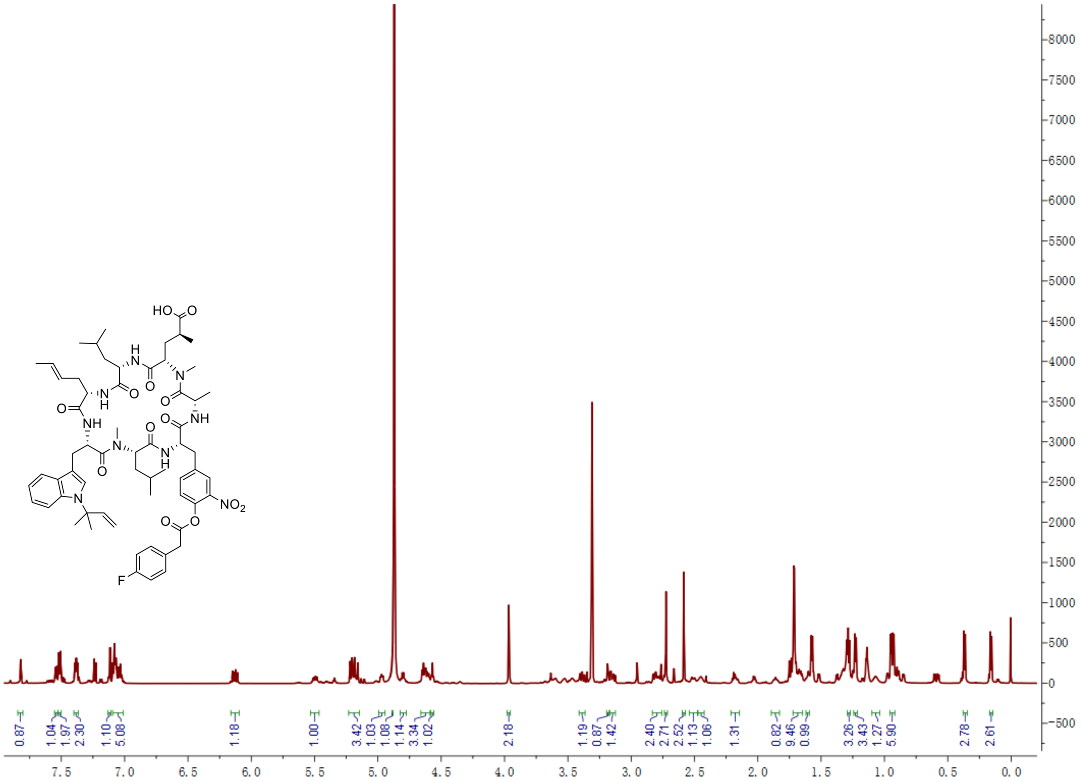

Figure S49. ^{1}H NMR spectrum of compound **17** in CD_3OD

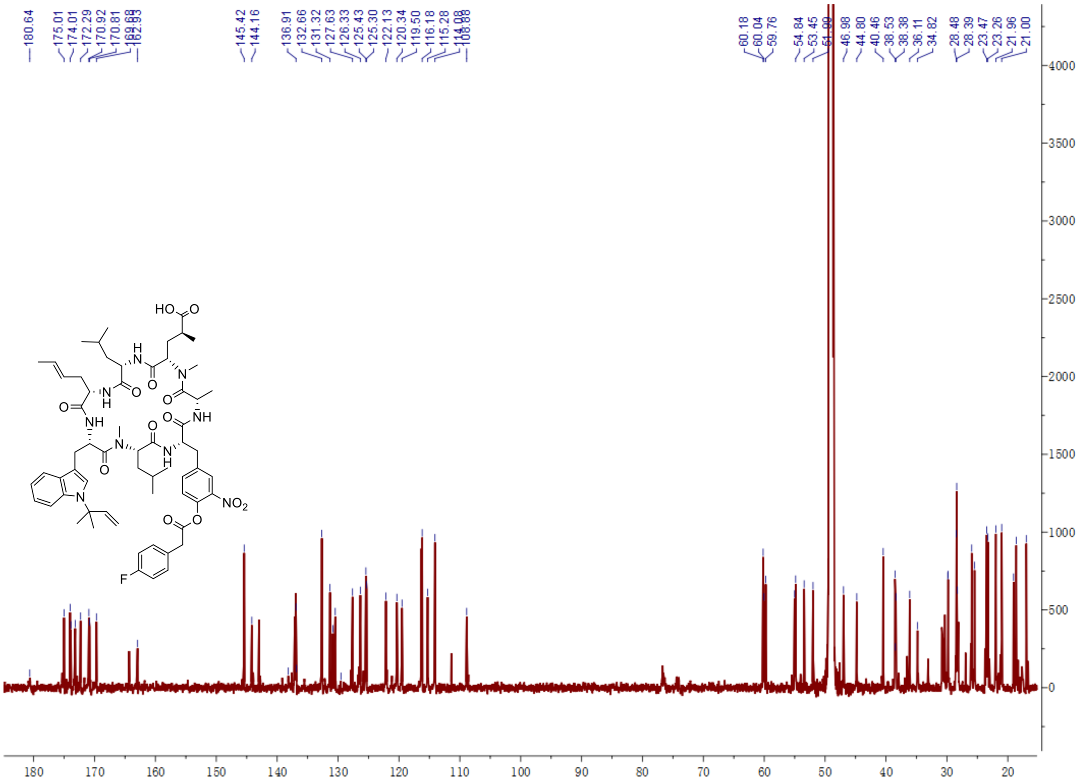

Figure S50. ^{13}C NMR spectrum of compound **17** in CD_3OD

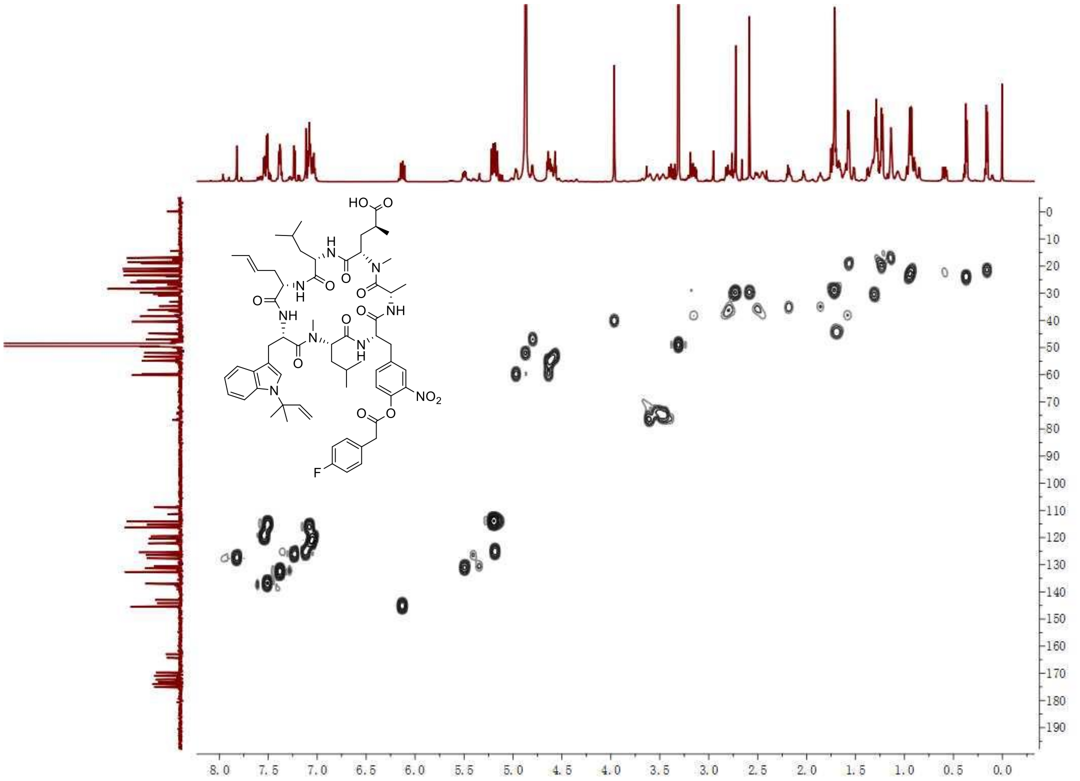

Figure S51. HSQC spectrum of compound **17** in CD_3OD

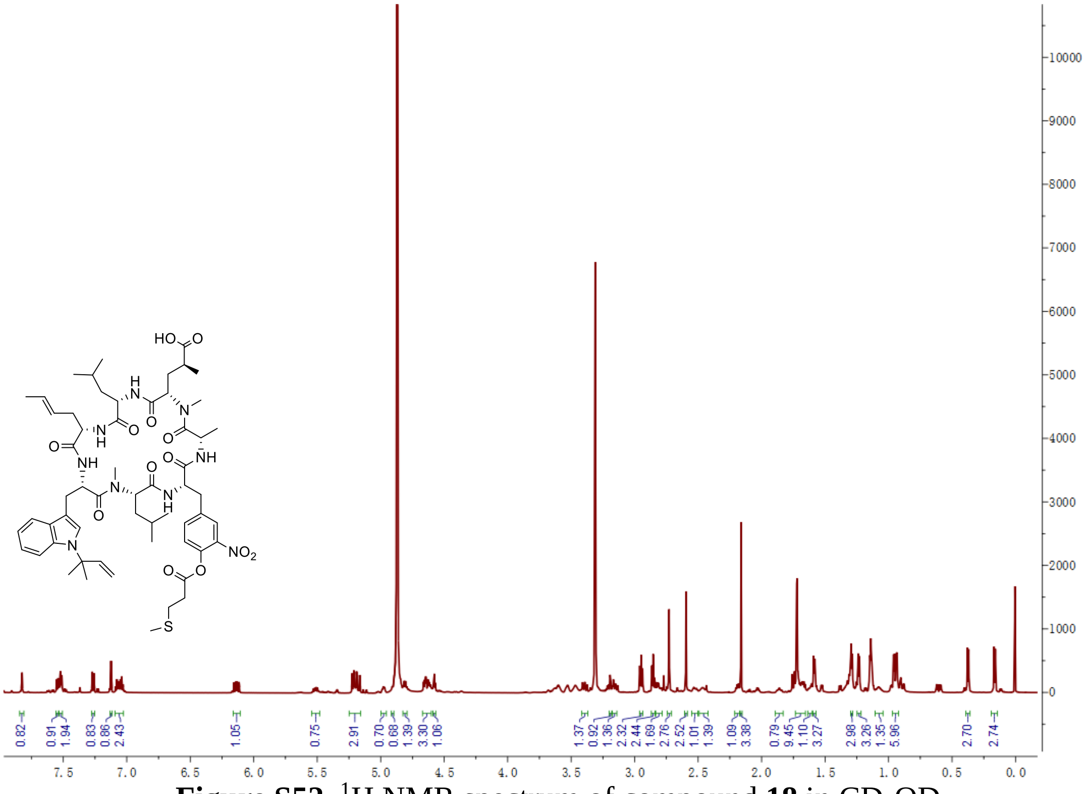

Figure S52. ^{1}H NMR spectrum of compound **18** in CD_3OD

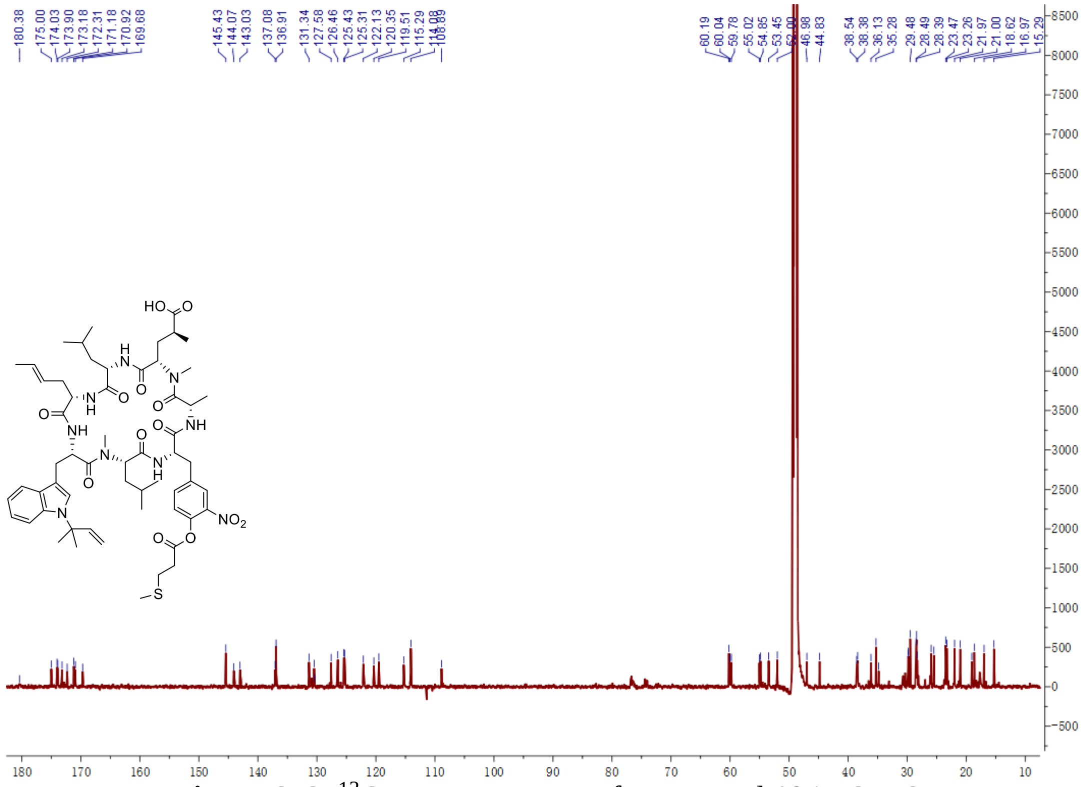

Figure S53. ^{13}C NMR spectrum of compound **18** in CD_3OD

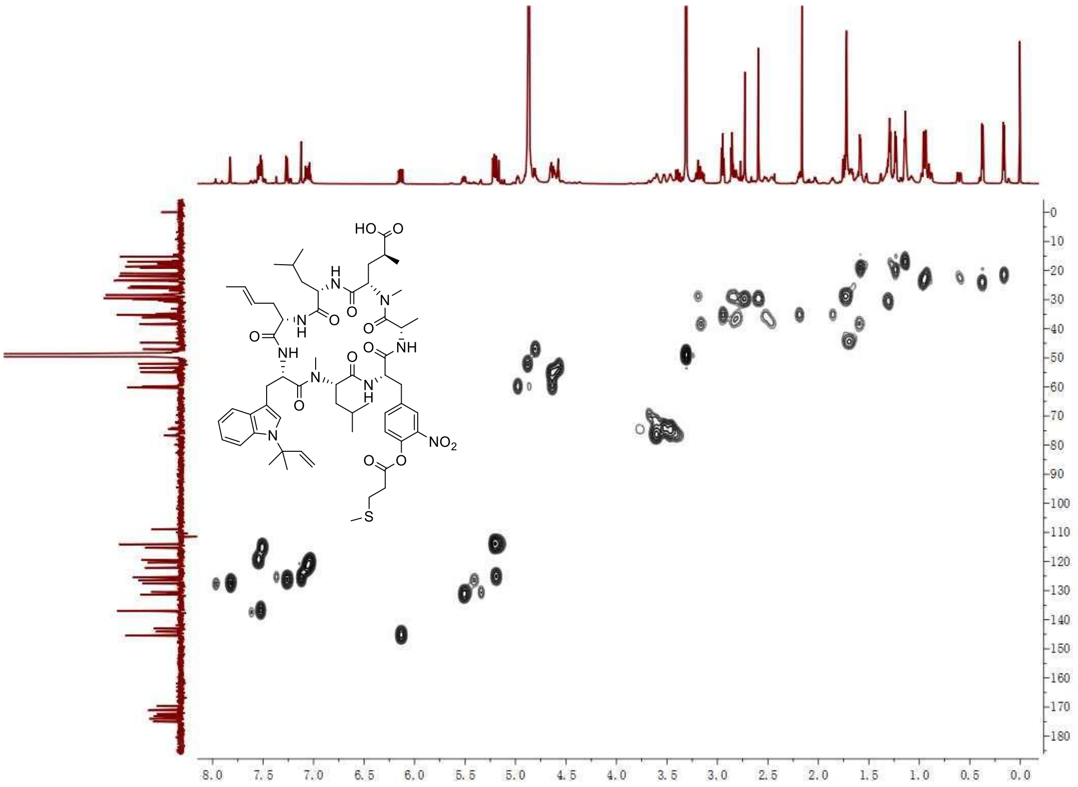

Figure S54. HSQC spectrum of compound **18** in CD_3OD

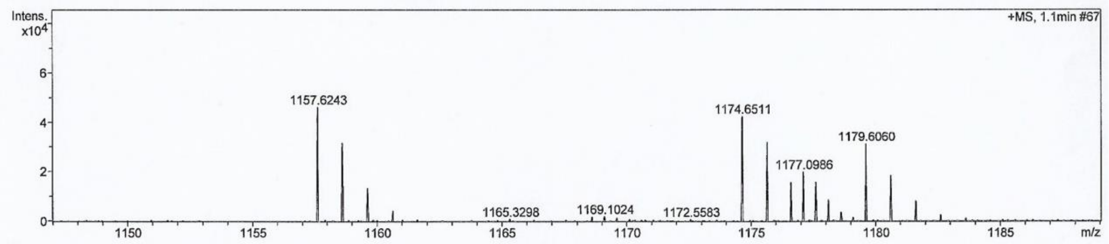

Meas. m/z	#	Ion Formula	Score	m/z	err [ppm]	err [mDa]	mSigma	rdb	e⁻ Conf	N-Rule
1157.6243	1	C59H85N10O14	100.00	1157.6241	-0.2	-0.2	15.8	22.5	even	ok
1179.6060	1	C59H84N10NaO14	100.00	1179.6061	-0.1	-0.1	46.9	22.5	even	ok

Figure S55. HRMS of compound **1**

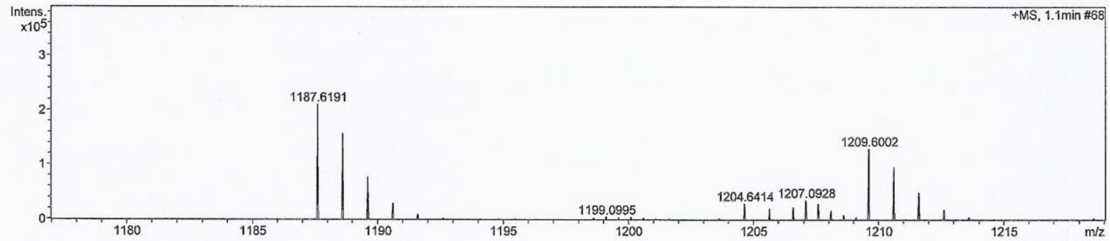

Meas. m/z	#	Ion Formula	Score	m/z	err [ppm]	err [mDa]	mSigma	rdb	e⁻ Conf	N-Rule
1187.6191	1	C60H87N10O13S	100.00	1187.6169	-1.9	-2.2	32.5	22.5	even	ok
1209.6002	1	C60H86N10NaO13S	100.00	1209.5989	1.1	1.3	36.2	22.5	even	ok

Figure S56. HRMS of compound **2**

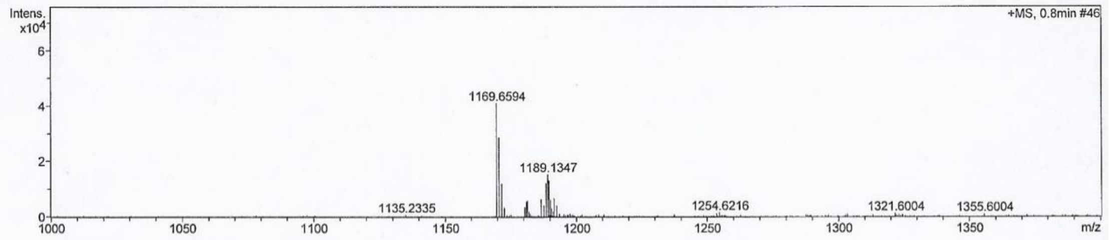

Meas. m/z	#	Ion Formula	Score	m/z	err [ppm]	err [mDa]	mSigma	rdb	e⁻ Conf	N-Rule
1169.6594	1	C61H89N10O13	100.00	1169.6605	-1.0	-1.1	9.5	22.5	even	ok
1191.6435	1	C61H88N10NaO13	100.00	1191.6425	-0.9	-1.1	59.5	22.5	even	ok

Figure S57. HRMS of compound **3**

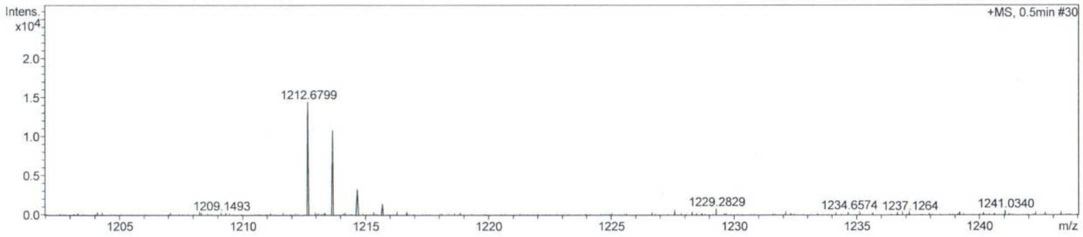

Meas. m/z	#	Ion Formula	Score	m/z	err [ppm]	err [mDa]	mSigma	rdb	e⁻ Conf	N-Rule
1212.6799	1	C61H90N13O13	100.00	1212.6776	1.9	2.3	28.0	23.5	even	ok
1234.6574	1	C61H89N13NaO13	100.00	1234.6595	1.7	2.2	149.1	23.5	even	ok

Figure S58. HRMS of compound **4**

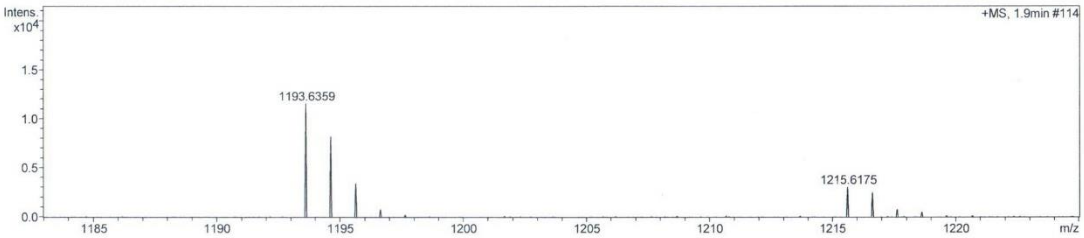

Meas. m/z	#	Ion Formula	Score	m/z	err [ppm]	err [mDa]	mSigma	rdb	e⁻ Conf	N-Rule
1193.6359	1	C61H85N12O13	100.00	1193.6354	0.4	0.5	8.9	25.5	even	ok
1215.6175	1	C61H84N12NaO13	100.00	1215.6173	-0.1	-0.2	59.8	25.5	even	ok

Figure S59. HRMS of compound **5**

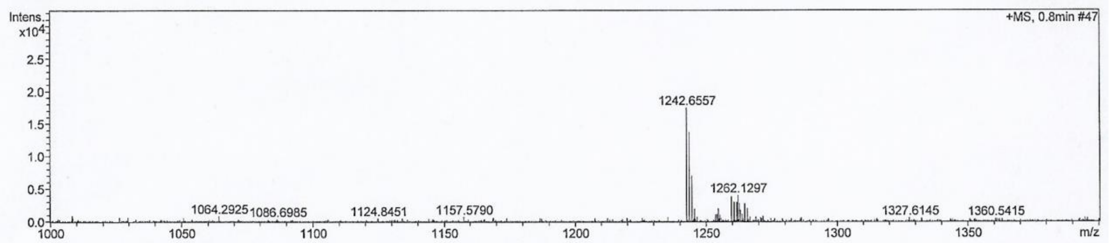

Meas. m/z	#	Ion Formula	Score	m/z	err [ppm]	err [mDa]	mSigma	rdb	e⁻ Conf	N-Rule
1242.6557	1	C66H88N11O13	100.00	1242.6558	0.0	0.0	36.9	28.5	even	ok
1264.6364	1	C66H87N11NaO13	100.00	1264.6377	-1.0	-1.3	23.3	28.5	even	ok

Figure S60. HRMS of compound **6**

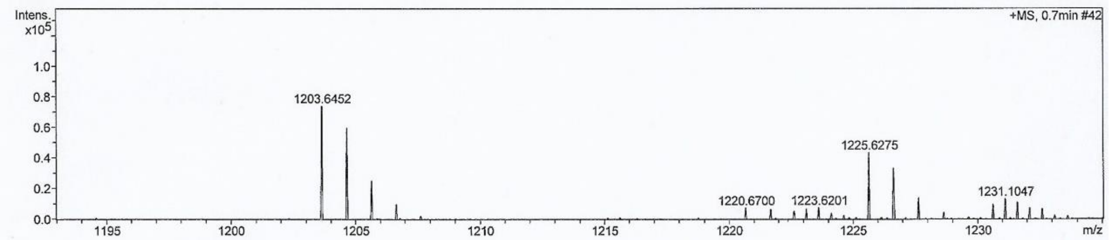

Meas. m/z	#	Ion Formula	Score	m/z	err [ppm]	err [mDa]	mSigma	rdb	e⁻ Conf	N-Rule
1203.6452	1	C64H87N10O13	100.00	1203.6449	-0.3	-0.4	36.8	26.5	even	ok
1225.6275	1	C64H86N10NaO13	100.00	1225.6268	-0.5	-0.7	17.5	26.5	even	ok

Figure S61. HRMS of compound **7**

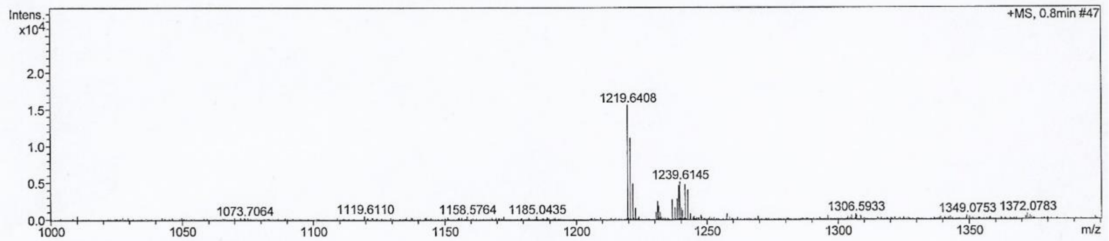

Meas. m/z	#	Ion Formula	Score	m/z	err [ppm]	err [mDa]	mSigma	rdb	e⁻ Conf	N-Rule
1219.6408	1	C64H87N10O14	100.00	1219.6398	-0.9	-1.1	15.6	26.5	even	ok
1241.6205	1	C64H86N10NaO14	100.00	1241.6217	1.0	1.2	77.3	26.5	even	ok

Figure S62. HRMS of compound **8**

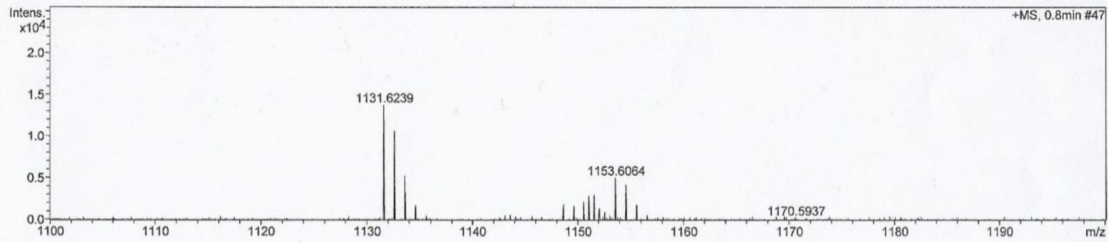

Meas. m/z	#	Ion Formula	Score	m/z	err [ppm]	err [mDa]	mSigma	rdb	e⁻ Conf	N-Rule
1131.6239	1	C61H83N10O11	100.00	1131.6237	-0.2	-0.2	65.0	25.5	even	ok
1153.6064	1	C61H82N10NaO11	100.00	1153.6057	0.6	0.7	80.1	25.5	even	ok

Figure S63. HRMS of compound **9**

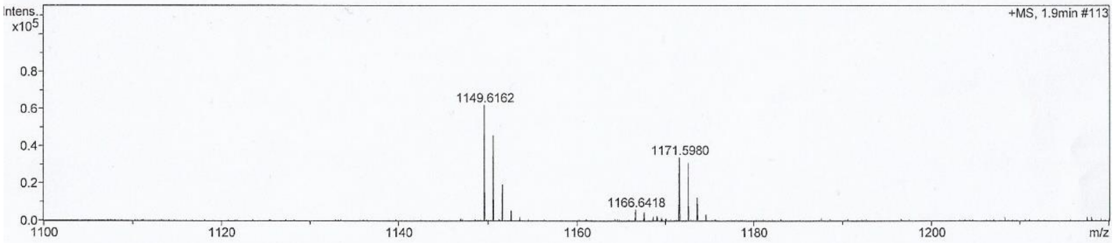

Meas. m/z	#	Ion Formula	Score	m/z	err [ppm]	err [mDa]	mSigma	rdb	e⁻ Conf	N-Rule
1149.6162	1	C61H82FN10O11	100.00	1149.6143	1.6	1.9	23.4	25.5	even	ok
1171.5980	1	C61H81FN10NaO11	100.00	1171.5963	1.5	1.7	101.5	25.5	even	ok

Figure S64. HRMS of compound **10**

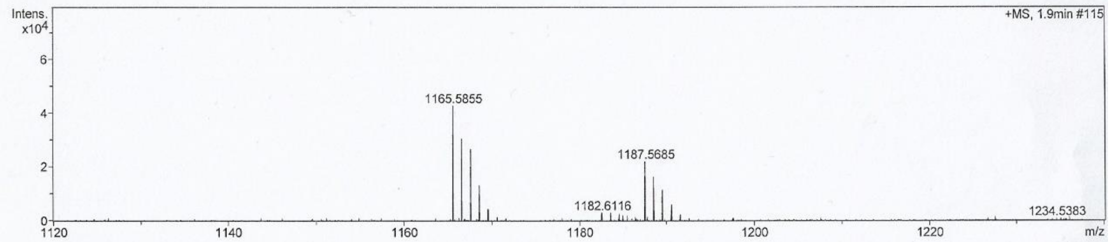

Meas. m/z	#	Ion Formula	Score	m/z	err [ppm]	err [mDa]	mSigma	rdb	e⁻ Conf	N-Rule
1165.5855	1	C61H82ClN10O11	100.00	1165.5848	-0.7	-0.8	13.6	25.5	even	ok
1187.5685	1	C61H81ClN10NaO11	100.00	1187.5667	-1.5	-1.8	31.9	25.5	even	ok

Figure S65. HRMS of compound **11**

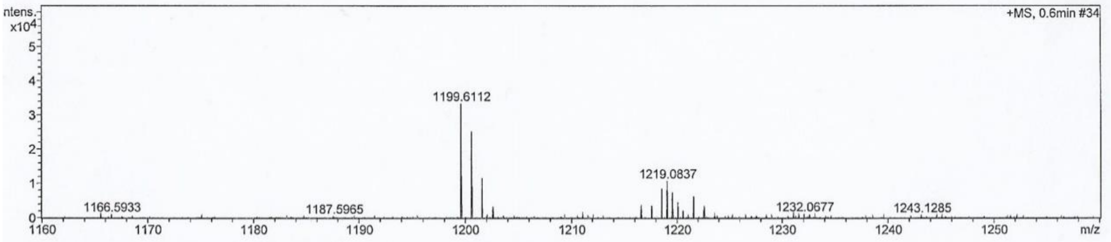

Meas. m/z	#	Ion Formula	Score	m/z	err [ppm]	err [mDa]	mSigma	rdb	e⁻ Conf	N-Rule
1199.6112	1	C62H82F3N10O11	100.00	1199.6111	-0.1	-0.1	37.3	25.5	even	ok
1221.5910	1	C62H81F3N10NaO11	100.00	1221.5931	1.7	2.0	84.4	25.5	even	ok

Figure S66. HRMS of compound **12**

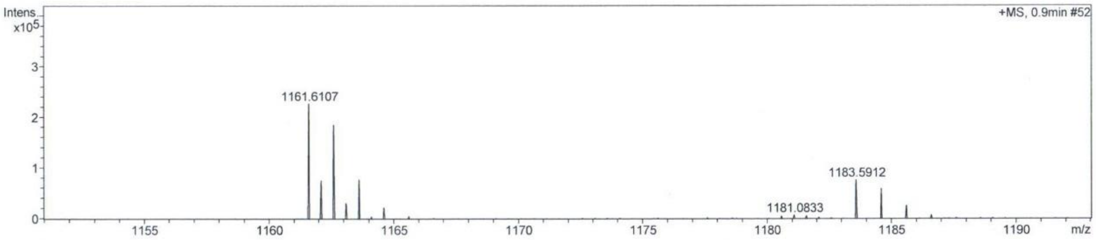

Meas. m/z	#	Ion Formula	Score	m/z	err [ppm]	err [mDa]	mSigma	rdb	e⁻ Conf	N-Rule
1161.6107	1	C60H81N12O12	100.00	1161.6091	1.4	1.6	58.2	26.5	even	ok
1183.5912	1	C62H79N12O12	100.00	1183.5935	1.9	2.3	35.7	29.5	even	ok

Figure S67. HRMS of compound **13**

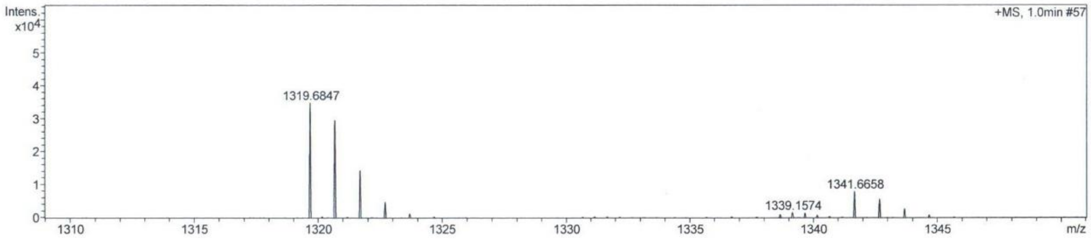

Meas. m/z	#	Ion Formula	Score	m/z	err [ppm]	err [mDa]	mSigma	rdb	e⁻ Conf	N-Rule
1319.6847	1	C68H92FN12O14	100.00	1319.6835	-0.9	-1.2	38.2	28.5	even	ok
1341.6658	1	C68H91FN12NaO14	100.00	1341.6654	-0.3	-0.4	33.7	28.5	even	ok

Figure S68. HRMS of compound **14**

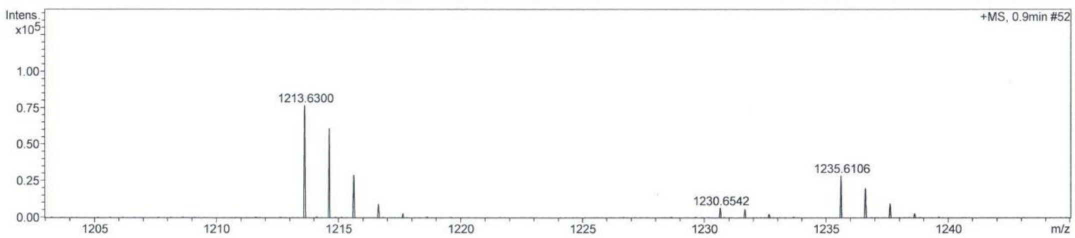

Meas. m/z	#	Ion Formula	Score	m/z	err [ppm]	err [mDa]	mSigma	rdb	e⁻ Conf	N-Rule
1213.6300	1	C65H85N10O13	100.00	1213.6292	-0.7	-0.8	36.5	28.5	even	ok
1235.6106	1	C65H84N10NaO13	100.00	1235.6112	-0.5	-0.6	24.0	28.5	even	ok

Figure S69. HRMS of compound **15**

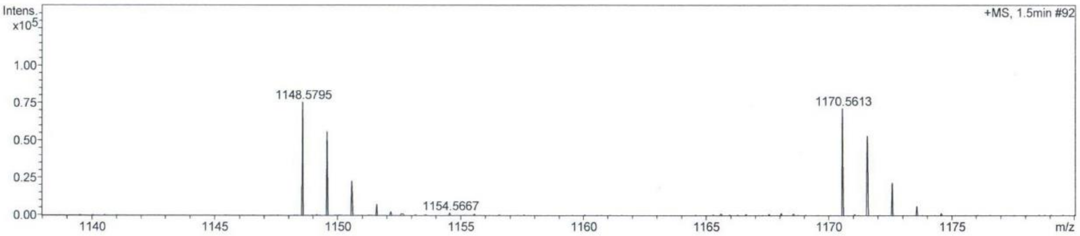

Meas. m/z	#	Ion Formula	Score	m/z	err [ppm]	err [mDa]	mSigma	rdb	e⁻ Conf	N-Rule
1148.5795	1	C59H78N11O13	100.00	1148.5775	1.7	2.0	29.4	26.5	even	ok
1170.5613	1	C59H77N11NaO13	100.00	1170.5595	-1.6	-1.8	30.2	26.5	even	ok

Figure S70. HRMS of compound **16**

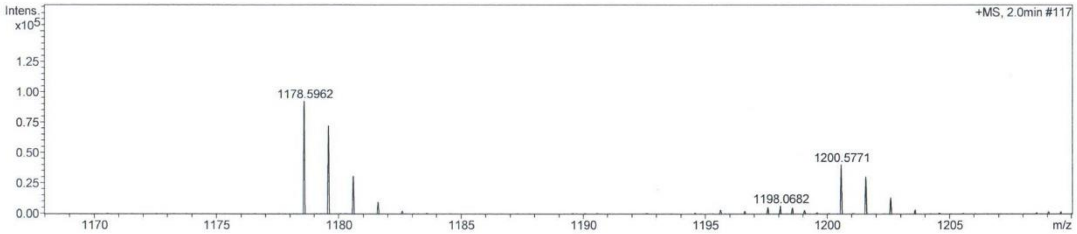

Meas. m/z	#	Ion Formula	Score	m/z	err [ppm]	err [mDa]	mSigma	rdb	e⁻ Conf	N-Rule
1178.5962	1	C62H81FN9O13	100.00	1178.5932	-2.5	-3.0	39.1	26.5	even	ok
1200.5771	1	C62H80FN9NaO13	100.00	1200.5752	1.6	1.9	29.3	26.5	even	ok

Figure S71. HRMS of compound **17**

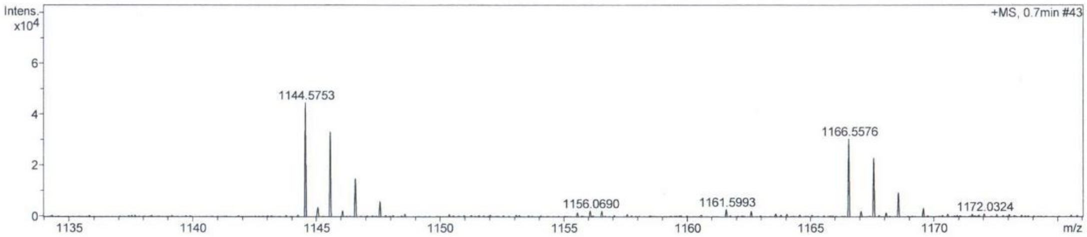

Meas. m/z	#	Ion Formula	Score	m/z	err [ppm]	err [mDa]	mSigma	rdb	e⁻ Conf	N-Rule
1144.5753	1	C58H82N9O13S	100.00	1144.5747	-0.5	-0.6	30.6	22.5	even	ok
1166.5576	1	C58H81N9NaO13S	100.00	1166.5567	0.8	0.9	32.5	22.5	even	ok

Figure S72. HRMS of compound **18**
